# Oral 11β-HSD1 inhibitor AZD4017 improves wound healing and skin integrity in adults with type 2 diabetes mellitus: a pilot randomized controlled trial

**DOI:** 10.1530/EJE-21-1197

**Published:** 2022-02-03

**Authors:** R A Ajjan, E M A Hensor, F Del Galdo, K Shams, A Abbas, R J Fairclough, L Webber, L Pegg, A Freeman, A E Taylor, W Arlt, A W Morgan, A A Tahrani, P M Stewart, D A Russell, A Tiganescu

**Affiliations:** 1 Leeds Institute of Cardiovascular and Metabolic Medicine, University of Leeds, Leeds, UK; 2 Leeds Institute of Rheumatic and Musculoskeletal Medicine, University of Leeds, Leeds, UK; 3 NIHR Leeds Biomedical Research Center, Leeds Teaching Hospitals, NHS Trust, Leeds, UK; 4 Emerging Innovations Unit, Discovery Sciences, BioPharmaceuticals R&D; 5 Emerging Portfolio Development, Late Oncology, Oncology R&D, AstraZeneca, Cambridge, UK; 6 Institute of Metabolism and Systems Research, University of Birmingham, Birmingham, UK; 7 Centre for Endocrinology, Diabetes and Metabolism, Birmingham Health Partners, Birmingham, UK; 8 NIHR Birmingham Biomedical Research Centre, University Hospitals Birmingham NHS Foundation Trust, Birmingham, UK; 9 Faculty of Medicine and Health, University of Leeds, Leeds, UK; 10 Leeds Vascular Institute, Leeds Teaching Hospitals NHS Trust, Leeds, UK

## Abstract

**Background:**

Chronic wounds (e.g. diabetic foot ulcers) reduce the quality of life, yet treatments remain limited. Glucocorticoids (activated by the enzyme 11β-hydroxysteroid dehydrogenase type 1, 11β-HSD1) impair wound healing.

**Objectives:**

Efficacy, safety, and feasibility of 11β-HSD1 inhibition for skin function and wound healing.

**Design:**

Investigator-initiated, double-blind, randomized, placebo-controlled, parallel-group phase 2b pilot trial.

**Methods:**

Single-center secondary care setting. Adults with type 2 diabetes mellitus without foot ulcers were administered 400 mg oral 11β-HSD1 inhibitor AZD4017 (*n* = 14) or placebo (*n* = 14) bi-daily for 35 days. Participants underwent 3-mm full-thickness punch skin biopsies at baseline and on day 28; wound healing was monitored after 2 and 7 days. Computer-generated 1:1 randomization was pharmacy-administered. Analysis was descriptive and focused on CI estimation. Of the 36 participants screened, 28 were randomized.

**Results:**

Exploratory proof-of-concept efficacy analysis suggested AZD4017 did not inhibit 24-h *ex vivo*skin 11β-HSD1 activity (primary outcome; difference in percentage conversion per 24 h 1.1% (90% CI: −3.4 to 5.5) but reduced systemic 11β-HSD1 activity by 87% (69–104%). Wound diameter was 34% (7–63%) smaller with AZD4017 at day 2, and 48% (12–85%) smaller after repeat wounding at day 30. AZD4017 improved epidermal integrity but modestly impaired barrier function. Minimal adverse events were comparable to placebo. Recruitment rate, retention, and data completeness were 2.9/month, 27/28, and 95.3%, respectively.

**Conclusion:**

A phase 2 trial is feasible, and preliminary proof-of-concept data suggests AZD4017 warrants further investigation in conditions of delayed healing, for example in diabetic foot ulcers.

**Significance statement:**

Stress hormone activation by the enzyme 11β-HSD type 1 impairs skin function (e.g. integrity) and delays wound healing in animal models of diabetes, but effects in human skin were previously unknown. Skin function was evaluated in response to treatment with a 11β-HSD type 1 inhibitor (AZD4017), or placebo, in people with type 2 diabetes. Importantly, AZD4017 was safe and well tolerated. This first-in-human randomized, controlled, clinical trial found novel evidence that 11β-HSD type 1 regulates skin function in humans, including improved wound healing, epidermal integrity, and increased water loss. Results warrant further studies in conditions of impaired wound healing, for example, diabetic foot ulcers to evaluate 11β-HSD type 1 as a novel therapeutic target forchronic wounds.

## Introduction

Chronic, non-healing wounds (e.g. diabetic foot ulcers) are a common and worldwide health problem of substantial medical and socioeconomic importance (1). Costs for wound care in the UK are estimated to be £2.03 to £3.8 million per 100 000 population (2, 3). The profound atrophic effects of glucocorticoids (GC) on human skin structure and function are well documented, including increased transepidermal water loss (TEWL), skin thinning, and delayed wound healing (4, 5, 6, 7, 8, 9, 10, 11). 11β-hydroxysteroid dehydrogenase (11β-HSD) isozymes regulate local GC availability independently of circulating levels (12). In the skin, 11β-HSD1 is widely expressed and induced by GC in a forward-feedback manner (13, 14).

11β-HSD1 activity increases during wound healing (15) and 11β-HSD1 inhibition improves wound healing in preclinical models of GC excess, aging, and diabetes (13, 16, 17, 18). Therefore, 11β-HSD1 inhibition may aid wound healing, particularly in conditions associated with increased systemic GC levels, such as diabetes (19, 20). However, the ability of systemic 11β-HSD1 inhibitors to regulate skin function in humans is unknown.

We conducted this randomized controlled trial to evaluate the effect of 11β-HSD1 inhibition on skin function and wound healing in patients with type 2 diabetes mellitus.––

## Patients and methods

We have summarized the trial methods in brief here but for full details please refer to the final approved trial protocol provided in Supplementary data 1 (see section on supplementary materials given at the end of this article).

### Study approval

Informed consent was obtained after the nature and possible consequences of the study had been explained. Full ethical approval was acquired from North West Greater Manchester Central Research Ethics Committee 17/NW/0283 before initiation of recruitment. The reported investigations were carried out in accordance with the principles of the Declaration of Helsinki as revised in 2008.

### Study design

This was a randomized, double-blind, parallel-group, placebo-controlled, phase 2 pilot trial in people with type 2 diabetes. Participants were recruited between March 28, 2018, and January 23, 2019 (first to last randomized participant telephone invitations), and the last participant’s last follow-up call (telephone discharge) was on April 3, 2019. A total of 28 participants (22 male and 6 female) were randomized, and 27 completed the 35-day oral AZD4017 or placebo treatment.

### Selection of sample size

Published guidance for pilot studies recommended a sample size of 12 participants per arm (21). To achieve this sample size, the study was designed to randomize 15 participants per arm to allow for a 20% drop-out rate.

### Data monitoring

Data monitoring was carried out during the trial by the study management team and the sponsor. Independent oversight of the study, including interim safety monitoring, was conducted by an independent data monitoring and ethics committee.

### Randomization and blinding

Participants and the study team were blinded to the randomization process, and other blinding procedures included mitigation of accidental unblinding, semi-blind interim safety analyses, and independent data monitoring and ethics committee oversight. Dosing compliance was monitored by the completion of diary cards by participants and by counting the number of tablets remaining at each study visit.

### Treatments and withdrawal criteria

Participants received either oral AZD4017 (400 mg) or matched oral placebo twice daily for 35 days. For full treatment and compliance details, and AZD4017 withdrawal criteria, please refer to the protocol (Supplementary data 1).

### Study endpoints

The primary endpoint was 24 –h 11β-HSD1 activity in skin (efficacy) at baseline and day 28. Pre-specified secondary endpoints were:

- Systemic 11β-HSD1 activity at baseline and day 35
- AZD4017 quantification in skin at day 28 and in plasma at day 35
- Safety variables at baseline and days 7, 28, 35, and 42
- Urinary cortisol to cortisone metabolite analysis at baseline and day 35 (to assess systemic GC levels)
- Skin function variables at baseline and days 2, 7, 28, 30, and 35
- Feasibility variables throughout the study

### Wound healing

All individuals underwent two full-thickness 3-mm skin punch biopsies at baseline, repeated in the contralateral arm on day 28; wound healing was assessed on days 2 and 7 post-wounding (treatment days 2, 7, 30, and 35).

### Statistical analysis

A detailed standalone statistical analysis plan (SAP) was developed and finalized before breaking of the blind and processing of primary outcome samples.

In this pilot study, the analysis was descriptive throughout, so no inferential hypotheses were formally tested. As pre-specified in the SAP, analysis followed published recommendations, which state that the focus in pilot trials should be on descriptive statistics and estimation using a range of CIs other than 95% and interpreting these using the minimum clinically important difference (22).

For each outcome, differences were adjusted for gender, age, baseline glycated hemoglobin A1c (HbA1c), and (where applicable) the baseline value of the outcome. Primary two-sided 90% CIs were supplemented with CIs ranging from 75 to 95%, in line with recommendations. Adjusted differences were obtained via regression modeling; linear regression was used unless data did not meet the model assumptions and a suitable transformation could not be found. As pre-specified in the SAP, in these instances quantile (median) regression was used instead; this estimates the difference in the medians instead of the means, makes fewer assumptions about the data, and is less sensitive to outliers than linear regression.

Because minimum clinically important differences have not been established in this population for the outcomes investigated, we additionally reported percentage differences relative to the mean or median with placebo as a guideline. Analyses were conducted in Stata 16.1 (StataCorp 2019). For further details, see Supplementary data 2.

## Results

**Figure 1:**
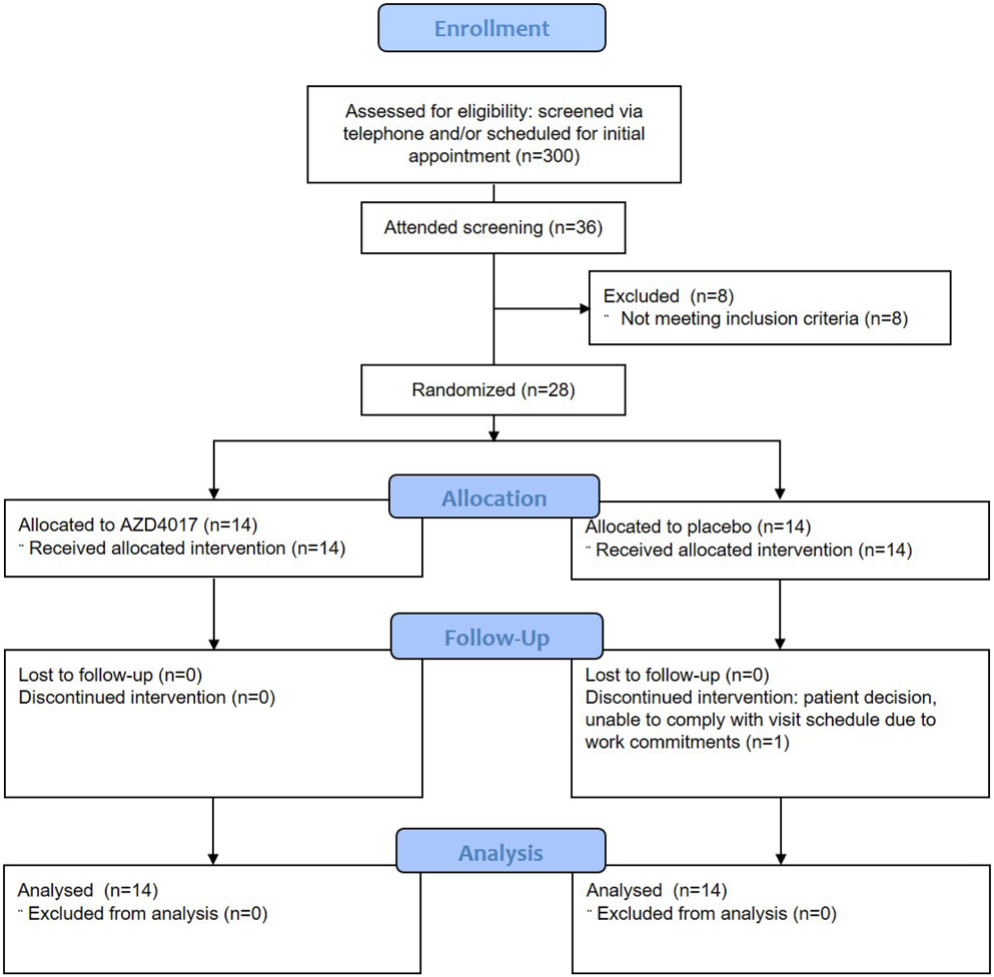
CONSORT flow diagram. Progress, from screening to study completion, of participants in the double-blind, randomized, placebo-controlled pilot trial comparing 35-day oral AZD4017 treatment with placebo in adults with type 2 diabetes. A total of 300 prospective participants were screened. Eight of the 36 individuals enrolled in the study were not randomized because they did not meet eligibility criteria, and 27 of 28 participants who were randomized completed the study.

A Consolidated Standards of Reporting Trials (CONSORT) flow diagram is presented in Fig. 1. In addition to the main findings, Supplementary data 3 presents full descriptive summaries of efficacy (Supplementary Table 1) and laboratory safety variables (Supplementary Table 2), compliance data (Supplementary Table 3), unadjusted differences in primary and secondary efficacy outcomes (Supplementary Table 4), sensitivity analyses after outlier removal (Supplementary Table 5), unadjusted and adjusted changes from baseline (Supplementary Tables 6 and 7), additional pre-specified sensitivity analyses (Supplementary Tables 8, 9, and 10), correlations between AZD4017 compliance and efficacy outcomes (Supplementary Table 11), full laboratory safety data (Supplementary Tables 12, 13, 14, and 15) and sample sizes for future trials (Supplementary Table 16).

Differences between groups are presented with 90% CI, unless otherwise stated.

### Participant demographics and baseline variables

The two randomized arms were well balanced for participant demographics, baseline efficacy, and laboratory safety variables (Table 1).

**Table 1:** Baseline demographics, primary and secondary efficacy outcomes, and safety variables of the population under study (full analysis set). Data are presented as mean ± s.d. or median (Q1 and Q3).

Variable	Placebo	AZD4017	Total
*n*	14	14	28
Demographics			
Age			
Mean ± s.d.	60.3 ± 13.4	60.1 ± 14.5	60.2 ± 13.7
Range	31–84	28–75	28–84
Male, *n*/*N* (%)	12/14 (86)	10/14 (71)	22/28 (79)
Primary outcome			
Skin 11β-HSD1 activity (% conversion/24 h)			
Radioassay	15.3 (11.6, 18.4)	10.7 (9.4, 17.4)	13.6 (9.8, 17.9)
ELISA	6.8 (5.5, 15.6)	6.4 (4.2, 8.5)	6.8 (4.8, 10.7)
Secondary outcomes			
Systemic 11β-HSD1 activity			
Urinary (THF+alloTHF)/THE ratio	1.0 (0.8, 1.2)	1.0 (0.8, 1.1)	1.0 (0.8, 1.1)
Cortisol (µg/24 h)	68.5 (40.0, 101.0)	75.5 (48.0, 84.0)	74.5 (47.5, 94.0)
Epidermal barrier TEWL (g/h/m^2^)*	2.1 ± 0.4 (8.2)	2.2 ± 0.3 (9.1)^†^	2.2 ± 0.4 (8.6)^‡^
Epidermal integrity (tape strips)*	3.8 ± 0.4 (44.5)	3.9 ± 0.5 (50.5)^†^	3.9 ± 0.4 (47.5)^‡^
Epidermal thickness (μm)	62.8 (54.5, 69.2)	65.7 (60.7, 69.3)	63.8 (58.4, 69.3)
Skin hydration (AU)	40.5 (34.5, 46.2)	40.4 (36.7, 45.5)	40.5 (36.1, 45.8)
Sudomotor nerve function (μS)			
Left hand	54.0 (47.0, 63.0)	57.5 (39.0, 71.0)	56.0 (46.5, 68.5)
Right hand	54.0 (47.0, 60.0)	56.5 (41.0, 67.0)	56.0 (45.5, 61.5)
Hands	53.8 (48.0, 60.5)	56.8 (40.0, 70.5)	56.8 (47.3, 65.0)
Left foot	65.5 (46.0, 78.0)	79.0 (63.0, 84.0)	74.5 (57.0, 81.5)
Right foot	69.5 (48.0, 79.0)	77.0 (69.0, 82.0)	74.0 (54.5, 79.0)
Feet	67.5 (44.5, 76.5)	78.0 (64.0, 83.5)	75.0 (55.8, 79.8)
Overall	61.0 (46.8, 67.8)	66.6 (63.3, 71.3)	64.6 (50.3, 69.8)
Safety variables			
BMI (kg/m^2^)	33.67 ± 13.47	35.05 ± 5.68	34.36± 10.17
Waist–hip ratio	0.98 ± 0.08	1.03 ± 0.08	1.01 ± 0.08
HbA1c (mmol/mol)	72.29 ± 19.43	66.00 ± 14.91	69.14 ± 17.29
Blood pressure (mmHg)			
Systolic	135.71 ± 21.01	140.43 ± 12.02	138.07 ± 16.97
Diastolic	83.86 ± 8.91	72.64 ± 9.96	78.25 ± 10.89
Full blood count			
Hemoglobin (g/L)	139.21 ± 14.12	139.43 ± 11.35	139.32 ± 12.57
White blood cells (×10^9^/L)	6.46 ± 2.55	7.34 ± 2.04	6.90 ± 2.31
Platelets (×10^9^/L)	218.71 ± 61.85	273.14 ± 81.37	245.93 ± 76.15
Red blood cells (×10^12^/L)	4.84 ± 0.49	4.71 ± 0.48	4.78 ± 0.48
Mean corpuscular volume (fL)	88.43 ± 4.64	91.21 ± 6.96	89.82 ± 5.98
Hematocrit (packed cell volume)	0.43 ± 0.04	0.43 ± 0.03	0.43 ± 0.03
Mean corpuscular hemoglobin (pg)	28.82 ± 2.09	29.74 ± 2.47	29.28 ± 2.29
Corpuscular hemoglobin (g/L)	325.57 ± 10.43	326.64 ± 8.29	326.11 ± 9.26
Red blood cell distribution width (%)	13.88 ± 0.98	14.12 ± 1.10	14.00 ± 1.03
Lipid profile			
High density lipoprotein (mmol/L)	1.24 ± 0.31	1.19 ± 0.27	1.21 ± 0.29
Cholesterol (mmol/L)	4.36 ± 1.13	3.95 ± 0.75	4.15 ± 0.97
Triglycerides (mmol/L)	1.68 ± 0.74	2.02 ± 1.02	1.85 ± 0.89
Liver function			
Albumin (g/L)	39.43 ± 2.56	39.50 ± 2.44	39.46 ± 2.46
Bilirubin (µmol/L)	8.93 ± 2.97	8.14 ± 2.82	8.54 ± 2.87
Alkaline phosphatase (U/L)	80.43 ± 23.76	85.07± 26.08	82.75 ± 24.59
Alanine aminotransferase (IU/L)	24.64 ± 7.10	27.43± 10.91	26.04± 9.14
Aspartate aminotransferase (IU/L)	21.00 ± 4.95	22.64± 5.50	21.82 ± 5.20
Gamma-glutamyl transpeptidase (IU/L)	41.86 ± 33.87	39.29 ± 29.87	40.57 ± 31.37
Kidney functioL			
eGFR (mL/min/1.73 m^2^)	81.86 ± 9.69	79.21 ± 13.59	80.54 ± 11.66
Sodium (mmol/L)	138.93 ± 2.06	140.71 ± 5.47	139.82 ± 4.15
Potassium (mmol/L)	4.46 ± 0.33	4.63 ± 0.41	4.55 ± 0.37
Urea (mmol/L)	6.96 ± 2.81	7.25 ± 2.17	7.10 ± 2.47
Creatinine (µmol/L)	76.64 ± 15.36	76.21 ± 20.04	76.43 ± 17.52
Adrenal function			
Testosterone (nmol/L)	11.10 ± 6.25	6.97± 5.48	9.04± 6.14
DHEAS (µmol/L)	3.53 ± 2.19	2.91± 1.69	3.22 ± 1.95
Thyroid function			
Free thyroxine (pmol/L)	14.86 ± 2.20	15.73 ± 1.58	15.30 ± 1.93
Thyroid stimulating hormone (mlU/L)	1.74 ± 0.66	1.72 ± 0.63	1.73 ± 0.64

*values are log mean ± s.d. and values in parentheses is the geometric mean; ^†^ *n*  = 13;^ ‡^ *n*  = 27.

*n*, number non-missing; Q1, first quartile; Q3, third quartile.

### Efficacy

#### Primary outcome skin 11β-HSD1 activity

**Figure 2:**
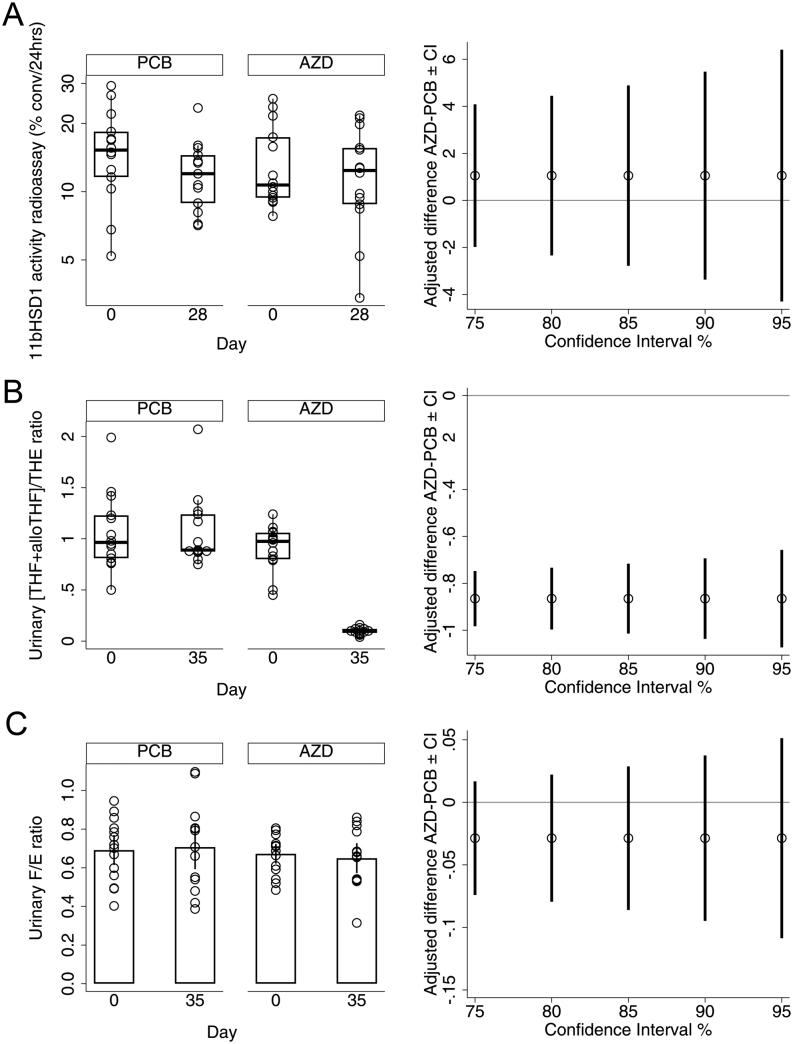
Efficacy outcome measures. Population: Full analysis set. (A) Box plots of observed skin 11β-HSD1 activity (percent conversion (conv) per 24 h measured by radioassay) (left panel) and adjusted differences between placebo (PCB) and AZD4017 (AZD) medians at day 28 with CIs estimated in imputed data (right panel). (B) Box plots of observed urinary (THF+alloTHF)/THE ratio (left panel), indicative of systemic 11β-HSD1 activity and adjusted differences between medians at day 35 with CIs estimated in imputed data (right panel). (C) Means and 90% CIs for observed urinary cortisol/cortisone (F/E) ratio (left panel), indicative of systemic 11β-HSD2 activity and adjusted differences between means at day 35 with CIs estimated in observed data (right panel). Solid lines indicate no difference.

Median 11β-HSD1 activity (% conversion per 24 h) by radioassay at day 28 was similar in both treatments after adjustment for baseline activity, baseline HbA1c, age, and sex. The median was 11.8% with placebo and 12.8% with AZD4017, with a difference of 1.1% (−3.4, 5.5, Fig. 2A, and Table 2). This outcome was unaffected in sensitivity analysis after the removal of two baseline samples that were considered unreliable.

**Table 2:** Primary and secondary efficacy outcomes; adjusted differences. Population: Full analysis set. Multiple imputation was used to address missing data. For TEWL, integrity, wound depth, and diameter, linear regression was used to estimate CIs around differences between the groups. TEWL and integrity measurements were log-transformed before analysis; differences are expressed as ratios of geometric means between groups (AZD4017:placebo). For the remaining variables, which did not meet assumptions for linear regression, median regression was used. For each variable, the comparison was adjusted for the variable’s baseline value (this was not available for wound diameter and depth), age, sex, and baseline HbA1c. All TEWL readings were adjusted using pre-disruption TEWL at baseline. Between-group differences were not calculated for biopsy and plasma AZD4017 because available placebo values did not vary, and all were below the detection limit.

Variable	Observed				Estimated median*		Difference*	90% CI
	Placebo		AZD4017		Placebo, *n* = 14	AZD4017, *n* = 14		
Median (Q1, Q3)	*n*	Median (Q1, Q3)	*n*					
Primary outcome								
Skin 11β-HSD1 activity (% conversion/24 h): day 28								
Radioassay	12.0 (8.9, 14.5)	13	12.7 (8.8, 15.6)	14	11.8	12.8	1.1	(−3.4, 5.5)
ELISA	5.6 (1.0, 7.6)	13	4.3 (3.1, 5.0)	14	4.9	4.3	−0.5	(−3.8, 2.7)
Secondary outcomes								
Systemic 11β-HSD1 activity: day 35								
Urinary (HF+alloTHF)/THE ratio	0.89 (0.88, 1.24)	13	0.10 (0.08, 0.12)	13	1.00	0.13	−0.87	(−1.04, −0.69)
Cortisol (µg/24h)	55.0 (47.0, 120.0)	13	62.0 (52.0, 73.0)	13	61.6	66.9	5.3	(−30.2, 40.7)
Epidermal thickness (μm)	57.5 (55.7, 67.3)	13	66.3 (60.6, 74.4)	12	61.2	66.8	5.6	(−4.9, 16.0)
Skin hydration (A.U)	40.5 (29.4, 46.0)	12	45.3 (37.0, 46.5)	12	38.0	43.7	5.7	(−2.8, 14.1)
Sudomotor (nerve) function (μS): day 35								
Left hand	58.0 (48.0, 63.0)	13	64.0 (59.0, 72.0)	13	55.3	65.7	10.4	(−1.8, 22.6)
Right hand	55.0 (51.0, 60.0)	13	63.0 (57.0, 69.0)	13	55.6	60.6	5.0	(−6.7, 16.8)
Hands	57.5 (49.5, 61.0)	13	63.5 (59.0, 70.5)	13	55.8	63.0	7.3	(−4.8, 19.3)
Left foot	70.0 (61.0, 76.0)	13	80.0 (64.0, 82.0)		75.5	68.5	−7.0	(−18.4, 4.3)
Right foot	65.0 (63.0, 75.0)	13	80.0 (68.0, 82.0)	13	73.0	68.9	−4.1	(−13.2, 5.1)
Feet	68.5 (62.0, 75.0)	13	80.0 (66.0, 82.5)	13	73.8	69.3	−4.5	(−14.5, 5.4)
Overall	59.8 (53.8, 68.0)	13	70.8 (64.5, 73.0)	13	65.0	63.7	−1.3	(−9.2, 6.6)
AZD4017 (nmol/L)								
Biopsy: day 28	<5 (<5, <5)	13	1570 (876, 2440)	14				
Plasma: day 35	<5 (<5, <5)	7	6490 (2960, 9040)	12				
Wound healing (mm) after day 0 biopsies								
Diameter: day 2	1.49 (0.72)	14	0.98 (0.70)	14	1.51	0.98	−0.52	(−0.95, −0.10)
Depth: day 7	0.60 (0.23)	14	0.59 (0.16)	14	0.60	0.59	−0.01	(−0.15, 0.13)
Wound healing after day 28 biopsies								
Diameter: day 30	1.44 (0.70)	11	0.65 (0.49)	12	1.35	0.70	−0.65	(−1.15, 0.16)
Depth: day 35	0.60 (0.17)	13	0.54 (0.21)	12	0.59	0.56	−0.03	(−0.16, 0.09)
Epidermal barrier TEWL (g/h/m^2^): day 35	1.9 (0.4); 7.0	13	2.2 (0.5); 8.9	12	6.9	9.2	1.33	(0.97, 1.82)
Epidermal integrity (tape strips): day 28	3.6 (0.6); 37.9	13	4.0 (0.4); 55.6	14	39.0	55.8	1.43	(1.07, 1.91)
Epidermal barrier recovery TEWL (g/h/m^2^) after day 0 disruption								
Hour 3	3.6 (0.3); 34.9	14	3.4 (0.2); 30.8	12	35.1	32.0	0.91	(0.76, 1.09)
Hour 48	3.0 (0.3); 19.8	14	3.1 (0.4); 21.3	11	20.4	20.6	1.01	(0.79, 1.28)
Hour 168	2.6 (0.3); 13.5	13	2.8 (0.7); 16.1	13	14.1	15.6	1.11	(0.76, 1.62)
Epidermal barrier recovery TEWL (g/h/m^2^) after day 28 disruption								
Hour 3	3.2 (0.5); 23.8	13	3.4 (0.3); 29.8	14	23.0	30.7	1.33	(0.99, 1.79)
Hour 48	2.7 (0.4); 14.4	13	2.9 (0.4); 18.2	14	14.6	18.4	1.26	(0.94, 1.67)
Hour 168	2.3 (0.4); 10.0	13	2.4 (0.4); 10.8	12	10.1	10.8	1.07	(0.79, 1.44)

*Estimated using multiple imputation, adjusted for baseline value (if applicable), age, sex, and baseline HbA1c.

Another sensitivity analysis that used an alternative ELISA method had an acceptable correlation with the radioassay method at baseline (Spearman’s rho = 0.70) but not at day 28 (Spearman’s rho = 0.19) (Table 3 and Supplementary Fig. 1). ELISA results were consistent with the radioassay method. Therefore, the pilot data offered no proof-of-concept of 11β-HSD1 inhibition by AZD4017 in skin, in contrast to the systemic effects detaled below.

**Table 3:** Longitudinal laboratory safety variables; adjusted differences. Population: safety set. All point estimates and CIs were estimated using linear regression in imputed data (*n* = 14 for placebo, *n*  = 14 for AZD4017).

Variable	Difference* AZD4017-placebo (90% CI)			
	Day 7	Day 28	Day 35	Day 42
BMI (kg/m^2^)	N/A	N/A	0.41 (−0.51, 1.34)	N/A
Waist–hip ratio	N/A	N/A	0.02 (−0.01, 0.05)	N/A
HbA1c (mmol/mol)	0.18 (1.51, 1.88)	2.17 (0.64, 4.99)	0.72 (−3.64, 5.09)	2.42 (−2.38, 7.22)
Blood pressure (mmHg)				
Systolic	N/A	N/A	−14.74 (−23.00, −6.47)	0.77 (−6.58, 8.11)
Diastolic	N/A	N/A	−2.43 (−7.67, 2.80)	2.09 (−6.77, 10.95)
Full blood count				
Hemoglobin (g/L)	0.67 (−2.12, 3.45)	−0.98 (−4.13, 2.17)	0.64 (−3.96, 5.24)	2.07 (−1.62, 5.76)
White blood cells (×10^9^/L)	0.54 (−0.14, 1.22)	−0.40 (−0.87, 0.08)	−0.31 (−0.96, 0.34)	0.33 (−0.41, 1.07)
Platelets (×10^9^/L)	2.42 (−13.65, 18.50)	13.32 (−9.56, 36.20)	−0.91 (−25.20, 23.37)	1.47 (−16.74, 19.68)
Red blood cells (×10^12^/L)	−0.05 (−0.17, 0.06)	−0.05 (−0.15, 0.04)	−0.02 (−0.15, 0.12)	0.15 (0.03, 0.26)
Mean corpuscular volume (fL;)	0.11 (−1.83, 2.05)	0.52 (−1.49, 2.53)	−0.17 (−2.53, 2.19)	0.16 (−1.42, 1.73)
Hematocrit (packed cell volume)	−0.01 (−0.02, 0.01)	0.00 (−0.01, 0.02)	0.00 (−0.02, 0.02)	0.02 (0.00, 0.03)
Mean corpuscular hemoglobin (pg)	0.41 (0.08, 0.75)	−0.07 (−0.44, 0.31)	0.17 (−0.28, 0.62)	−0.42 (−0.95, 0.10)
Corpuscular hemoglobin (g/L)	4.91 (−1.41, 11.22)	−3.80 (−10.80, 3.21)	1.75 (−4.85, 8.35)	−5.22 (−12.18, 1.74)
Red blood cell distribution width (%)	−0.36 (−0.89, 0.18)	−0.09 (−0.49, 0.31)	0.11 (−0.28, 0.51)	−0.21 (−0.68, 0.26)
Lipid profile				
High-density lipoprotein (mmol/L)	−0.06 (−0.14, 0.02)	−0.08 (−0.16, 0.00)	−0.13 (−0.21, −0.05)	0.05 (−0.07, 0.16)
Cholesterol (mmol/L)	−0.42 (−0.60, −0.23)	−0.46 (−0.79, −0.12)	−0.51 (−0.87, −0.15)	−0.10 (−0.54, 0.34)
Triglycerides (mmol/L)	−0.14 (−0.67, 0.40)	−0.19 (−0.56, 0.18)	−0.48 (−1.21, 0.24)	0.02 (−0.51, 0.55)
Liver function				
Albumin (g/L)	0.37 (−0.82, 1.55)	−0.37 (−1.82, 1.09)	−0.01 (−1.60, 1.59)	0.40 (−0.59, 1.38)
Bilirubin (µmol/L)	0.93 (−1.14, 3.00)	0.60 (−0.89, 2.09)	−0.64 (−3.01, 1.73)	0.35 (−1.17, 1.87)
Alkaline phosphatase (U/L)	−6.00 (−12.44, 0.44)	−12.39 (−20.21, −4.56)	−13.09 (−20.43, −5.75)	−5.65 (−15.25, 3.95)
Alanine aminotransferase (IU/L)	1.16 (−1.84, 4.15)	−1.96 (−4.94, 1.03)	−1.64 (−5.88, 2.61)	0.78 (−3.00, 4.56)
Aspartate aminotransferase (IU/L)	0.46 (−2.29, 3.21)	0.12 (−1.81, 2.05)	−0.04 (−2.52, 2.45)	2.21 (−1.02, 5.44)
Gamma-glutamyl transpeptidase (IU/L)	0.76 (−2.83, 4.35)	−10.28 (−14.96, −5.60)	−11.97 (−22.80, −1.15)	−9.55 (−14.88, −4.22)
Kidney function				
eGFR (mL/min/1.73 m^2^)	−3.18 (−8.30, 1.94)	−2.19 (−5.38, 1.01)	0.27 (−4.57, 5.10)	1.62 (−2.77, 6.01)
Sodium (mmol/L)	0.10 (−1.23, 1.44)	0.16 (−0.98, 1.29)	−1.19 (−3.21, 0.82)	−1.25 (−2.42, −0.07)
Potassium (mmol/L)	−0.31 (−0.58, −0.03)	0.18 (−0.06, 0.43)	0.01 (−0.22, 0.25)	−0.04 (−0.31, 0.22)
Urea (mmol/L)	0.13 (−0.79, 1.05)	−0.35 (−1.31, 0.61)	−0.52 (−1.88, 0.83)	0.02 (−1.04, 1.08)
Creatinine (µmol/L)	7.43 (0.79, 14.08)	3.43 (−0.98, 7.85)	1.78 (−5.65, 9.22)	−1.58 (−8.24, 5.07)
Adrenal function				
Testosterone (nmol/L)	−0.63 (−2.52, 1.25)	−1.06 (−2.58, 0.45)	−0.40 (−2.66, 1.85)	1.12 (−1.20, 3.45)
DHEAS (µmol/L)	2.30 (1.52, 3.08)	2.86 (2.09, 3.64)	2.16 (1.29, 3.04)	1.09 (0.43, 1.75)
Thyroid function				
Free thyroxine (pmol/L)	0.50 (−0.63, 1.64)	0.51 (−0.43, 1.44)	0.65 (−0.45, 1.76)	0.79 (0.07, 1.52)
Thyroid stimulating hormone (mlU/L)	0.10 (−0.22, 0.42)	−0.20 (−0.58, 0.19)	−0.15 (−0.54, 0.23)	−0.04 (−0.46, 0.38)

*Estimated in imputed data, adjusted for baseline value, age, sex, and baseline HbA1c.

#### Systemic 11β-HSD1 activity

Systemic 11β-HSD1 activity was inferred from 24 h urinary steroid metabolite ratios tetrahydrocortisol + 5α-tetrahydrocortisol/tetrahydrocortisone ((THF+alloTHF)/THE) by liquid chromatography-tandem mass spectrometry (23). In contrast to the skin, systemic 11β-HSD1 activity was lower with AZD4017; the adjusted median was 1.00 with placebo and 0.13 with AZD4017, and the difference was −0.87 (−1.04, −0.69) (Fig. 2B and Table 2). All supplementary CIs up to 95% excluded 0, suggesting that median systemic 11β-HSD1 activity may be at least 69% (median 87%) lower in participants taking AZD4017. As anticipated, the results showed no evidence that urinary cortisol/cortisone ratio (a measure of systemic 11β-HSD2 activity that deactivates cortisol to cortisone; unplanned analysis) was affected by AZD4017. The adjusted median was 0.71 with placebo and 0.65 with AZD4017; the difference was −0.06 (−0.19, 0.07, Fig. 2C).

#### Wound healing

**Figure 3:**
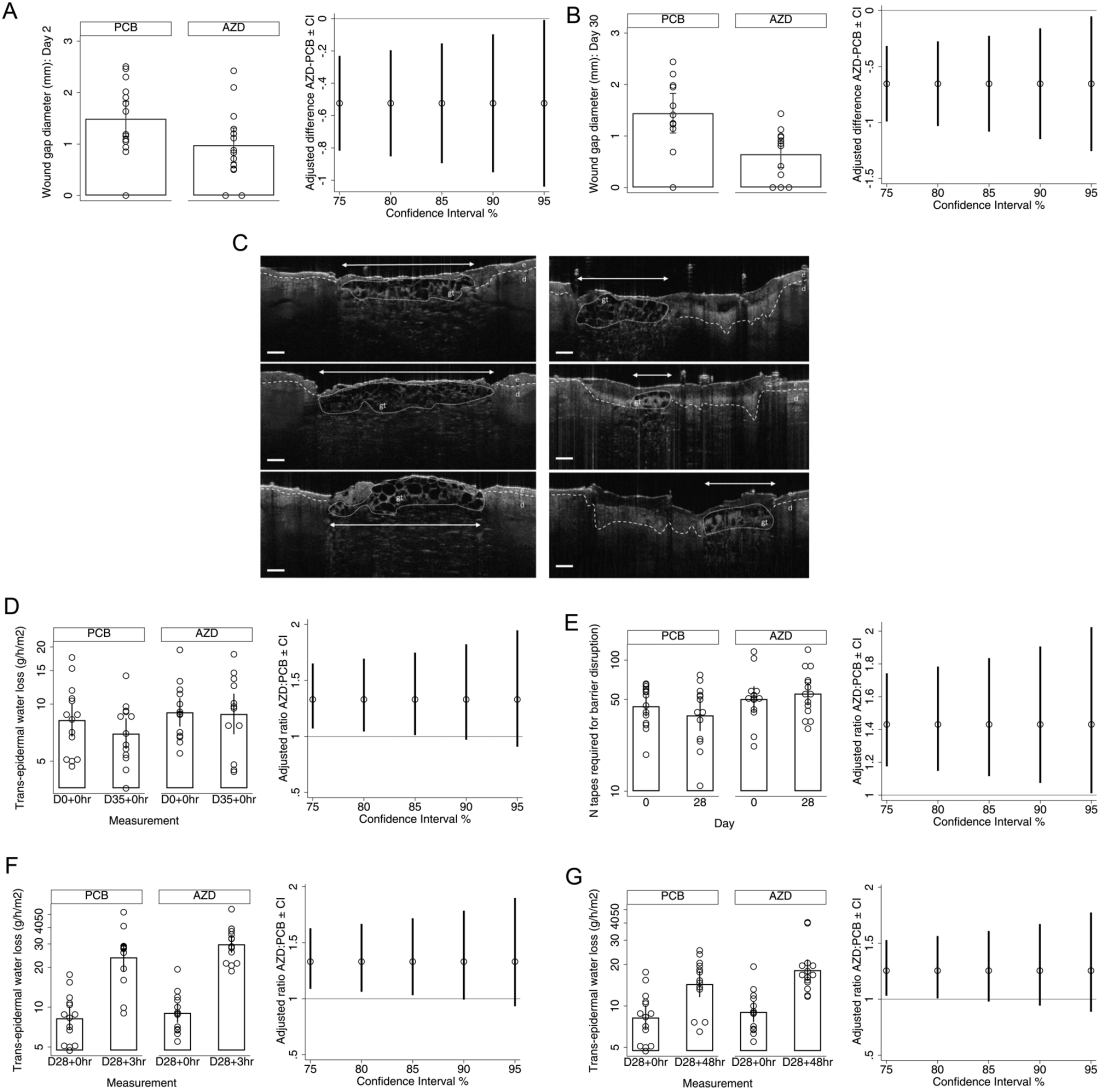
Skin outcome measures. Population: Full analysis set. (A, B, D, E, F, and G) Means and 90% CIs for observed values (left panel) and adjusted differences between PCB and AZD means with CIs estimated in imputed data (right panel). TEWL and integrity readings were log-transformed before analysis; therefore, geometric means and ratios are provided for these variables. Solid lines indicate no difference. (C) Representative day 2 biopsy wound healing optical coherence tomography images (after 2 days of treatment). Maximal early granulation tissue width (arrow) was measured for participants treated with PCB (left panel) or AZD4017 (right panel). Wide dashes indicate dermal–epidermal junction. Scale bar: 250 µm. AZD, AZD4017; d, dermis; e, epidermis; gt, granulation tissue; PCB, placebo; TEWL, transepidermal water loss.

Based on maximal granulation tissue width (a marker of early wound healing), the mean wound gap 2 days after each of the separate biopsies was lower with AZD4017 at all levels of confidence. The mean difference was −0.52 mm (−0.95 mm, −0.10 mm) at day 2 and −0.65 mm (−1.15 mm, −0.016 mm) at day 30, corresponding to a 7–63% (mean 34%) and 12–85% (mean 48%) narrower wound gap diameter, respectively, with AZD4017 (Fig. 3A, B and Table 2). Representative wound healing images for participants treated with placebo and AZD4017 are presented in Fig. 3C.

Based on maximal clot depth (a marker of late wound healing), the data showed no indication of a difference on either day 7 or day 35 (Table 2).

#### Epidermal barrier function

The epidermal barrier is essential in guarding against water loss and infection. Barrier defects are associated with a range of skin pathologies, for example, atopic dermatitis (24), and functions are regulated by GC in a complex manner (25), but the role of 11β-HSD1 is unknown.

TEWL, the gold-standard measure of epidermal barrier function, was on average 33% higher with AZD4017 on day 35 (Fig. 3D and Table 2), but 90% CI included 0. At lower levels of confidence, resting TEWL was higher with AZD4017, and in sensitivity analysis (which excluded potentially unreliable values), the 90% CI was 3–88%.

#### Epidermal integrity

Epidermal integrity contributes to skin resilience against mechanical damage (wounding), measured by resistance to sequential tape stripping of epidermal layers.

The number of tapes required to achieve the same degree of barrier disruption (TEWL of 40–50 g/h/m^2^) was 7–91% (mean 43%) higher with AZD4017 (Fig. 3E and Table 2), equivalent to an additional 16.8 tapes.

#### Epidermal barrier recovery

The first set of post-disruption recovery measurements, collected between baseline and day 7, provided no proof of concept for a difference in TEWL (Table 2).

The second set of post-disruption recovery measurements, collected between days 28 and 35, indicated higher TEWL with AZD4017 at 3 h and 48 h, at 80–85% confidence, although the CIs were wide (Fig. 3F, G and Table 2). By 168 h after disruption, there was no difference (Table 2).

Together with pre-disruption epidermal barrier findings, these preliminary results suggest a novel role for skin 11β-HSD1 in the maintenance of epidermal barrier homeostasis, which would require confirmation in larger future trials.

#### Epidermal thickness

Although the observed median epidermal thickness was greater with AZD4017 at day 35, median skin thickness could be between 8% thinner and 26% thicker with AZD4017 (Table 2).

#### Skin hydration

Dry skin is less resistant to wounding and is common in people with diabetes (26).

Although the observed median hydration was higher with AZD4017, consistent with proof of concept, hydration could be between 7% lower and 37% higher in those receiving AZD4017 (Table 2).

#### Sudomotor nerve function

Reduced sudomotor nerve function is a leading cause of chronic foot ulcers (27) but regulation by GC is unknown.

Averaged across all sites, there was no proof of concept of a difference in sudomotor function; results suggested function could be between 14% worse and 10% better with AZD4017 (Table 2). There was also no proof of concept (and CIs included only modest potential differences in either direction) when results were averaged for hands and feet separately.

Sensitivity analyses indicated worse foot sudomotor function with AZD4017 at 75% confidence, but all other results from this additional analysis were consistent with the main findings.

### Compliance

Mean diary card compliance was >97.9% in both arms at all visits and >99.6% at day 35. Mean treatment compliance at each visit was >95.2% in both arms and >97.9% at day 35; overall treatment compliance was 84–101% with placebo and 93–101% with AZD4017. Drug exposure data in plasma and skin did not indicate any discrepancies between reported compliance rates and actual exposure, which suggests that participants had correctly adhered to their assigned treatment regimen.

As anticipated, AZD4017 levels in day 28 skin biopsies and day 35 plasma samples correlated moderately (Spearman’s rho = 0.54; Supplementary Fig. 2), further validating drug tissue penetration.

### Correlations between compliance and efficacy outcomes

In those taking AZD4017, several outcome measures displayed a possible association with compliance (absolute rho >0.3), including negative associations with 11β-HSD1 activity (skin activity measured by ELISA and systemic activity measured by urinary (THF+alloTHF)/THE ratios) and positive associations with TEWL and skin hydration.

### Safety

#### Biopsy and ECG

Biopsy findings did not raise any clinical concerns, and all passed a physical inspection. No incidents of infection were observed. Participants did not report any significant pain or discomfort from the biopsies, which all healed well.

No participants remaining in the trial at day 42 (13 with placebo and 14 with AZD4017) showed clinically meaningful ECG anomalies.

#### Longitudinal laboratory safety data

**Figure 4:**
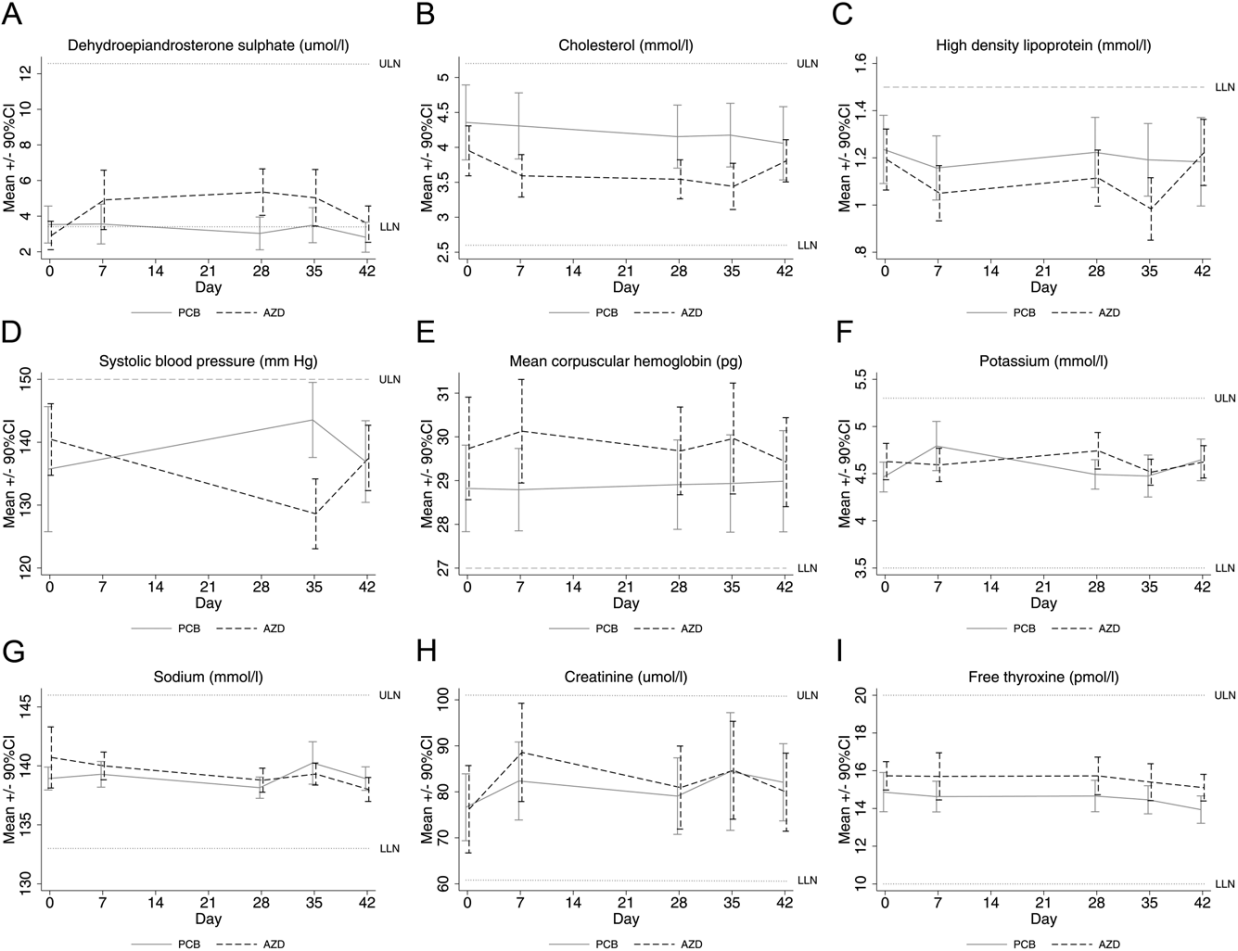
Longitudinal laboratory safety data. Population: safety set. Means and 90% CIs with PCB and AZD over time in cases with data available. AZD, AZD4017; LLN, lower limit of normal (where applicable); PCB, placebo; ULN, upper limit of normal.

With AZD4017 (Table 3) DHEAS was higher on days 7, 28, 35, and 42; total cholesterol was lower on days 7, 28, and 35; high-density lipoprotein was lower on day 35, and systolic blood pressure was lower on day 28 (Fig. 4A, B, C, D and Table 3).

Apart from some small differences which were not considered to be clinically relevant, the remaining variables did not differ (Fig. 4E, F, G, H, I and Table 3).

Although several laboratory findings (with both treatments) were above or below accepted clinically normal limits, few warranted further investigation or intervention by the study team, and none resulted in treatment withdrawal.

#### Adverse events

Thirty-seven individual adverse events (AEs) occurred, of which 13 were with placebo and 24 with AZD4017 (Table 4). When recurring instances of the same AE within a participant were counted as a single AE, 29 unique AEs occurred, of which 16 were with AZD4017 and 13 with placebo. Most AEs involved the gastrointestinal, nervous, or respiratory systems. Twenty-six AEs were mild and three were moderate. None of the 29 unique AEs were considered probably or definitely related to the study drug, 20 were possibly related, and 8 were unlikely to be related. AEs were broadly balanced across treatment arms, although all three moderately severe events occurred with AZD4017.

**Table 4:** Adverse event summary. Population: safety set.

	PCB (*n* = 14)	AZD (*n* = 14)	All participants (*n* = 28)
AEs			
Total AEs, *n* (*n* per PY)	13 (8.3)	24 (13.8)	37 (11.1)
Unique AE, *n* (*n* per PY)	13 (8.3)	16 (9.2)	29 (8.7)
Patients with ≥1 AE, *n* (%)	8 (57)	10 (71)	18 (64)
Discontinuation due to AE, *n* (%)	0 (–)	0 (–)	0 (–)
AEs by SOC: total *n* (unique n*), *n* (%)			
Gastrointestinal	9 (9), 6 (43)	14 (8), 7 (50)	23 (17), 13 (46)
Infections	0 (0), 0 (–)	1 (1), 1 (7)	1 (1), 1 (4)
Musculoskeletal	0 (0), 0 (–)	1 (1), 1 (7)	1 (1), 1 (4)
Nervous system	1 (1), 1 (7)	6 (4), 4 (29)	7 (5), 5 (18)
Respiratory	2 (2), 1 (7)	2 (2), 1 (7)	4 (4), 2 (7)
Skin	1 (1), 1 (7)	0 (0), 0 (–)	1 (1), 1 (4)
AE severity, n*			
Mild	13	13	26
Moderate	0	3	3
AEs by relation to study drug, n*			
Not related	0	1	1
Unlikely	4	4	8
Possible	9	11	20
SAEs			
Total SAE, *n*	0	1	1
Patients with >1 SAE, *n* (%)	0 (–)	1 (7)	1 (4)

*Recurrent AEs counted once at maximum severity and relatedness reported.

AE, adverse event; PY, patient year; SAE, serious adverse events; SOC, Common Terminology Criteria for Adverse Events system order class.

### Future study power estimation

Based on power calculations from the current trial, we anticipate that 100–150 participants per arm should suffice to detect a difference of 20% or more in comparison with placebo for all outcome measures.

### Feasibility

Our assessment of the study’s recruitment rate, proportions meeting each eligibility criterion, the proportion of eligible candidates consenting to take part, and data completeness found that a future confirmatory trial is feasible.

## Discussion

Our double-blind, randomized, placebo-controlled, parallel-group pilot clinical trial provides preliminary evidence that wounds were smaller and skin integrity was greater in people with type 2 diabetes with systemic 11β-HSD1 inhibition. This finding potentially represents a major advance in the development of 11β-HSD1 inhibitors as novel therapies for diabetic ulcers. Nevertheless, all findings from this pilot study are considered exploratory, which requires confirmation in an adequately powered phase 2 trial.

Studies previously demonstrated effective 11β-HSD1 inhibition in mouse skin (13, 16), but this had not been explored in humans. In this pilot study, data on our primary outcome measure failed to show 11β-HSD1 inhibition in the skin. The absence of skin 11β-HSD1 inhibition was not due to lack of exposure *in situ* because AZD4017 levels in skin correlated with plasma levels, albeit at lower concentrations. Alternatively, ineffective AZD4017 efficacy *ex vivo* (low target affinity) or insufficient assay sensitivity (as baseline 11β-HSD1 activity was relatively low) may explain this finding. In this situation, the analysis plan stipulated that differences in secondary outcomes were only to be interpreted if AZD4017 inhibited systemic 11β-HSD1 activity. Indeed, we found AZD4017 reduced [THF+alloTHF]/THE ratios and elevated DHEAS. This finding is consistent with that of other selective 11β-HSD1 inhibitor trials, including AZD4017 (28, 29, 30, 31, 32). Importantly, 11β-HSD2 activity was unaffected by AZD4017 (23, 33).

Our secondary outcomes, although exploratory, offer a series of novel insights into the potential effects of 11β-HSD1 inhibition on skin function. Optical coherence tomography is a validated method for the non-invasive assessment of wound healing (34), and this is the first application in a randomized controlled trial. Our finding of improved early granulation tissue healing in day 2 wounds (across all CIs) with only 2 days of AZD4017 treatment is very promising, especially as overall healing in this cohort was normal, and participants’ diabetes was well managed. Further, this effect was greater after 30 days of treatment, suggesting improved efficacy with increased AZD4017 exposure.

By day 7 after wounding, early granulation tissue remodeling was complete in all participants and no effect with AZD4017 was detected, potentially because we measured clot depth, rather than volume. In a subsequent study, we developed a new machine learning method for automated volumetric quantification of key morphological features of wound healing using the optical coherence tomography scans from this clinical trial, which further supports our finding that AZD4017 promotes wound healing (35). These novel pilot human data are consistent with the known deleterious effects of GC on wound healing (36) and with preclinical evidence of improved healing by 11β-HSD1 inhibition in animal models of stress, obesity, GC excess, and aging from our group and others (13, 16, 17, 18).

We also found that AZD4017 increased epidermal integrity, an improvement compared to the greater epidermal integrity of young vs aged skin (37). This is supported by studies demonstrating reduced skin integrity after human psychological stress (38) or exogenous GC treatment (39) and strengthens the basis for further development of AZD4017 to improve skin function.

Provisional evidence of modest impairments in epidermal barrier function and recovery with AZD4017 was also observed in our study. GCs are known to both impair and promote epidermal barrier function, the former during exogenous GC treatment (39, 40, 41) and the latter during endogenous GC excess (25). Our results are in agreement, showing for the first time that endogenous cortisol may promote epidermal barrier function via 11β-HSD1.

GCs impair healing through multiple mechanisms, for example, by inhibiting keratinocyte re-epithelialization whilst promoting keratinocyte differentiation (essential for barrier function) (9), supporting our findings of improved wound healing and impaired barrier function by AZD4017. Future 11β-HSD1 inhibitor trials should monitor the epidermal barrier, particularly in participants with compromised function (e.g. atopic dermatitis). Other key mechanisms of wound healing defect in aging and diabetes include progenitor (stem) cell recruitment and angiogenesis (42), both associated with GC excess (43, 44). Abnormal immune cell function is also implicated in chronic wound etiology, for example, increased neutrophil extracellular trap formation (45) but the role of GC in this process is unknown. Such detailed mechanistic studies were beyond the scope of this pilot trial and regulation of these pathways by 11β-HSD1 remains an important area for future research.

Peripheral neuropathy is a serious complication in diabetes but regulation by GC is unknown. Nerve function was determined by sudomotor analysis, as previously described (27). In our study, AZD4017 did not affect nerve function overall or in the hands. At the lowest level of confidence, a modest impairment occurred in the feet. Therefore, future trials should also monitor nerve function to rule out any adverse effects.

We found no significant safety concerns in this small pilot study. Indeed, we found improvements in cardiovascular risk factors (notably total cholesterol and systolic blood pressure) in the AZD4017 arm. These improvements are consistent with results from other 11β-HSD1 inhibitor trials (28, 30, 46, 47). Cardiovascular risk factors are a strong predictor of ulcers, healing, complications, and recurrence (48, 49). Therefore, oral 11β-HSD1 inhibitors could also reduce ulcer recurrence but this requires further studies with long-term 11β-HSD1 inhibitor treatment. Concomitant medication use was not formally measured in this trial but warrants monitoring in these future studies.

Our study has several limitations, including the use of biopsy healing rather than ulcers, which was necessary to ensure AZD4017 was safe in acute wounds and well-managed diabetes, before application to more severe disease. Importantly, however, our approach enabled the study of wound healing in a standardized manner, where issues such as infection and ulcer chronicity that would influence healing to apply to a lesser extent, thus enabling a more focused assessment of AZD4017 impact. Although our findings are promising, the small sample size and lack of inferential testing (consistent with recommendations for pilot trials) necessitate confirmation in future trials.

Together, our findings provide justification for a powered phase 2 clinical trial in people with diabetic foot ulcers with an established ulcer healing measure as the primary outcome. This will likely be multicenter, coordinated with routine appointments, and with randomization stratified for variables known to affect 11β-HSD1 function and wound healing (e.g. ulcer size and duration at presentation). Although requiring confirmation, the exploratory results from this pilot trial relating to healing, skin integrity, and cardiovascular risk factors for ulcer recurrence hold promise for 11β-HSD1 as a novel therapeutic target in wound repair.

## Supplementary Material

Research Protocol: Leeds Institute of Rheumatic and Musculoskeletal Medicine

Supplementary S3 – Statistical Methods

Table S1: Full descriptive data for primary and secondary efficacy variables in the full analysis set

Figure S1. Correlation between the different methods of assessing 24-hour 11β-HSD1 activity (percent conversion per 24 hours) in the full analysis set

## Declaration of interest

A A T reports grants, personal fees, and travel support from Sanofi, grants, personal fees, and educational events grants from Novo Nordisk, travel support from Merck Sharp and Dohme, personal fees and travel support from Boehringer Ingelheim, personal fees from Lilly, AstraZeneca, Bristol-Myers Squibb, and Janssen, equipment and travel support from ResMed, equipment from Philips Resporinics, Impeto Medical, and ANSAR Medical Technologies, grants and non-financial support from Napp, and equipment and support staff from BHR Pharmaceuticals Ltd. AAT is currently an employee of Novo Nordisk. This work was performed before AAT becoming a Novo Nordisk employee and Novo Nordisk had no role in this study. Wiebke Arlt is the Editor in Chief of European Journal of Endocrinology. Wiebke Arlt was not involved in the review or editorial process for this paper, on which she is listed as an author. The other authors have declared that no conflict of interest exists.

## Funding

This work was supported by a Medical Research Council http://dx.doi.org/10.13039/501100000265 Confidence in Concept Award to AT (MC_PC_15046), NIHR Senior Investigator Award to P M S (NF-SI-0514-10090), NIHR Leeds Biomedical Research Centre and NIHR Leeds In Vitro Diagnostic Evidence Co-operative.

## Trial registration

Clinicaltrials.gov NCT03313297, www.isrctn.com ISRCTN74621291.

## Data availability statement

All data associated with this study are available in the main text or the Supplementary materials.

## Author contribution statement

R A: conceptualization, design, investigation, resources, supervision, writing – review and editing; E M A H: conceptualization, design, software, formal analysis, data curation, visualization, writing – review and editing; K S: design, investigation, writing – review and editing; F D G: conceptualization, investigation, writing – review and editing; A A: design, investigation, writing – review and editing; R J F: design, resources, supervision; L W: design, resources, supervision; L P: design, resources, supervision; A F: design, resources, supervision, writing – review and editing; A E T: design, investigation, writing – review and editing, W A: design, investigation, writing – review and editing; A W M: conceptualization, supervision, writing – review and editing; A A T: conceptualization, writing – review and editing; P M S: conceptualization, funding acquisition, writing – review and editing; D A R: design, investigation, supervision, writing – review and editing; A T: conceptualization, design, funding acquisition, investigation, project administration, supervision, validation, writing – original draft, writing – review and editing. All authors revised the article for critically important content and approved the final version to be published.
