# Supplementary material for: Oral 11β-HSD1 inhibitor AZD4017 improves wound healing and skin integrity in adults with type 2 diabetes mellitus: a pilot randomized controlled trial
Source: Eur J Endocrinol. 2022 Feb 3;186(4):441–55. doi: 10.1530/EJE-21-1197 (PMC8942338; doi:10.1530/EJE-21-1197)
Supplement: Supplementary S3 – Statistical Methods [file supplementary_material_2.pdf]

Supplementary S3 – Statistical Methods

1.1 Primary outcome analysis

For the primary variable (24-hour 11β-HSD1 activity in skin at day 28), unadjusted and adjusted summaries (adjusted for gender, age, baseline 11β-HSD1 activity, and baseline HbA1c) for final values and changes from baseline are presented. Between-group differences in final values and changes from baseline are presented with two-sided 90% CIs, supplemented with CIs ranging from 75% to 95% in 5% increments as prespecified in the statistical analysis plan (SAP). Adjusted differences favoring the intervention arm (i.e., if 11β-HSD1 activity is lower) were considered preliminary evidence of efficacy.

Because model residuals were not normally distributed and a suitable data transformation could not be found, quantile (median) regression was used to obtain adjusted summaries and CIs, as prespecified in the SAP.

The primary analysis was on an intention-to-treat basis, with all patients included, as randomized. Analysis was conducted in the full analysis set using multiple imputation by chained equations to address missing data. Five-nearest-neighbor predictive mean matching was used for all variables; for each outcome, the imputation model included the repeated observations of the outcome, treatment assignment, age, sex, baseline HbA1c, and overall IMP compliance. Variables that correlated with the outcome or the likelihood of missingness and variables that had fewer missing values at a given time point were also included. Twenty datasets were imputed; inspection of Monte Carlo errors indicated that 20 were sufficient. Estimates were combined according to Rubin's rules. Sensitivity analyses using available case and last observation carried forward were also performed. A planned sensitivity analysis in the per-protocol set was not performed (see Section 1.7 below).

1.2 Secondary outcome analysis

The secondary endpoints, systemic 11β-HSD1 activity and skin function, and continuous clinical laboratory safety variables were analyzed in the same ways as the primary endpoints. Adjusted summaries were obtained using a linear regression model that mirrored the analysis of covariance approach for wound healing and laboratory safety variables. For all other variables, visual inspection of linear regression model residuals indicated that they were not normally distributed. For TEWL and epidermal integrity, log-transformation was performed before linear regression. For the remaining outcomes, for which log-transformation did not render normally-distributed linear regression model residuals, quantile (median) regression was used.

Planned supplementary analyses used linear mixed modelling to allow the precise timing of measurements to be included as a covariate where relevant and to account for any differences in timings between groups. Likelihood ratio tests supported the inclusion of non-linear (quadratic) terms for change over time and supported allowance of changes over time to vary between patients.

The standalone SAP pre-specified that in the event that there was not proof-of-concept for the primary outcome, effects on secondary outcomes would be interpreted with reference to the measured effects on systemic, as opposed to skin, 11β-HSD1 activity. If both showed a potential difference between groups, this would be considered preliminary evidence of efficacy. However, without evidence of a substantive difference in 11β-HSD1 activity either systemically or in the skin, any apparent substantive differences in secondary outcomes were to be interpreted with caution.

1.3 Additional pre-planned analyses

Correlations between plasma AZD4017 concentration at day 35 and skin AZD4017 concentration at day 28 and between AZD4017 compliance and efficacy outcomes in the active treatment arm were estimated using Spearman rank correlation.

We measured the strengths of associations between skin 11β-HSD1 activity and skin outcome measures while controlling for systemic GC level using partial correlation after rank transformation. Correlation coefficients were transformed using Fisher's z transformation before averaging across multiple imputed datasets. For all correlation analyses, absolute correlation coefficients with a value of r(ho) greater than 0.3 were considered preliminary evidence of substantive association.

The numbers of patients with clinical laboratory values below, within, or above normal ranges before the intervention and at each post-intervention time point were tabulated for each test for the safety population by treatment group. The proportions of patients who passed the overall assessment of blood safety at days 0, 7, 28, 35, 42 were summarized.

For AEs, summaries of incidence rates (frequencies and percentages), intensity, and relationship to study drug of individual AEs by system organ class and preferred term are presented.

Feasibility variables were summarized descriptively.

Estimates of sample sizes for future trials were based on the pooled SDs from both treatment arms for the following outcomes: 11β-HSD1 activity in skin (at 28 days); sudomotor function, skin hydration, epidermal barrier function, integrity (at 28 days), and recovery (TEWL at 3 hours, 2 days, and 7 days after disruption by repeat tape stripping at 28 days); skin thickness (at 35 days); and WH (maximal early granulation tissue width at 2 days and maximal blood clot depth at 7 days after biopsy at 28 days).

1.4 Changes in the Conduct of the Study or Planned Analyses

Recruitment halted early

Study recruitment was intended to continue until a total of 30 participants had been randomized to ensure recruitment of at least 12 per group, as recommended for pilot studies, with a 20% dropout rate. The recruitment period was extended twice. At the end of the second extension, 28 patients had been recruited, and the dropout rate, at less than 5%, was lower than expected. Therefore, we decided to halt recruitment rather than extend it further, which would have increased the risk of delaying the reporting of the trial results and the planning of future trials based on these results. This decision was made without reference to the primary outcome measure, whose results had not yet been processed, and before the breaking of the blind.

Primary outcome unit of measurement

After the completion of the study and final database lock, the primary outcome was found to have been calculated as percent conversion per *24 hours*, rather than *per hour* as stipulated in the protocol. As this difference in scale affected all values equally and did not affect the conclusions, the sponsor and investigators agreed that the values would not be changed and would be reported as percent conversion per 24 hours.

Validation of primary outcome by ELISA

In a protocol amendment, validation of the radioimmunoassay method of measuring 11β-HSD1 activity in the skin by a cortisol ELISA was added. This addition was made before the processing of the biopsy samples and the breaking of the blind.

Measurement of wound depth instead of diameter at days 7 and 35

At 2 days after wounding, maximal early granulation tissue width (a marker of early healing) was prespecified as the standardized indicator of wound diameter. However, the tissue width had reduced to zero in all patients by 7 days after wounding. Therefore, maximal clot depth (a

marker of later healing that was absent at 2 days after wounding) was substituted as the standardized indicator of healing at this time point

Per-protocol analysis

Only one participant who had withdrawn from follow-up due to work commitments after day 7 was excluded from the per-protocol dataset. Because the per-protocol analysis of each outcome was designed to include only participants in the per-protocol population who had data available for that outcome, the per-protocol analysis was essentially identical to the planned available case sensitivity analysis.

Additional sensitivity analysis of multiply imputed data

An additional planned sensitivity analysis that would have increased or decreased imputed values in multiples of the baseline SD in the observed data was not performed because of the low level of missing data.

1.5 Analysis populations

Safety population

The safety population or safety set includes all participants who received any amount of the planned study medication.

Efficacy population

The efficacy population or full analysis set includes all participants who were randomized and received at least one dose of the planned study medication.

Per-protocol efficacy population

The per-protocol efficacy population includes all participants in the efficacy population, except for those who met the following criteria:

- Receipt of prohibited prior, concomitant, or prior and concomitant medications
- Failure to meet inclusion or exclusion criteria (i.e., those who entered the study in error)
- Overall compliance with study treatment during the trial less than 80%
- No receipt of study treatment to which they were assigned through randomization
- Withdrawal from study treatment for any reason

For each primary and secondary variable, at each visit, only participants in the per protocol population with data available were to be included in the per protocol analysis.
