# Supplementary material for: Oral 11β-HSD1 inhibitor AZD4017 improves wound healing and skin integrity in adults with type 2 diabetes mellitus: a pilot randomized controlled trial
Source: Eur J Endocrinol. 2022 Feb 3;186(4):441–55. doi: 10.1530/EJE-21-1197 (PMC8942338; doi:10.1530/EJE-21-1197)
Supplement: Table S1: Full descriptive data for primary and secondary efficacy variables in the full analysis set [file supplementary_table_1.pdf]

Table S1: Full descriptive data for primary and secondary efficacy variables in the full analysis set

Variable	Summary	Placebo n=14	AZD4017 n=14
**Baseline**			
11bHSD1 activity radioassay (percent conversion per 24 hours): Day 0	Mean (SD)	15.84 (6.80)	13.73 (6.02)
	Median (Q1, Q3)	15.25 (11.60, 18.40)	10.70 (9.40, 17.40)
	Minimum, maximum	5.20, 29.30	7.80, 25.70
	Nn	14	14
11bHSD1 activity ELISA (percent conversion per 24 hours): Day 0	Mean (SD)	11.04 (8.06)	6.56 (3.38)
	Median (Q1, Q3)	6.80 (5.50, 15.60)	6.45 (4.20, 8.50)
	Minimum, maximum	3.00, 26.90	1.40, 13.60
	Nn	14	14
Sudomotor function Left Hand (µS): Day 0	Mean (SD)	54.14 (15.04)	56.43 (17.49)
	Median (Q1, Q3)	54.00 (47.00, 63.00)	57.50 (39.00, 71.00)
	Minimum, maximum	18.00, 75.00	30.00, 81.00
	Nn	14	14
Sudomotor function Right Hand (µS): Day 0	Mean (SD)	51.43 (15.91)	54.29 (16.99)
	Median (Q1, Q3)	54.00 (47.00, 60.00)	56.50 (41.00, 67.00)
	Minimum, maximum	16.00, 73.00	22.00, 80.00
	Nn	14	14
Sudomotor function Hands (µS): Day 0	Mean (SD)	52.79 (15.20)	55.36 (17.13)
	Median (Q1, Q3)	53.75 (48.00, 60.50)	56.75 (40.00, 70.50)
	Minimum, maximum	17.00, 74.00	26.00, 80.50

Variable	Summary	Placebo n=14	AZD4017 n=14
	Nn	14	14
Sudomotor function Left Foot (µS): Day 0	Mean (SD)	63.29 (17.18)	73.93 (15.62)
	Median (Q1, Q3)	65.50 (46.00, 78.00)	79.00 (63.00, 84.00)
	Minimum, maximum	38.00, 87.00	34.00, 92.00
	Nn	14	14
Sudomotor function Right Foot (µS): Day 0	Mean (SD)	63.57 (18.00)	71.36 (18.94)
	Median (Q1, Q3)	69.50 (48.00, 79.00)	77.00 (69.00, 82.00)
	Minimum, maximum	37.00, 87.00	14.00, 88.00
	Nn	14	14
Sudomotor function Feet (µS): Day 0	Mean (SD)	63.43 (17.49)	72.64 (17.08)
	Median (Q1, Q3)	67.50 (44.50, 76.50)	78.00 (64.00, 83.50)
	Minimum, maximum	37.50, 87.00	24.00, 90.00
	Nn	14	14
Sudomotor function overall (µS): Day 0	Mean (SD)	58.11 (14.71)	64.00 (15.11)
	Median (Q1, Q3)	61.00 (46.75, 67.75)	66.63 (63.25, 71.25)
	Minimum, maximum	27.25, 80.00	25.00, 84.00
	Nn	14	14
Skin hydration (A.U): Day 0	Mean (SD)	40.88 (7.79)	40.79 (9.19)
	Median (Q1, Q3)	40.47 (34.50, 46.18)	40.35 (36.68, 45.51)
	Minimum, maximum	27.17, 54.06	20.58, 58.68
	Nn	14	14
Epidermal thickness (µm): Day 0	Mean (SD)	61.30 (10.53)	65.96 (9.81)

Variable	Summary	Placebo n=14	AZD4017 n=14
	Median (Q1, Q3)	62.76 (54.51, 69.22)	65.70 (60.74, 69.32)
	Minimum, maximum	37.18, 77.57	44.08, 86.48
	Nn	14	14
Cortisol (mcg/24 hours): Day 0	Mean (SD)	81.50 (49.27)	70.50 (22.24)
	Median (Q1, Q3)	68.50 (40.00, 101.00)	75.50 (48.00, 84.00)
	Minimum, maximum	25.00, 202.00	39.00, 116.00
	Nn	14	14
Urinary [THF+alloTHF]/THE ratio: Day 0	Mean (SD)	1.06 (0.38)	0.91 (0.22)
	Median (Q1, Q3)	0.96 (0.81, 1.23)	0.97 (0.80, 1.06)
	Minimum, maximum	0.50, 1.99	0.45, 1.24
	Nn	14	14
Hour 0 TEWL (Set 1, Day 0)	Mean (SD)	8.96 (3.94)	9.61 (3.73)
	Geometric mean	8.24	9.05
	Median (Q1, Q3)	8.55 (5.10, 10.70)	8.90 (6.80, 11.30)
	Minimum, maximum	4.70, 17.60	5.50, 19.30
	Nn	14	13
Number of tapes required for barrier disruption: Day 0	Mean (SD)	47.14 (15.21)	55.54 (26.52)
	Geometric mean	44.49	50.48
	Median (Q1, Q3)	49.50 (33.00, 60.00)	51.00 (42.00, 58.00)
	Minimum, maximum	19.00, 66.00	22.00, 116.00
	Nn	14	13
**Follow-up**			

Variable	Summary	Placebo<br>n=14	AZD4017<br>n=14
11bHSD1 activity radioassay (% conv/24hrs): Day 28	Mean (SD)	12.38 (4.51)	12.58 (5.64)
	Median (Q1, Q3)	12.00 (8.90, 14.50)	12.70 (8.80, 15.60)
	Minimum, maximum	7.10, 23.40	3.40, 21.70
	Nn	13	14
11bHSD1 activity ELISA (% conv/24hrs): Day 28	Mean (SD)	15.27 (37.27)	3.81 (1.78)
	Median (Q1, Q3)	5.60 (1.00, 7.60)	4.30 (3.10, 5.00)
	Minimum, maximum	0.80, 138.70	0.72, 6.50
	Nn	13	14
Sudomotor function Left Hand (µS): Day 35	Mean (SD)	56.46 (11.12)	62.85 (14.35)
	Median (Q1, Q3)	58.00 (48.00, 63.00)	64.00 (59.00, 72.00)
	Minimum, maximum	39.00, 73.00	28.00, 81.00
	Nn	13	13
Sudomotor function Right Hand (µS): Day 35	Mean (SD)	54.08 (13.32)	59.92 (14.79)
	Median (Q1, Q3)	55.00 (51.00, 60.00)	63.00 (57.00, 69.00)
	Minimum, maximum	29.00, 74.00	23.00, 77.00
	Nn	13	13
Sudomotor function Hands (µS): Day 35	Mean (SD)	55.27 (11.97)	61.38 (14.43)
	Median (Q1, Q3)	57.50 (49.50, 61.00)	63.50 (59.00, 70.50)
	Minimum, maximum	35.50, 71.50	25.50, 79.00
	Nn	13	13
Sudomotor function Left Foot (µS): Day 35	Mean (SD)	68.69 (10.91)	70.92 (18.61)
	Median (Q1, Q3)	70.00 (61.00, 76.00)	80.00 (64.00, 82.00)

Variable	Summary	Placebo n=14	AZD4017 n=14
	Minimum, maximum	49.00, 85.00	23.00, 88.00
	Nn	13	13
Sudomotor function Right Foot (µS): Day 35	Mean (SD)	69.31 (9.87)	70.31 (21.44)
	Median (Q1, Q3)	65.00 (63.00, 75.00)	80.00 (68.00, 82.00)
	Minimum, maximum	51.00, 85.00	10.00, 85.00
	Nn	13	13
Sudomotor function Feet (µS): Day 35	Mean (SD)	69.00 (10.19)	70.62 (19.92)
	Median (Q1, Q3)	68.50 (62.00, 75.00)	80.00 (66.00, 82.50)
	Minimum, maximum	50.00, 85.00	16.50, 84.50
	Nn	13	13
Sudomotor function Overall (µS): Day 35	Mean (SD)	62.13 (10.01)	66.00 (15.12)
	Median (Q1, Q3)	59.75 (53.75, 68.00)	70.75 (64.50, 73.00)
	Minimum, maximum	49.25, 77.50	21.00, 78.75
	Nn	13	13
Skin hydration (A.U): Day 35	Mean (SD)	38.19 (9.69)	44.82 (10.10)
	Median (Q1, Q3)	40.52 (29.40, 46.04)	45.28 (37.02, 46.53)
	Minimum, maximum	23.74, 52.86	30.53, 69.72
	Nn	12	12
Epidermal thickness (µm): Day 35	Mean (SD)	61.72 (8.35)	66.07 (10.49)
	Median (Q1, Q3)	57.54 (55.69, 67.30)	66.29 (60.57, 74.37)
	Minimum, maximum	51.32, 77.40	49.13, 82.95
	Nn	13	12

Variable	Summary	Placebo<br>n=14	AZD4017<br>n=14
Cortisol (mcg/24 hours): Day 35	Mean (SD)	81.62 (44.49)	64.08 (22.04)
	Median (Q1, Q3)	55.00 (47.00, 120.00)	62.00 (52.00, 73.00)
	Minimum, maximum	29.00, 164.00	34.00, 124.00
	Nn	13	13
Urinary [THF+alloTHF]/THE ratio: Day 35	Mean (SD)	1.08 (0.36)	0.10 (0.03)
	Median (Q1, Q3)	0.89 (0.88, 1.24)	0.10 (0.08, 0.12)
	Minimum, maximum	0.75, 2.07	0.04, 0.16
	Nn	13	13
Wound gap diameter (mm): Day 2	Mean (SD)	1.49 (0.72)	0.98 (0.70)
	Median (Q1, Q3)	1.64 (1.05, 2.02)	0.88 (0.52, 1.29)
	Minimum, maximum	0.00, 2.51	0.00, 2.42
	Nn	14	14
Wound depth (mm): Day 7	Mean (SD)	0.60 (0.23)	0.59 (0.16)
	Median (Q1, Q3)	0.66 (0.57, 0.77)	0.59 (0.57, 0.68)
	Minimum, maximum	0.00, 0.82	0.27, 0.86
	Nn	14	14
Wound gap diameter (mm): Day 30	Mean (SD)	1.44 (0.70)	0.65 (0.49)
	Median (Q1, Q3)	1.41 (1.14, 1.98)	0.81 (0.25, 0.98)
	Minimum, maximum	0.00, 2.44	0.00, 1.43
	Nn	11	12
Wound depth (mm): Day 35	Mean (SD)	0.60 (0.17)	0.54 (0.21)
	Median (Q1, Q3)	0.63 (0.55, 0.67)	0.54 (0.47, 0.63)

Variable	Summary	Placebo n=14	AZD4017 n=14
	Minimum, maximum	0.27, 0.90	0.19, 0.94
	Nn	13	12
Biopsy AZD4017: Day 28	Mean (SD)	<5.00 (0.00)	1685.07 (914.43)
	Geometric mean	<5.00	1442.44
	Median (Q1, Q3)	<5.00 (<5.00, <5.00)	1570.00 (876.00, 2440.00)
	Minimum, maximum	<5.00, <5.00	443.00, 3310.00
	Nn	13	14
Plasma AZD4017: Day 35	Mean (SD)	<5.00 (0.00)	6992.50 (5303.82)
	Geometric mean	<5.00	5281.66
	Median (Q1, Q3)	<5.00 (<5.00, <5.00)	6490.00 (2960.00, 9040.00)
	Minimum, maximum	<5.00, <5.00	1180.00, 19400.00
	Nn	7	12
Hour 3 TEWL (Set 1; Day 0)	Mean (SD)	36.03 (9.66)	31.68 (8.00)
	Geometric mean	34.94	30.84
	Median (Q1, Q3)	32.50 (29.30, 43.20)	31.50 (26.10, 35.50)
	Minimum, maximum	26.30, 53.80	22.60, 50.70
	Nn	14	12
Hour 48 TEWL (Set 1; Day 2)	Mean (SD)	20.53 (5.64)	22.98 (10.04)
	Geometric mean	19.79	21.29
	Median (Q1, Q3)	19.90 (16.10, 26.00)	21.40 (15.40, 25.80)
	Minimum, maximum	12.40, 29.80	11.40, 47.80
	Nn	14	11

Variable	Summary	Placebo<br>n=14	AZD4017<br>n=14
Hour 168 TEWL (Set 1; Day 7)	Mean (SD)	13.95 (3.52)	21.22 (18.23)
	Geometric mean	13.55	16.06
	Median (Q1, Q3)	14.90 (11.10, 15.90)	14.50 (9.40, 24.70)
	Minimum, maximum	9.00, 21.10	6.90, 60.60
	Nn	13	13
Hour 3 TEWL (Set 2; Day 28)	Mean (SD)	26.35 (11.63)	31.17 (9.81)
	Geometric mean	23.77	29.83
	Median (Q1, Q3)	27.80 (19.50, 28.60)	32.80 (21.40, 37.40)
	Minimum, maximum	9.00, 52.10	18.70, 54.90
	Nn	13	14
Hour 48 TEWL (Set 2; Day 30)	Mean (SD)	15.65 (5.99)	19.64 (9.12)
	Geometric mean	14.43	18.21
	Median (Q1, Q3)	15.30 (13.20, 19.50)	16.80 (14.90, 19.60)
	Minimum, maximum	6.50, 25.30	11.70, 40.50
	Nn	13	14
Hour 168 TEWL (Set 2; Day 35)	Mean (SD)	10.72 (4.01)	11.72 (4.83)
	Geometric mean	10.02	10.84
	Median (Q1, Q3)	10.50 (8.20, 12.40)	10.40 (8.80, 15.10)
	Minimum, maximum	4.70, 18.20	4.90, 20.40
	Nn	13	12
Hour 0 TEWL (Set 3; Day 35)	Mean (SD)	7.45 (2.82)	9.78 (4.35)
	Geometric mean	6.99	8.87

Variable	Summary	Placebo<br>n=14	AZD4017<br>n=14
	Median (Q1, Q3)	7.00 (5.40, 9.10)	9.70 (7.70, 12.50)
	Minimum, maximum	3.60, 14.10	4.40, 18.30
	Nn	13	12
Number of tapes required for barrier disruption: Day 28	Mean (SD)	42.85 (19.79)	60.14 (25.72)
	Geometric mean	37.88	55.55
	Median (Q1, Q3)	40.00 (25.00, 54.00)	55.00 (41.00, 70.00)
	Minimum, maximum	11.00, 77.00	30.00, 120.00
	Nn	13	14

Nn, number non-missing; Q1, first quartile; Q3, third quartile.

Table S2: Full descriptive data for laboratory safety variables in the safety set

Variable	Summary	Day 0		Day 7		Day 28		Day 35		Day 42	
		**Placebo**	**AZD4017**	**Placebo**	**AZD4017**	**Placebo**	**AZD4017**	**Placebo**	**AZD4017**	**Placebo**	**AZD4017**
		**n=14**	**n=14**	**n=14**	**n=14**	**n=14**	**n=14**	**n=14**	**n=14**	**n=14**	**n=14**
Body mass index (kg/m^2)	Mean (SD)	33.67 (13.47)	35.05 (5.68)					34.07 (14.56)	35.71 (6.43)		
	Median	31.02	34.54					31.35	35.06		
	(Q1, Q3)	(26.52, 33.57)	(30.65, 39.08)					(26.51, 32.93)	(31.38, 38.61)		
	Minimum, maximum	22.59, 75.64	27.04, 46.54					22.85, 77.85	27.35, 51.80		
	Nn	14	14					13	13		
Waist-hip ratio	Mean (SD)	0.98 (0.08)	1.03 (0.08)					0.98 (0.07)	1.02 (0.07)		
	Median	0.98	1.03					0.98	1.01		
	(Q1, Q3)	(0.92, 1.05)	(0.95, 1.10)					(0.91, 1.04)	(0.97, 1.07)		
	Minimum, maximum	0.85, 1.13	0.92, 1.17					0.89, 1.09	0.92, 1.16		
	Nn	14	14					13	13		
Systolic blood pressure (mm Hg)	Mean (SD)	135.71 (21.01)	140.43 (12.02)					143.54 (12.07)	128.62 (11.23)	136.92 (13.12)	137.50 (10.97)
	Median	136.00	140.00					148.00	126.00	137.00	140.00
	(Q1, Q3)	(120.00, 145.00)	(131.00, 150.00)					(140.00, 152.00)	(123.00, 134.00)	(128.00, 146.00)	(129.00, 146.00)
	Minimum, maximum	101.00, 174.00	120.00, 162.00					121.00, 159.00	106.00, 154.00	118.00, 158.00	113.00, 150.00
	Nn	14	14					13	13	13	14
Diastolic blood pressure (mm Hg)	Mean (SD)	83.86 (8.91)	72.64 (9.96)					79.62 (7.11)	73.69 (7.88)	80.00 (10.04)	79.29 (11.36)
	Median	83.00	74.00					80.00	73.00	80.00	77.00

Variable	Summary	Day 0		Day 7		Day 28		Day 35		Day 42	
		**Placebo**	**AZD4017**	**Placebo**	**AZD4017**	**Placebo**	**AZD4017**	**Placebo**	**AZD4017**	**Placebo**	**AZD4017**
		**n=14**	**n=14**	**n=14**	**n=14**	**n=14**	**n=14**	**n=14**	**n=14**	**n=14**	**n=14**
	(Q1, Q3)	(77.00, 90.00)	(63.00, 84.00)					(75.00, 84.00)	(70.00, 78.00)	(77.00, 86.00)	(71.00, 89.00)
	Minimum, maximum	68.00, 99.00	60.00, 86.00					67.00, 92.00	61.00, 91.00	59.00, 94.00	60.00, 103.00
	Nn	14	14					13	13	13	14
HbA1c (mmol/mol)	Mean (SD)	72.29 (19.43)	66.00 (14.91)	73.77 (18.09)	64.31 (14.60)	68.92 (17.09)	66.00 (15.66)	70.00 (20.10)	63.67 (16.27)	68.46 (17.76)	65.43 (16.31)
	Median	72.00	64.00	71.00	63.00	67.00	69.00	71.00	68.00	69.00	68.00
	(Q1, Q3)	(54.00, 90.00)	(59.00, 82.00)	(56.00, 90.00)	(57.00, 71.00)	(55.00, 85.00)	(54.00, 81.00)	(49.00, 86.00)	(52.00, 77.00)	(55.00, 81.00)	(53.00, 78.00)
	Minimum, maximum	46.00, 100.00	42.00, 86.00	49.00, 98.00	41.00, 85.00	46.00, 98.00	42.00, 87.00	45.00, 108.00	42.00, 86.00	44.00, 109.00	42.00, 90.00
	Nn	14	14	13	13	13	14	11	12	13	14
High density lipoprotein (mmol/l)	Mean (SD)	1.24 (0.31)	1.19 (0.27)	1.16 (0.29)	1.05 (0.25)	1.22 (0.30)	1.11 (0.25)	1.19 (0.30)	0.98 (0.26)	1.18 (0.36)	1.22 (0.28)
	Median	1.20	1.20	1.10	1.10	1.10	1.10	1.10	0.90	1.20	1.30
	(Q1, Q3)	(1.00, 1.30)	(0.90, 1.40)	(0.90, 1.30)	(0.80, 1.20)	(1.10, 1.30)	(0.80, 1.30)	(1.10, 1.20)	(0.80, 1.10)	(1.00, 1.20)	(0.90, 1.40)
	Minimum, maximum	0.90, 2.00	0.70, 1.60	0.80, 1.80	0.70, 1.40	0.90, 1.90	0.70, 1.40	0.90, 1.90	0.60, 1.40	0.50, 1.80	0.80, 1.70
	Nn	14	14	14	14	13	14	12	12	12	13
Cholesterol (mmol/l)	Mean (SD)	4.36 (1.13)	3.95 (0.75)	4.31 (1.00)	3.59 (0.64)	4.15 (0.91)	3.54 (0.59)	4.17 (0.88)	3.44 (0.64)	4.06 (1.01)	3.81 (0.61)
	Median	4.40	3.90	4.30	3.40	4.10	3.40	4.10	3.40	4.20	3.80
	(Q1, Q3)	(3.60, 4.60)	(3.40, 4.40)	(3.40, 4.50)	(3.20, 4.20)	(3.70, 4.50)	(3.20, 3.70)	(3.80, 4.60)	(3.00, 3.70)	(3.70, 4.40)	(3.40, 4.20)
	Minimum, maximum	3.10, 7.50	2.60, 5.50	3.10, 6.70	2.60, 4.90	2.70, 6.30	2.60, 4.90	2.80, 6.20	2.70, 4.80	2.00, 6.00	2.80, 5.10
	Nn	14	14	14	14	13	14	12	12	12	13
Triglycerides (mmol/l)	Mean (SD)	1.68 (0.74)	2.02 (1.02)	2.11 (0.70)	2.39 (1.58)	1.80 (0.86)	1.71 (0.75)	2.23 (1.02)	2.38 (2.22)	1.63 (0.78)	1.89 (1.31)
	Median	1.60	1.50	2.00	2.00	1.50	1.60	2.00	1.80	1.70	1.60

Variable	Summary	Day 0		Day 7		Day 28		Day 35		Day 42	
		**Placebo**	**AZD4017**	**Placebo**	**AZD4017**	**Placebo**	**AZD4017**	**Placebo**	**AZD4017**	**Placebo**	**AZD4017**
		**n=14**	**n=14**	**n=14**	**n=14**	**n=14**	**n=14**	**n=14**	**n=14**	**n=14**	**n=14**
	(Q1, Q3)	(1.10, 1.90)	(1.40, 2.50)	(1.70, 2.70)	(1.70, 2.30)	(1.20, 2.00)	(1.00, 2.00)	(1.60, 2.80)	(1.40, 2.10)	(1.00, 1.90)	(1.20, 2.00)
	Minimum, maximum	1.10, 3.90	1.10, 4.70	0.70, 3.30	1.10, 7.10	1.00, 3.70	1.00, 3.40	0.80, 3.90	0.90, 8.90	0.70, 3.20	0.80, 6.00
	Nn	14	14	14	14	13	14	12	12	12	13
Hemoglobin (g/l)	Mean (SD)	139.21 (14.12)	139.43 (11.35)	136.07 (13.04)	137.43 (10.80)	137.46 (10.11)	138.36 (10.58)	134.42 (12.91)	136.31 (11.50)	135.31 (13.19)	139.86 (13.42)
	Median	138.00	139.00	136.00	135.00	138.00	136.00	133.00	134.00	139.00	140.00
	(Q1, Q3)	(130.00, 146.00)	(130.00, 145.00)	(126.00, 145.00)	(131.00, 144.00)	(132.00, 145.00)	(134.00, 144.00)	(126.00, 144.00)	(129.00, 140.00)	(130.00, 145.00)	(131.00, 145.00)
	Minimum, maximum	112.00, 168.00	121.00, 162.00	115.00, 161.00	122.00, 163.00	113.00, 153.00	124.00, 164.00	110.00, 155.00	122.00, 162.00	106.00, 151.00	117.00, 162.00
	Nn	14	14	14	14	13	14	12	13	13	14
White blood cells ($x10^9$/l)	Mean (SD)	6.46 (2.55)	7.34 (2.04)	6.71 (1.83)	7.97 (2.12)	6.54 (2.11)	6.72 (1.57)	6.69 (1.96)	7.04 (1.94)	6.51 (1.97)	7.66 (2.18)
	Median	6.62	7.36	6.19	8.40	6.44	6.63	7.01	7.09	6.42	7.69
	(Q1, Q3)	(4.59, 8.33)	(6.09, 8.74)	(5.41, 8.00)	(6.47, 9.54)	(5.81, 7.48)	(6.05, 7.43)	(5.06, 7.78)	(5.85, 8.59)	(5.73, 6.82)	(7.00, 8.68)
	Minimum, maximum	2.13, 11.62	3.21, 11.27	4.11, 10.35	3.83, 10.91	2.33, 10.21	3.30, 9.34	3.42, 10.49	3.58, 9.90	3.19, 10.24	3.65, 12.00
	Nn	14	14	14	14	13	14	12	13	13	14
Platelets ($x10^9$/l)	Mean (SD)	218.71 (61.85)	273.14 (81.37)	219.36 (64.89)	275.29 (81.60)	222.69 (59.57)	288.57 (99.85)	215.92 (66.59)	272.69 (85.96)	221.77 (60.01)	281.43 (92.00)
	Median	204.00	264.00	214.00	264.00	190.00	286.00	204.00	260.00	203.00	270.00
	(Q1, Q3)	(171.00, 259.00)	(232.00, 335.00)	(171.00, 264.00)	(223.00, 317.00)	(180.00, 262.00)	(221.00, 331.00)	(171.00, 212.00)	(245.00, 285.00)	(170.00, 254.00)	(205.00, 349.00)
	Minimum, maximum	149.00, 352.00	117.00, 402.00	146.00, 381.00	130.00, 428.00	146.00, 325.00	124.00, 476.00	149.00, 365.00	136.00, 442.00	155.00, 357.00	133.00, 446.00
	Nn	14	14	14	14	13	14	12	13	13	14
Red blood cells ($x10^{12}$/l)	Mean (SD)	4.84 (0.49)	4.71 (0.48)	4.75 (0.54)	4.59 (0.45)	4.77 (0.39)	4.68 (0.47)	4.66 (0.47)	4.57 (0.44)	4.68 (0.49)	4.77 (0.53)

Variable	Summary	Day 0		Day 7		Day 28		Day 35		Day 42	
		Placebo	AZD4017	Placebo	AZD4017	Placebo	AZD4017	Placebo	AZD4017	Placebo	AZD4017
		n=14	n=14	n=14	n=14	n=14	n=14	n=14	n=14	n=14	n=14
	Median	4.80	4.65	4.79	4.54	4.70	4.65	4.65	4.58	4.55	4.76
	(Q1, Q3)	(4.46, 5.17)	(4.56, 5.05)	(4.27, 5.11)	(4.39, 4.87)	(4.52, 4.89)	(4.44, 4.78)	(4.25, 5.01)	(4.38, 4.81)	(4.41, 4.98)	(4.59, 4.92)
	Minimum, maximum	4.13, 5.78	3.59, 5.68	3.93, 5.66	3.57, 5.53	4.24, 5.62	3.74, 5.60	4.01, 5.42	3.57, 5.43	3.96, 5.53	3.62, 5.97
	Nn	14	14	14	14	13	14	12	13	13	14
Mean corpuscular volume (fl)	Mean (SD)	88.43 (4.64)	91.21 (6.96)	89.71 (4.39)	92.14 (6.77)	88.15 (5.41)	91.21 (6.82)	90.00 (6.30)	92.23 (6.73)	88.08 (4.57)	90.50 (6.10)
	Median	88.00	91.00	90.00	93.00	87.00	91.00	91.00	94.00	88.00	91.00
	(Q1, Q3)	(87.00, 91.00)	(86.00, 95.00)	(89.00, 92.00)	(88.00, 95.00)	(86.00, 92.00)	(84.00, 97.00)	(86.00, 93.00)	(89.00, 95.00)	(85.00, 92.00)	(84.00, 95.00)
	Minimum, maximum	79.00, 97.00	80.00, 107.00	80.00, 98.00	81.00, 105.00	79.00, 98.00	82.00, 105.00	76.00, 99.00	80.00, 104.00	80.00, 96.00	81.00, 101.00
	Nn	14	14	14	14	13	14	12	13	13	14
Hematocrit (packed cell volume)	Mean (SD)	0.43 (0.04)	0.43 (0.03)	0.43 (0.04)	0.42 (0.03)	0.42 (0.03)	0.43 (0.03)	0.42 (0.04)	0.42 (0.04)	0.41 (0.03)	0.43 (0.04)
	Median	0.42	0.43	0.42	0.41	0.41	0.42	0.42	0.41	0.40	0.43
	(Q1, Q3)	(0.40, 0.46)	(0.41, 0.45)	(0.38, 0.46)	(0.40, 0.43)	(0.40, 0.43)	(0.40, 0.45)	(0.39, 0.45)	(0.40, 0.43)	(0.39, 0.45)	(0.40, 0.45)
	Minimum, maximum	0.37, 0.50	0.37, 0.48	0.37, 0.50	0.37, 0.49	0.37, 0.48	0.38, 0.50	0.35, 0.48	0.37, 0.49	0.36, 0.47	0.36, 0.50
	Nn	14	14	14	14	13	14	12	13	13	14
Mean corpuscular hemoglobin (pg)	Mean (SD)	28.82 (2.09)	29.74 (2.47)	28.79 (1.99)	30.13 (2.50)	28.91 (2.07)	29.68 (2.12)	28.93 (2.14)	29.96 (2.56)	28.98 (2.34)	29.42 (2.15)
	Median	28.70	30.00	28.80	30.10	29.60	30.00	29.10	30.10	29.10	29.70
	(Q1, Q3)	(27.70, 29.90)	(27.30, 31.50)	(27.60, 29.90)	(27.70, 32.00)	(27.70, 29.90)	(28.20, 31.10)	(28.00, 30.10)	(27.70, 31.30)	(28.10, 30.70)	(27.70, 30.90)
	Minimum, maximum	23.70, 31.80	25.60, 34.50	24.60, 32.30	26.40, 35.00	24.00, 32.00	26.10, 33.40	23.70, 32.40	26.40, 34.50	23.60, 32.80	26.20, 33.10

Variable	Summary	Day 0		Day 7		Day 28		Day 35		Day 42	
		**Placebo n=14**	**AZD4017 n=14**	**Placebo n=14**	**AZD4017 n=14**	**Placebo n=14**	**AZD4017 n=14**	**Placebo n=14**	**AZD4017 n=14**	**Placebo n=14**	**AZD4017 n=14**
	Nn	14	14	14	14	13	14	12	13	13	14
Corpuscular hemoglobin concentration (g/l)	Mean (SD)	325.57 (10.43)	326.64 (8.29)	321.07 (11.87)	326.93 (9.03)	328.62 (13.61)	325.79 (7.76)	322.08 (11.20)	325.08 (8.65)	328.85 (15.56)	324.86 (6.69)
	Median	328.00	328.00	321.00	327.00	333.00	326.00	324.00	329.00	333.00	326.00
	(Q1, Q3)	(322.00, 334.00)	(320.00, 332.00)	(311.00, 327.00)	(316.00, 334.00)	(319.00, 337.00)	(321.00, 328.00)	(311.00, 330.00)	(320.00, 330.00)	(320.00, 338.00)	(318.00, 330.00)
	Minimum, maximum	302.00, 337.00	315.00, 345.00	307.00, 343.00	314.00, 344.00	306.00, 347.00	314.00, 344.00	303.00, 337.00	306.00, 334.00	294.00, 350.00	315.00, 336.00
	Nn	14	14	14	14	13	14	12	13	13	14
Red blood cell distribution width (%)	Mean (SD)	13.88 (0.98)	14.12 (1.10)	14.43 (1.29)	14.27 (1.12)	14.02 (0.87)	14.08 (0.82)	14.23 (1.01)	14.44 (0.88)	13.80 (0.98)	13.74 (1.04)
	Median	13.90	13.90	14.50	13.90	13.90	14.00	14.30	14.20	13.90	13.60
	(Q1, Q3)	(13.10, 14.50)	(13.20, 15.30)	(13.90, 14.80)	(13.70, 14.70)	(13.60, 14.30)	(13.80, 14.60)	(13.50, 14.40)	(14.00, 14.90)	(13.10, 14.10)	(13.40, 14.40)
	Minimum, maximum	12.40, 16.00	12.70, 15.80	12.70, 17.70	12.90, 16.80	12.50, 16.00	12.00, 15.30	13.20, 16.60	12.70, 15.90	12.50, 16.30	11.70, 15.70
	Nn	14	14	14	14	13	14	12	13	13	14
Albumin (g/l)	Mean (SD)	39.43 (2.56)	39.50 (2.44)	39.50 (2.53)	40.14 (3.23)	38.85 (2.48)	38.71 (2.89)	39.67 (2.27)	40.00 (3.00)	38.15 (1.95)	38.69 (2.18)
	Median	39.00	40.00	40.00	40.00	39.00	39.00	40.00	39.00	38.00	39.00
	(Q1, Q3)	(38.00, 41.00)	(38.00, 41.00)	(38.00, 41.00)	(38.00, 42.00)	(37.00, 41.00)	(37.00, 40.00)	(38.00, 41.00)	(38.00, 41.00)	(37.00, 40.00)	(36.00, 40.00)
	Minimum, maximum	35.00, 44.00	36.00, 44.00	35.00, 44.00	36.00, 49.00	34.00, 42.00	35.00, 46.00	36.00, 44.00	37.00, 46.00	35.00, 42.00	36.00, 42.00
	Nn	14	14	14	14	13	14	12	13	13	13
Bilirubin (umol/l)	Mean (SD)	8.93 (2.97)	8.14 (2.82)	8.36 (2.17)	8.64 (5.53)	9.69 (3.35)	9.86 (4.49)	8.92 (5.55)	8.54 (3.80)	8.31 (2.14)	8.38 (2.66)
	Median	9.00	8.00	9.00	7.00	10.00	9.00	7.00	8.00	8.00	8.00

Variable	Summary	Day 0		Day 7		Day 28		Day 35		Day 42	
		**Placebo**	**AZD4017**	**Placebo**	**AZD4017**	**Placebo**	**AZD4017**	**Placebo**	**AZD4017**	**Placebo**	**AZD4017**
		**n=14**	**n=14**	**n=14**	**n=14**	**n=14**	**n=14**	**n=14**	**n=14**	**n=14**	**n=14**
	(Q1, Q3)	(6.00, 12.00)	(6.00, 9.00)	(7.00, 10.00)	(6.00, 8.00)	(7.00, 11.00)	(7.00, 12.00)	(6.00, 9.00)	(7.00, 10.00)	(7.00, 11.00)	(7.00, 10.00)
	Minimum, maximum	5.00, 14.00	4.00, 14.00	4.00, 12.00	4.00, 24.00	5.00, 16.00	5.00, 22.00	4.00, 24.00	4.00, 19.00	5.00, 11.00	5.00, 13.00
	Nn	14	14	14	14	13	14	12	13	13	13
Alkaline phosphatase (U/l)	Mean (SD)	80.43 (23.76)	85.07 (26.08)	80.86 (27.04)	80.14 (26.38)	77.31 (21.55)	68.93 (23.03)	79.58 (21.21)	66.15 (21.10)	76.85 (25.66)	78.62 (30.06)
	Median	77.00	88.00	72.00	79.00	72.00	70.00	76.00	69.00	69.00	79.00
	(Q1, Q3)	(65.00, 95.00)	(64.00, 106.00)	(66.00, 96.00)	(57.00, 102.00)	(64.00, 86.00)	(55.00, 80.00)	(71.00, 86.00)	(55.00, 75.00)	(65.00, 77.00)	(61.00, 91.00)
	Minimum, maximum	50.00, 145.00	37.00, 123.00	49.00, 147.00	38.00, 122.00	51.00, 129.00	29.00, 114.00	47.00, 130.00	27.00, 100.00	50.00, 145.00	33.00, 152.00
	Nn	14	14	14	14	13	14	12	13	13	13
Alanine aminotransferase (iu/l)	Mean (SD)	24.64 (7.10)	27.43 (10.91)	22.93 (6.83)	26.14 (9.72)	22.69 (6.59)	23.07 (9.39)	21.67 (6.36)	21.15 (10.36)	21.15 (6.40)	24.31 (10.86)
	Median	25.00	25.00	23.00	25.00	20.00	22.00	21.00	18.00	19.00	22.00
	(Q1, Q3)	(19.00, 31.00)	(20.00, 33.00)	(19.00, 30.00)	(21.00, 27.00)	(19.00, 28.00)	(16.00, 30.00)	(18.00, 25.00)	(15.00, 23.00)	(18.00, 24.00)	(15.00, 29.00)
	Minimum, maximum	15.00, 36.00	15.00, 51.00	12.00, 34.00	14.00, 53.00	12.00, 33.00	11.00, 41.00	14.00, 33.00	10.00, 46.00	12.00, 34.00	13.00, 45.00
	Nn	14	14	14	14	13	14	12	13	13	13
Aspartate aminotransferase (iu/l)	Mean (SD)	21.00 (4.95)	22.64 (5.50)	21.07 (5.93)	22.36 (3.99)	20.77 (4.17)	21.36 (2.68)	20.92 (3.85)	20.67 (2.93)	19.00 (5.70)	22.18 (3.76)
	Median	21.00	22.00	20.00	22.00	21.00	21.00	20.00	21.00	20.00	22.00
	(Q1, Q3)	(19.00, 22.00)	(19.00, 26.00)	(17.00, 24.00)	(19.00, 24.00)	(19.00, 23.00)	(19.00, 23.00)	(19.00, 21.00)	(19.00, 23.00)	(16.00, 20.00)	(21.00, 23.00)
	Minimum, maximum	14.00, 32.00	14.00, 36.00	14.00, 36.00	18.00, 30.00	14.00, 28.00	18.00, 27.00	15.00, 30.00	16.00, 25.00	12.00, 34.00	15.00, 30.00
	Nn	14	14	14	14	13	14	12	12	12	11

Variable	Summary	Day 0		Day 7		Day 28		Day 35		Day 42	
		**Placebo**	**AZD4017**	**Placebo**	**AZD4017**	**Placebo**	**AZD4017**	**Placebo**	**AZD4017**	**Placebo**	**AZD4017**
		**n=14**	**n=14**	**n=14**	**n=14**	**n=14**	**n=14**	**n=14**	**n=14**	**n=14**	**n=14**
Gamma-glutamyl transpeptidase (iu/l)	Mean (SD)	41.86 (33.87)	39.29 (29.87)	39.71 (32.51)	37.71 (30.94)	42.46 (31.59)	29.86 (21.31)	43.67 (36.88)	23.92 (15.94)	41.23 (33.38)	29.23 (20.66)
	Median	37.00	29.00	37.00	22.00	34.00	21.00	41.00	19.00	40.00	24.00
	(Q1, Q3)	(21.00, 45.00)	(21.00, 43.00)	(19.00, 46.00)	(20.00, 46.00)	(22.00, 58.00)	(17.00, 33.00)	(19.00, 47.00)	(15.00, 24.00)	(22.00, 42.00)	(18.00, 35.00)
	Minimum, maximum	13.00, 145.00	16.00, 127.00	12.00, 143.00	13.00, 129.00	16.00, 133.00	14.00, 94.00	15.00, 147.00	10.00, 71.00	15.00, 143.00	6.00, 89.00
	Nn	14	14	14	14	13	14	12	13	13	13
eGFR (ml/min/1.73 m^2)	Mean (SD)	81.86 (9.69)	79.21 (13.59)	77.50 (12.37)	71.50 (15.24)	79.77 (11.13)	75.14 (14.09)	76.83 (15.91)	73.31 (16.69)	77.46 (11.60)	76.21 (14.73)
	Median	87.00	85.00	78.00	70.00	83.00	72.00	78.00	69.00	78.00	77.00
	(Q1, Q3)	(76.00, 90.00)	(70.00, 90.00)	(67.00, 90.00)	(55.00, 88.00)	(71.00, 90.00)	(67.00, 90.00)	(73.00, 90.00)	(62.00, 90.00)	(67.00, 90.00)	(69.00, 90.00)
	Minimum, maximum	61.00, 90.00	54.00, 90.00	55.00, 90.00	47.00, 90.00	58.00, 90.00	51.00, 90.00	39.00, 90.00	47.00, 90.00	59.00, 90.00	42.00, 90.00
	Nn	14	14	14	14	13	14	12	13	13	14
Sodium (mmol/l)	Mean (SD)	138.93 (2.06)	140.71 (5.47)	139.29 (2.30)	140.00 (2.48)	138.15 (1.82)	138.79 (2.15)	140.25 (3.47)	139.31 (1.89)	138.92 (2.02)	138.00 (2.15)
	Median	139.00	140.00	139.00	140.00	138.00	139.00	140.00	139.00	139.00	138.00
	(Q1, Q3)	(137.00, 140.00)	(137.00, 142.00)	(138.00, 142.00)	(139.00, 141.00)	(137.00, 139.00)	(137.00, 140.00)	(138.00, 142.00)	(138.00, 140.00)	(138.00, 140.00)	(137.00, 139.00)
	Minimum, maximum	136.00, 143.00	136.00, 158.00	135.00, 143.00	136.00, 144.00	135.00, 141.00	134.00, 142.00	136.00, 148.00	136.00, 143.00	135.00, 142.00	134.00, 142.00
	Nn	14	14	14	14	13	14	12	13	13	14
Potassium (mmol/l)	Mean (SD)	4.46 (0.33)	4.63 (0.41)	4.79 (0.55)	4.59 (0.37)	4.49 (0.31)	4.74 (0.41)	4.48 (0.43)	4.52 (0.28)	4.65 (0.45)	4.63 (0.33)
	Median	4.40	4.60	4.70	4.60	4.60	4.70	4.50	4.50	4.70	4.70
	(Q1, Q3)	(4.20, 4.70)	(4.40, 4.80)	(4.50, 5.00)	(4.30, 4.90)	(4.40, 4.70)	(4.40, 5.10)	(4.30, 4.80)	(4.30, 4.60)	(4.20, 4.90)	(4.60, 4.70)
	Minimum, maximum	3.90, 5.10	3.90, 5.50	3.70, 5.70	4.10, 5.30	3.80, 4.90	4.20, 5.40	3.50, 5.00	4.20, 5.10	3.90, 5.50	4.10, 5.10

Variable	Summary	Day 0		Day 7		Day 28		Day 35		Day 42	
		Placebo	AZD4017	Placebo	AZD4017	Placebo	AZD4017	Placebo	AZD4017	Placebo	AZD4017
		n=14	n=14	n=14	n=14	n=14	n=14	n=14	n=14	n=14	n=14
	Nn	14	14	14	14	13	14	12	13	13	12
Urea (mmol/l)	Mean (SD)	6.96 (2.81)	7.25 (2.17)	6.91 (2.72)	7.24 (1.89)	6.79 (3.85)	6.56 (1.80)	7.67 (5.13)	7.60 (2.72)	7.07 (2.98)	7.57 (2.72)
	Median	6.70	6.40	6.40	6.60	5.80	6.60	6.30	8.50	6.80	7.30
	(Q1, Q3)	(5.20, 7.30)	(5.90, 8.70)	(5.10, 8.90)	(5.60, 9.20)	(4.90, 6.80)	(5.30, 7.90)	(5.80, 7.40)	(5.60, 9.30)	(5.50, 7.30)	(6.10, 8.30)
	Minimum, maximum	3.90, 14.90	3.90, 11.00	2.60, 12.30	4.90, 10.60	3.10, 18.60	3.80, 9.50	2.70, 23.10	3.70, 13.30	3.00, 14.70	3.90, 15.40
	Nn	14	14	14	14	13	14	12	13	13	14
Creatinine (umol/l)	Mean (SD)	76.64 (15.36)	76.21 (20.04)	82.36 (17.90)	88.57 (22.57)	79.08 (16.81)	80.93 (19.07)	84.42 (24.67)	84.69 (21.53)	82.08 (16.96)	79.93 (17.93)
	Median	77.00	76.00	82.00	84.00	81.00	75.00	81.00	74.00	82.00	75.00
	(Q1, Q3)	(66.00, 88.00)	(60.00, 94.00)	(70.00, 97.00)	(71.00, 102.00)	(69.00, 93.00)	(67.00, 93.00)	(70.00, 87.00)	(72.00, 101.00)	(66.00, 96.00)	(70.00, 95.00)
	Minimum, maximum	49.00, 101.00	51.00, 115.00	55.00, 114.00	61.00, 129.00	55.00, 106.00	54.00, 120.00	55.00, 148.00	59.00, 125.00	59.00, 110.00	56.00, 111.00
	Nn	14	14	14	14	13	14	12	13	13	14
Testosterone (nmol/l)	Mean (SD)	11.10 (6.25)	6.97 (5.48)	10.34 (7.47)	6.43 (4.94)	11.72 (6.75)	6.46 (5.01)	10.17 (7.94)	6.03 (5.08)	10.12 (6.47)	7.66 (6.43)
	Median	10.10	8.00	10.70	7.00	11.50	6.70	9.30	5.00	9.70	8.10
	(Q1, Q3)	(8.00, 16.80)	(0.80, 11.40)	(6.20, 13.70)	(1.40, 9.90)	(8.50, 14.70)	(1.00, 10.30)	(8.00, 10.60)	(1.00, 10.00)	(6.90, 13.80)	(0.70, 12.30)
	Minimum, maximum	0.60, 22.00	0.40, 15.00	0.50, 27.50	0.70, 14.90	0.60, 27.20	0.80, 14.20	0.50, 31.50	0.60, 13.50	0.40, 25.00	0.60, 18.30
	Nn	14	14	14	14	12	14	12	13	13	14
Dehydroepiandrosterone sulphate (umol/l)	Mean (SD)	3.53 (2.19)	2.91 (1.69)	3.56 (2.36)	4.91 (3.53)	3.03 (1.86)	5.35 (2.76)	3.49 (1.90)	5.03 (3.22)	2.82 (1.70)	3.55 (2.16)
	Median	2.90	2.50	3.60	3.50	3.20	5.30	3.80	4.60	2.70	3.40
	(Q1, Q3)	(1.00, 5.00)	(1.90, 3.40)	(1.00, 5.30)	(2.90, 5.50)	(1.20, 4.60)	(3.40, 6.70)	(2.30, 4.90)	(2.90, 5.40)	(1.10, 3.40)	(2.00, 4.30)
	Minimum, maximum	1.00, 7.30	1.00, 7.90	1.00, 8.00	1.00, 14.70	1.00, 6.20	1.50, 11.60	1.00, 6.40	1.60, 12.60	1.00, 6.30	1.10, 9.70

Variable	Summary	Day 0		Day 7		Day 28		Day 35		Day 42	
		Placebo	AZD4017	Placebo	AZD4017	Placebo	AZD4017	Placebo	AZD4017	Placebo	AZD4017
		n=14	n=14	n=14	n=14	n=14	n=14	n=14	n=14	n=14	n=14
	Nn	14	14	14	14	13	14	12	13	13	14
Free thyroxine (pmol/l)	Mean (SD)	14.86 (2.20)	15.73 (1.58)	14.63 (1.72)	15.70 (2.53)	14.66 (1.61)	15.72 (1.92)	14.46 (1.43)	15.39 (1.98)	13.94 (1.47)	15.10 (1.49)
	Median	15.20	15.20	14.70	15.50	14.60	16.20	14.60	14.80	14.00	15.10
	(Q1, Q3)	(13.10, 16.40)	(14.80, 16.50)	(13.20, 15.30)	(13.90, 16.80)	(13.40, 15.60)	(14.30, 17.30)	(13.50, 14.90)	(14.40, 17.60)	(12.60, 15.00)	(13.80, 16.10)
	Minimum, maximum	11.20, 18.80	14.00, 20.00	12.10, 18.50	12.20, 21.50	12.60, 17.30	13.10, 19.00	12.70, 17.90	12.30, 18.40	12.10, 16.60	13.00, 18.80
	Nn	14	14	14	13	12	12	12	13	13	14
Thyroid-stimulating hormone (mlU/l)	Mean (SD)	1.74 (0.66)	1.72 (0.63)	1.68 (0.56)	1.88 (0.76)	1.91 (0.84)	1.76 (0.81)	1.76 (0.75)	1.72 (0.64)	1.79 (0.71)	1.76 (0.93)
	Median	1.40	1.60	1.60	1.70	1.70	1.60	1.60	1.60	1.70	1.60
	(Q1, Q3)	(1.30, 2.40)	(1.20, 2.10)	(1.30, 2.00)	(1.30, 2.00)	(1.20, 2.40)	(1.30, 2.10)	(1.20, 2.00)	(1.50, 1.90)	(1.30, 2.10)	(1.40, 2.10)
	Minimum, maximum	0.78, 2.80	0.94, 3.30	0.95, 2.90	0.97, 3.70	1.10, 3.50	0.68, 3.70	0.98, 3.40	0.62, 3.10	0.81, 3.00	0.51, 3.90
	Nn	14	14	14	13	12	12	12	13	13	14

Table S3: Study treatment (placebo or AZD4017) compliance in the full analysis set

Definition	Summary	Day 2		Day 7		Day 28		Day 30		Day 35	
		Placebo n=14	AZD4017 n=14	Placebo n=14	AZD4017 n=14	Placebo n=14	AZD4017 n=14	Placebo n=14	AZD4017 n=14	Placebo n=14	AZD4017 n=14
Percent	Mean (SD)	>99 (1)	>99 (1)	99 (1)	99 (2)	99 (3)	98 (2)	97 (6)	99 (2)	98 (5)	98 (2)
	Median (Q1, Q3)	100 (100, 100)	100 (100, 100)	100 (99, 100)	100 (98, 100)	99 (98, 100)	99 (97, 100)	99 (98, 100)	99 (98, 100)	100 (98, 100)	99 (97, 100)
	Minimum, maximum	97, 101	98, 100	96, 101	94, 101	91, 101	94, 100	81, 101	94, 101	84, 101	93, 101
	Nn	13	10	13	14	12	13	13	11	12	14
Cumulative percent	Mean (SD)	95 (17)	96 (12)	97 (7)	96 (10)	98 (3)	98 (2)	97 (6)	98 (2)	98 (5)	98 (2)
	Median (Q1, Q3)	100 (100, 100)	100 (100, 100)	100 (96, 100)	100 (89, 100)	99 (97, 100)	98 (96, 100)	99 (98, 100)	98 (98, 100)	100 (98, 100)	99 (97, 100)
	Minimum, maximum	50, 125	63, 100	79, 107	71, 107	89, 102	93, 100	78, 102	93, 101	84, 101	93, 101
	Nn	13	10	13	14	12	13	13	11	12	14

Nn, number non-missing; Q1, first quartile; Q3, third quartile.

Table S4: Primary and secondary efficacy outcomes; unadjusted differences in final values between treatment groups in the full analysis set

Multiple imputation was used to address missing data. For TEWL, integrity, wound depth and diameter, linear regression was used to estimate CIs around differences between the groups. TEWL and integrity measurements were log-transformed before analysis; differences are expressed as ratios of geometric means (AZD4017:placebo). For the remaining variables, which did not meet assumptions for linear regression, median regression was used.

Variable	Median*		Difference*	Confidence interval*				
	Placebo n=14	AZD4017 n=14		75%	80%	85%	90%	95%
11bHSD1 activity radioassay (percent conversion per 24 hours): Day 28	12.18	12.70	0.52	(-2.85, 3.90)	(-3.25, 4.30)	(-3.73, 4.78)	(-4.37, 5.42)	(-5.38, 6.43)
11bHSD1 activity ELISA (percent conversion per 24 hours): Day 28	5.84	4.30	-1.54	(-3.58, 0.49)	(-3.82, 0.73)	(-4.12, 1.03)	(-4.50, 1.41)	(-5.12, 2.03)
Sudomotor function Left Hand (μS): Day 35	58.00	63.15	5.15	(-2.52, 12.82)	(-3.42, 13.72)	(-4.52, 14.82)	(-5.98, 16.28)	(-8.28, 18.58)
Sudomotor function Right Hand (μS): Day 35	54.80	62.60	7.80	(0.35, 15.25)	(-0.53, 16.13)	(-1.60, 17.20)	(-3.02, 18.62)	(-5.26, 20.86)
Sudomotor function Hands (μS): Day 35	57.02	62.80	5.78	(-2.14, 13.69)	(-3.08, 14.63)	(-4.22, 15.77)	(-5.73, 17.28)	(-8.11, 19.66)
Sudomotor function Left Foot (μS): Day 35	69.40	76.20	6.80	(-1.72, 15.32)	(-2.73, 16.33)	(-3.95, 17.55)	(-5.57, 19.17)	(-8.13, 21.73)
Sudomotor function Right Foot (μS): Day 35	70.10	79.05	8.95	(0.86, 17.04)	(-0.10, 18.00)	(-1.27, 19.17)	(-2.82, 20.72)	(-5.27, 23.17)
Sudomotor function Feet (μS): Day 35	69.95	79.53	9.57	(1.75, 17.40)	(0.83, 18.32)	(-0.29, 19.44)	(-1.78, 20.93)	(-4.12, 23.27)
Sudomotor function Overall (μS): Day 35	62.48	70.58	8.10	(1.07, 15.13)	(0.24, 15.96)	(-0.77, 16.97)	(-2.11, 18.31)	(-4.22, 20.42)
Skin hydration (A.U): Day 35	40.17	45.39	5.22	(-0.64, 11.07)	(-1.33, 11.76)	(-2.17, 12.60)	(-3.29, 13.72)	(-5.05, 15.48)
Epidermal thickness (μm): Day 35	60.32	66.94	6.62	(-1.16, 14.41)	(-2.09, 15.34)	(-3.23, 16.47)	(-4.74, 17.99)	(-7.16, 20.40)

Cortisol (mcg/24 hours): Day 35	63.25	62.40	-0.85	(-28.55, 26.85)	(-31.96, 30.26)	(-36.19, 34.49)	(-41.91, 40.21)	(-51.28, 49.58)
Urinary [THF+alloTHF]/THE ratio: Day 35	0.91	0.10	-0.81	(-0.90, -0.72)	(-0.91, -0.70)	(-0.92, -0.69)	(-0.94, -0.67)	(-0.97, -0.64)

**Variable**	**Mean***		**Difference***	**Confidence interval***				
	Placebo n=14	AZD4017 n=14		75%	80%	85%	90%	95%
Wound gap diameter (mm): Day 2	1.49	0.98	-0.51	(-0.83, -0.20)	(-0.87, -0.16)	(-0.91, -0.11)	(-0.97, -0.05)	(-1.07, 0.04)
Wound depth (mm): Day 7	0.60	0.59	-0.01	(-0.10, 0.08)	(-0.11, 0.09)	(-0.12, 0.10)	(-0.14, 0.12)	(-0.17, 0.15)
Wound gap diameter (mm): Day 30	1.38	0.67	-0.71	(-1.02, -0.39)	(-1.06, -0.35)	(-1.10, -0.31)	(-1.16, -0.25)	(-1.26, -0.15)
Wound depth (mm): Day 35	0.61	0.54	-0.06	(-0.15, 0.02)	(-0.16, 0.04)	(-0.17, 0.05)	(-0.19, 0.06)	(-0.22, 0.09)

**Variable**	**Geometric mean***		**Ratio***	**Confidence interval***				
	Placebo n=14	AZD4017 n=14		75%	80%	85%	90%	95%
Hour 3 TEWL (Set 1; Day 0)	34.94	32.00	0.92	(0.82, 1.03)	(0.80, 1.04)	(0.79, 1.06)	(0.77, 1.08)	(0.75, 1.12)
Hour 48 TEWL (Set 1; Day 2)	19.79	21.37	1.08	(0.92, 1.27)	(0.90, 1.29)	(0.88, 1.32)	(0.86, 1.36)	(0.82, 1.43)
Hour 168 TEWL (Set 1; Day 7)	13.52	16.36	1.21	(0.94, 1.56)	(0.91, 1.61)	(0.88, 1.67)	(0.84, 1.75)	(0.77, 1.89)
Hour 3 TEWL (Set 2; Day 28)	23.52	29.83	1.27	(1.05, 1.53)	(1.03, 1.57)	(1.00, 1.61)	(0.96, 1.67)	(0.91, 1.76)
Hour 48 TEWL (Set 2; Day 30)	14.65	18.21	1.24	(1.03, 1.49)	(1.01, 1.53)	(0.99, 1.57)	(0.95, 1.62)	(0.90, 1.72)
Hour 168 TEWL (Set 2; Day 35)	9.98	10.88	1.09	(0.90, 1.31)	(0.89, 1.34)	(0.86, 1.38)	(0.83, 1.43)	(0.79, 1.51)
Hour 0 TEWL (Set 3; Day 35)	7.09	9.00	1.27	(1.05, 1.54)	(1.02, 1.58)	(0.99, 1.62)	(0.96, 1.68)	(0.90, 1.78)
Number of tapes required for barrier disruption: Day 28	38.58	55.55	1.44	(1.16, 1.79)	(1.13, 1.84)	(1.09, 1.90)	(1.05, 1.98)	(0.98, 2.11)

*Estimated in imputed data

Table S5: Sensitivity analysis of primary and secondary efficacy outcomes re-imputed after QC fails and outliers removed in the full analysis set

Data were re-imputed after deleting two baseline radioassays which failed QC and one outlying ELISA assay result at day 28, TEWL readings that were potentially unreliable due to high temperatures and TEWL and WH measures which were collected earlier than scheduled. The comparison was adjusted for each variable’s baseline value (this was not available for wound diameter and depth), age, sex and baseline HbA1c. All TEWL readings were adjusted using pre-disruption TEWL at baseline.

Variable	Median*		Difference*	Confidence interval*				
	Placebo n=14	AZD4017 n=14		75%	80%	85%	90%	95%
11bHSD1 activity radioassay (percent conversion per 24 hours): Day 28	11.42	12.88	1.47	(-1.67, 4.60)	(-2.04, 4.97)	(-2.50, 5.43)	(-3.10, 6.03)	(-4.07, 7.00)
11bHSD1 activity ELISA (percent conversion per 24 hours): Day 28	4.14	4.34	0.20	(-2.11, 2.51)	(-2.39, 2.79)	(-2.74, 3.13)	(-3.20, 3.59)	(-3.94, 4.33)
Sudomotor function Left Hand (μS): Day 35	55.83	65.47	9.64	(0.36, 18.92)	(-0.76, 20.04)	(-2.14, 21.41)	(-3.98, 23.25)	(-6.94, 26.21)
Sudomotor function Right Hand (μS): Day 35	55.96	59.94	3.98	(-4.24, 12.20)	(-5.22, 13.18)	(-6.41, 14.37)	(-7.99, 15.95)	(-10.50, 18.46)
Sudomotor function Hands (μS): Day 35	56.69	63.56	6.87	(-1.68, 15.42)	(-2.70, 16.44)	(-3.95, 17.68)	(-5.61, 19.34)	(-8.24, 21.98)
Sudomotor function Left Foot (μS): Day 35	74.99	69.07	-5.91	(-14.19, 2.36)	(-15.17, 3.34)	(-16.37, 4.54)	(-17.97, 6.14)	(-20.51, 8.69)
Sudomotor function Right Foot (μS): Day 35	73.98	70.17	-3.81	(-10.15, 2.53)	(-10.90, 3.28)	(-11.82, 4.20)	(-13.04, 5.42)	(-14.97, 7.36)
Sudomotor function Feet (μS): Day 35	74.99	69.64	-5.34	(-12.46, 1.77)	(-13.31, 2.62)	(-14.34, 3.65)	(-15.71, 5.02)	(-17.89, 7.20)
Sudomotor function Overall (μS): Day 35	65.24	63.96	-1.27	(-6.66, 4.11)	(-7.30, 4.75)	(-8.08, 5.53)	(-9.12, 6.57)	(-10.77, 8.22)
Skin hydration (A.U): Day 35	37.25	43.39	6.14	(0.41, 11.88)	(-0.27, 12.56)	(-1.12, 13.40)	(-2.23, 14.52)	(-4.02, 16.31)
Epidermal thickness (μm): Day 35	63.37	66.06	2.69	(-3.68, 9.05)	(-4.43, 9.80)	(-5.36, 10.73)	(-6.59, 11.96)	(-8.55, 13.92)

Cortisol (mcg/24 hours): Day 35	69.95	66.54	-3.41	(-27.29, 20.47)	(-30.13, 23.31)	(-33.61, 26.79)	(-38.22, 31.40)	(-45.56, 38.74)
Urinary [THF+alloTHF]/THE ratio: Day 35	0.99	0.13	-0.87	(-0.99, -0.75)	(-1.00, -0.73)	(-1.02, -0.72)	(-1.04, -0.69)	(-1.08, -0.66)

**Variable**	**Mean***		**Difference***	**Confidence interval***				
	Placebo n=14	AZD4017 n=14		75%	80%	85%	90%	95%
Wound gap diameter (mm): Day 2	1.51	0.98	-0.52	(-0.82, -0.23)	(-0.85, -0.20)	(-0.89, -0.15)	(-0.95, -0.10)	(-1.04, -0.01)
Wound depth (mm): Day 7	0.60	0.59	-0.01	(-0.11, 0.09)	(-0.12, 0.10)	(-0.13, 0.11)	(-0.15, 0.13)	(-0.18, 0.16)
Wound gap diameter (mm): Day 30	1.35	0.71	-0.64	(-0.99, -0.30)	(-1.03, -0.25)	(-1.08, -0.20)	(-1.15, -0.13)	(-1.26, -0.02)
Wound depth (mm): Day 35	0.58	0.56	-0.03	(-0.11, 0.06)	(-0.12, 0.07)	(-0.14, 0.09)	(-0.15, 0.10)	(-0.18, 0.13)

**Variable**	**Geometric mean***		**Ratio***	**Confidence interval***				
	Placebo n=14	AZD4017 n=14		75%	80%	85%	90%	95%
Hour 3 TEWL (Set 1; Day 0)	34.97	31.10	0.89	(0.79, 1.00)	(0.78, 1.02)	(0.76, 1.04)	(0.74, 1.06)	(0.72, 1.10)
Hour 48 TEWL (Set 1; Day 2)	20.20	17.72	0.88	(0.77, 1.00)	(0.75, 1.02)	(0.74, 1.04)	(0.72, 1.07)	(0.69, 1.12)
Hour 168 TEWL (Set 1; Day 7)	13.23	11.97	0.90	(0.75, 1.08)	(0.74, 1.11)	(0.72, 1.14)	(0.69, 1.18)	(0.66, 1.25)
Hour 3 TEWL (Set 2; Day 28)	23.09	30.68	1.33	(1.09, 1.63)	(1.06, 1.66)	(1.03, 1.71)	(0.99, 1.78)	(0.93, 1.89)
Hour 48 TEWL (Set 2; Day 30)	14.65	18.35	1.25	(1.02, 1.53)	(1.00, 1.57)	(0.97, 1.61)	(0.94, 1.68)	(0.88, 1.78)
Hour 168 TEWL (Set 2; Day 35)	9.85	10.70	1.09	(0.88, 1.34)	(0.86, 1.37)	(0.83, 1.42)	(0.80, 1.47)	(0.75, 1.57)
Hour 0 TEWL (Set 3; Day 35)	6.60	9.18	1.39	(1.13, 1.71)	(1.11, 1.75)	(1.07, 1.80)	(1.03, 1.88)	(0.97, 2.00)
Number of tapes required for barrier disruption: Day 28	38.98	55.79	1.43	(1.18, 1.74)	(1.15, 1.78)	(1.12, 1.84)	(1.08, 1.91)	(1.01, 2.02)

*Estimated in imputed data, adjusted for baseline value, if applicable, and for age, sex, and baseline HbA1c.

Table S6: Unadjusted differences in changes from baseline in primary and secondary efficacy outcomes in the full analysis set

Multiple imputation was used to address missing data. For TEWL, integrity, wound depth and diameter, linear regression was used to estimate CIs around differences between the groups. TEWL and integrity measurements were log-transformed before analysis; for these variables changes from baseline are expressed as ratios of geometric means at follow-up to baseline and differences are expressed as ratios of geometric means between groups (AZD4017:placebo). For the remaining variables, which did not meet assumptions for linear regression, median regression was used.

Variable	Median*		Difference*	Confidence interval*				
	Placebo n=14	AZD4017 n=14		75%	80%	85%	90%	95%
11bHSD1 activity radioassay (percent conversion per 24 hours): Day 28	-3.10	-0.50	2.60	(-1.63, 6.83)	(-2.13, 7.33)	(-2.74, 7.94)	(-3.54, 8.74)	(-4.81, 10.01)
11bHSD1 activity ELISA (percent conversion per 24 hours): Day 28	-3.11	-1.80	1.31	(-2.68, 5.29)	(-3.15, 5.76)	(-3.73, 6.34)	(-4.48, 7.09)	(-5.68, 8.29)
Sudomotor function Left Hand (μS): Day 35	-0.30	1.70	2.00	(-7.20, 11.20)	(-8.28, 12.28)	(-9.60, 13.60)	(-11.35, 15.35)	(-14.11, 18.11)
Sudomotor function Right Hand (μS): Day 35	3.65	5.05	1.40	(-6.70, 9.50)	(-7.66, 10.46)	(-8.83, 11.63)	(-10.37, 13.17)	(-12.81, 15.61)
Sudomotor function Hands (μS): Day 35	0.45	4.03	3.58	(-4.65, 11.80)	(-5.62, 12.77)	(-6.81, 13.96)	(-8.38, 15.53)	(-10.86, 18.01)
Sudomotor function Left Foot (μS): Day 35	5.75	-4.00	-9.75	(-17.14, -2.36)	(-18.01, -1.49)	(-19.07, -0.43)	(-20.48, 0.98)	(-22.70, 3.20)
Sudomotor function Right Foot (μS): Day 35	6.80	-1.80	-8.60	(-14.55, -2.65)	(-15.26, -1.94)	(-16.11, -1.09)	(-17.24, 0.04)	(-19.03, 1.83)
Sudomotor function Feet (μS): Day 35	5.90	-3.45	-9.35	(-15.79, -2.91)	(-16.55, -2.15)	(-17.48, -1.22)	(-18.70, 0.00)	(-20.63, 1.93)
Sudomotor function Overall (μS): Day 35	4.71	0.93	-3.79	(-8.68, 1.10)	(-9.26, 1.68)	(-9.97, 2.40)	(-10.92, 3.34)	(-12.42, 4.84)

Skin hydration (A.U): Day 35	-2.88	3.06	5.94	(0.67, 11.21)	(0.05, 11.83)	(-0.71, 12.59)	(-1.71, 13.59)	(-3.29, 15.17)
Epidermal thickness (µm): Day 35	-0.43	-1.92	-1.49	(-11.12, 8.15)	(-12.25, 9.28)	(-13.64, 10.66)	(-15.46, 12.49)	(-18.35, 15.38)
Cortisol (mcg/24 hours): Day 35	-2.70	-1.00	1.70	(-31.61, 35.01)	(-35.54, 38.94)	(-40.33, 43.73)	(-46.66, 50.06)	(-56.65, 60.05)
Urinary [THF+alloTHF]/THE ratio: Day 35	0.01	-0.84	-0.85	(-0.94, -0.75)	(-0.95, -0.74)	(-0.96, -0.73)	(-0.98, -0.71)	(-1.01, -0.68)

**Variable**	**Ratio FU:BL***		**Ratio***	**Confidence interval***				
	Placebo n=14	AZD4017 n=14		75%	80%	85%	90%	95%
Hour 3 TEWL (Set 1; Day 0)	4.24	3.49	0.82	(0.69, 0.98)	(0.68, 1.00)	(0.66, 1.02)	(0.64, 1.06)	(0.61, 1.12)
Hour 48 TEWL (Set 1; Day 2)	2.40	2.33	0.97	(0.80, 1.18)	(0.78, 1.21)	(0.75, 1.25)	(0.73, 1.29)	(0.68, 1.37)
Hour 168 TEWL (Set 1; Day 7)	1.64	1.78	1.09	(0.85, 1.39)	(0.82, 1.43)	(0.80, 1.48)	(0.76, 1.56)	(0.70, 1.68)
Hour 3 TEWL (Set 2; Day 28)	2.85	3.25	1.14	(0.89, 1.45)	(0.87, 1.49)	(0.84, 1.55)	(0.80, 1.62)	(0.74, 1.74)
Hour 48 TEWL (Set 2; Day 30)	1.78	1.98	1.12	(0.89, 1.40)	(0.87, 1.44)	(0.84, 1.48)	(0.80, 1.55)	(0.75, 1.66)
Hour 168 TEWL (Set 2; Day 35)	1.21	1.19	0.98	(0.78, 1.23)	(0.76, 1.26)	(0.73, 1.31)	(0.70, 1.37)	(0.66, 1.46)
Hour 0 TEWL (Set 3; Day 35)	0.86	0.98	1.14	(0.86, 1.51)	(0.83, 1.56)	(0.80, 1.62)	(0.76, 1.71)	(0.70, 1.86)
Number of tapes required for barrier disruption: Day 28	0.87	1.09	1.26	(1.01, 1.58)	(0.98, 1.63)	(0.95, 1.68)	(0.91, 1.75)	(0.85, 1.88)

*Estimated in imputed data.

BL, baseline; FU, follow-up.

Table S7: Adjusted differences in changes from baseline in primary and secondary efficacy outcomes in the full analysis set

Multiple imputation was used to address missing data. For TEWL, integrity, wound depth and diameter, linear regression was used to estimate CIs around differences between the groups. TEWL and integrity measurements were log-transformed before analysis; for these variables changes from baseline are expressed as ratios of geometric means at follow-up to baseline and differences are expressed as ratios of geometric means between groups (AZD4017:placebo). For the remaining variables, which did not meet assumptions for linear regression, median regression was used. The comparison was adjusted for each variable's baseline value (this was not available for wound diameter and depth), age, sex and baseline HbA1c. All TEWL readings were adjusted using pre-disruption TEWL at baseline.

**Variable**	**Median***		**Difference***	**Confidence interval***				
	Placebo n=14	AZD4017 n=14		75%	80%	85%	90%	95%
11bHSD1 activity radioassay (percent conversion per 24 hours): Day 28	-2.99	-1.94	1.05	(-1.98, 4.09)	(-2.34, 4.45)	(-2.78, 4.89)	(-3.37, 5.47)	(-4.30, 6.41)
11bHSD1 activity ELISA (percent conversion per 24 hours): Day 28	-3.93	-4.47	-0.54	(-2.78, 1.70)	(-3.05, 1.97)	(-3.38, 2.30)	(-3.82, 2.74)	(-4.53, 3.45)
Sudomotor function Left Hand (µS): Day 35	-0.01	10.38	10.39	(2.03, 18.74)	(1.04, 19.73)	(-0.18, 20.95)	(-1.80, 22.57)	(-4.38, 25.15)
Sudomotor function Right Hand (µS): Day 35	2.77	7.77	5.01	(-3.07, 13.08)	(-4.03, 14.04)	(-5.20, 15.21)	(-6.75, 16.76)	(-9.21, 19.23)
Sudomotor function Hands (µS): Day 35	1.71	8.96	7.25	(-1.05, 15.56)	(-2.04, 16.54)	(-3.24, 17.75)	(-4.84, 19.35)	(-7.39, 21.89)
Sudomotor function Left Foot (µS): Day 35	6.91	-0.10	-7.01	(-14.81, 0.79)	(-15.73, 1.71)	(-16.86, 2.84)	(-18.36, 4.34)	(-20.73, 6.71)
Sudomotor function Right Foot (µS): Day 35	5.49	1.43	-4.07	(-10.35, 2.21)	(-11.09, 2.96)	(-12.00, 3.87)	(-13.21, 5.07)	(-15.12, 6.99)
Sudomotor function Feet (µS): Day 35	5.77	1.24	-4.52	(-11.36, 2.32)	(-12.18, 3.13)	(-13.17, 4.12)	(-14.48, 5.43)	(-16.56, 7.51)
Sudomotor function Overall (µS): Day 35	3.99	2.66	-1.33	(-6.76, 4.10)	(-7.41, 4.75)	(-8.20, 5.54)	(-9.25, 6.59)	(-10.92, 8.25)

Skin hydration (A.U): Day 35	-2.85	2.82	5.67	(-0.08, 11.43)	(-0.77, 12.12)	(-1.62, 12.97)	(-2.75, 14.10)	(-4.57, 15.92)
Epidermal thickness (µm): Day 35	-2.38	3.20	5.58	(-1.54, 12.69)	(-2.40, 13.55)	(-3.45, 14.60)	(-4.85, 16.01)	(-7.11, 18.26)
Cortisol (mcg/24 hours): Day 35	-14.42	-9.12	5.29	(-19.05, 29.64)	(-21.94, 32.53)	(-25.47, 36.06)	(-30.15, 40.74)	(-37.58, 48.16)
Urinary [THF+alloTHF]/THE ratio: Day 35	0.01	-0.85	-0.87	(-0.98, -0.75)	(-1.00, -0.73)	(-1.01, -0.72)	(-1.04, -0.69)	(-1.07, -0.66)
**Variable**	**Ratio FU:BL***		**Ratio***	**Confidence interval***				
	Placebo n=14	AZD4017 n=14		75%	80%	85%	90%	95%
Hour 3 TEWL (Set 1; Day 0)	4.03	3.68	0.91	(0.81, 1.03)	(0.79, 1.05)	(0.78, 1.07)	(0.76, 1.09)	(0.73, 1.13)
Hour 48 TEWL (Set 1; Day 2)	2.35	2.37	1.01	(0.86, 1.19)	(0.84, 1.21)	(0.82, 1.24)	(0.79, 1.28)	(0.76, 1.34)
Hour 168 TEWL (Set 1; Day 7)	1.62	1.80	1.11	(0.86, 1.44)	(0.83, 1.49)	(0.80, 1.54)	(0.76, 1.62)	(0.70, 1.76)
Hour 3 TEWL (Set 2; Day 28)	2.65	3.53	1.33	(1.09, 1.63)	(1.06, 1.67)	(1.03, 1.72)	(0.99, 1.79)	(0.93, 1.90)
Hour 48 TEWL (Set 2; Day 30)	1.68	2.11	1.26	(1.03, 1.53)	(1.01, 1.56)	(0.98, 1.61)	(0.94, 1.67)	(0.89, 1.77)
Hour 168 TEWL (Set 2; Day 35)	1.16	1.24	1.07	(0.87, 1.31)	(0.85, 1.34)	(0.82, 1.38)	(0.79, 1.44)	(0.74, 1.53)
Hour 0 TEWL (Set 3; Day 35)	0.80	1.06	1.33	(1.07, 1.65)	(1.04, 1.69)	(1.01, 1.75)	(0.97, 1.82)	(0.91, 1.95)
Number of tapes required for barrier disruption: Day 28	0.82	1.17	1.43	(1.18, 1.74)	(1.15, 1.78)	(1.12, 1.84)	(1.07, 1.91)	(1.01, 2.02)

*Estimated in imputed data, adjusted for baseline value, age, sex, and baseline HbA1c.

BL, baseline; FU, follow-up.

Table S8: Sensitivity analysis of TEWL that adjusts for the exact timing of post-disruption measurements in the full analysis set

Analysis models included exact measurement times to account for variations in timings; results have been provided for a) data imputed including all observed values and b) data re-imputed after deleting TEWL readings that were potentially unreliable due to high temperatures.

Linear mixed models were used to model post-baseline TEWL readings (within each set of post-disruption measurements) as a function of time since disruption. Quadratic functions were added to allow for nonlinear change over time. Baseline values and changes over time were permitted to vary between participants. TEWL measurements were log-transformed prior to analysis; differences have been expressed as ratios of geometric means (AZD4017:placebo). For each variable, the comparison was adjusted for baseline (day 0, hour 0) TEWL, age, sex and baseline HbA1c.

**Dataset**	**Disruption day**	**Post-disruption hour**	**Ratio**	**Confidence interval**				
				75%	80%	85%	90%	95%
Imputed	0	3	0.89	(0.78, 1.00)	(0.77, 1.02)	(0.76, 1.03)	(0.74, 1.06)	(0.72, 1.09)
Imputed	0	48	1.00	(0.89, 1.14)	(0.88, 1.15)	(0.86, 1.17)	(0.84, 1.20)	(0.81, 1.24)
Imputed	0	168	1.17	(0.94, 1.45)	(0.91, 1.49)	(0.89, 1.53)	(0.85, 1.59)	(0.80, 1.69)
Imputed	28	3	1.28	(1.08, 1.52)	(1.06, 1.55)	(1.04, 1.59)	(1.01, 1.64)	(0.96, 1.72)
Imputed	28	48	1.25	(1.07, 1.47)	(1.05, 1.50)	(1.03, 1.53)	(1.00, 1.57)	(0.96, 1.64)
Imputed	28	168	1.12	(0.93, 1.34)	(0.92, 1.36)	(0.89, 1.40)	(0.86, 1.44)	(0.82, 1.52)
Re-imputed	0	3	0.88	(0.78, 1.00)	(0.77, 1.01)	(0.76, 1.03)	(0.74, 1.05)	(0.72, 1.08)
Re-imputed	0	48	0.90	(0.80, 1.01)	(0.79, 1.03)	(0.78, 1.04)	(0.76, 1.07)	(0.74, 1.10)
Re-imputed	0	168	0.90	(0.77, 1.04)	(0.76, 1.06)	(0.74, 1.08)	(0.72, 1.11)	(0.69, 1.16)
Re-imputed	28	3	1.28	(1.08, 1.51)	(1.06, 1.54)	(1.03, 1.58)	(1.00, 1.63)	(0.95, 1.71)

**Dataset**	**Disruption day**	**Post-disruption hour**	**Ratio**	**Confidence interval**				
				75%	80%	85%	90%	95%
Re-imputed	28	48	1.25	(1.07, 1.47)	(1.05, 1.50)	(1.02, 1.53)	(1.00, 1.58)	(0.95, 1.65)
Re-imputed	28	168	1.11	(0.93, 1.33)	(0.91, 1.36)	(0.89, 1.39)	(0.86, 1.44)	(0.82, 1.51)

Table S9: Sensitivity analysis of primary and secondary efficacy outcomes in available case data in the full analysis set

For TEWL, integrity, wound depth and diameter, linear regression was used to estimate CIs around differences between the groups. TEWL and integrity measurements were log-transformed before analysis; differences are expressed as ratios of geometric means (AZD4017:placebo). For the remaining variables, which did not meet assumptions for linear regression, median regression was used. The comparison was adjusted for each variable's baseline value (this was not available for wound diameter and depth), age, sex and baseline HbA1c. All TEWL readings were adjusted using pre-disruption TEWL at baseline.

**Variable**	**Estimated median***		**Difference***	**Confidence interval***				
	Placebo	AZD4017		75%	80%	85%	90%	95%
11bHSD1 activity radioassay (percent conversion per 24 hours): Day 28	11.29 (n=13)	12.33 (n=14)	1.03	(-1.64, 3.71)	(-1.84, 3.91)	(-3.11, 5.17)	(-3.38, 5.44)	(-4.11, 6.18)
11bHSD1 activity ELISA (percent conversion per 24 hours): Day 28	5.83 (n=13)	4.44 (n=14)	-1.39	(-3.54, 0.75)	(-3.63, 0.85)	(-3.74, 0.95)	(-4.52, 1.73)	(-5.22, 2.43)
Sudomotor function Left Hand (μS): Day 35	55.60 (n=13)	65.98 (n=13)	10.39	(2.28, 18.50)	(1.66, 19.12)	(0.06, 20.72)	(-1.42, 22.19)	(-3.73, 24.51)
Sudomotor function Right Hand (μS): Day 35	55.71 (n=13)	59.12 (n=13)	3.41	(-5.25, 12.07)	(-5.60, 12.42)	(-7.50, 14.32)	(-8.09, 14.91)	(-9.75, 16.57)
Sudomotor function Hands (μS): Day 35	55.06 (n=13)	57.98 (n=13)	2.92	(-4.53, 10.37)	(-5.43, 11.27)	(-6.78, 12.62)	(-8.36, 14.20)	(-9.22, 15.05)
Sudomotor function Left Foot (μS): Day 35	75.91 (n=13)	68.68 (n=13)	-7.23	(-12.33, -2.14)	(-12.54, -1.93)	(-12.78, -1.69)	(-18.65, 4.18)	(-19.52, 5.05)
Sudomotor function Right Foot (μS): Day 35	72.69 (n=13)	67.83 (n=13)	-4.85	(-10.63, 0.92)	(-10.86, 1.16)	(-11.77, 2.07)	(-12.15, 2.44)	(-16.40, 6.70)
Sudomotor function Feet (μS): Day 35	74.14 (n=13)	69.14 (n=13)	-5.00	(-9.45, -0.55)	(-10.47, 0.47)	(-13.18, 3.18)	(-13.77, 3.78)	(-16.92, 6.93)
Sudomotor function Overall (μS): Day 35	64.51 (n=13)	64.64 (n=13)	0.13	(-5.02, 5.28)	(-7.17, 7.43)	(-7.54, 7.79)	(-7.95, 8.21)	(-9.27, 9.52)
Skin hydration (A.U): Day 35	38.89 (n=12)	43.30 (n=12)	4.41	(-0.99, 9.80)	(-1.21, 10.02)	(-1.92, 10.73)	(-3.40, 12.21)	(-5.83, 14.64)
Epidermal thickness (μm): Day 35	61.95 (n=13)	65.53 (n=12)	3.58	(-3.77, 10.93)	(-4.08, 11.23)	(-4.62, 11.78)	(-5.25, 12.41)	(-6.00, 13.15)

Cortisol (mcg/24 hours): Day 35	60.40 (n=13)	69.11 (n=13)	8.71	(-19.06, 36.48)	(-20.20, 37.61)	(-21.78, 39.19)	(-24.23, 41.65)	(-33.39, 50.80)
Urinary [THF+alloTHF]/THE ratio: Day 35	0.99 (n=13)	0.17 (n=13)	-0.82	(-0.97, -0.67)	(-0.98, -0.66)	(-0.99, -0.65)	(-1.01, -0.63)	(-1.02, -0.62)
	**Estimated mean***		**Difference***			**Confidence interval***		
	Placebo	AZD4017		75%	80%	85%	90%	95%
Wound gap diameter (mm): Day 2	1.51 (n=14)	0.98 (n=14)	-0.52	(-0.82, -0.23)	(-0.85, -0.20)	(-0.89, -0.15)	(-0.95, -0.10)	(-1.04, -0.01)
Wound depth (mm): Day 7	0.60 (n=14)	0.59 (n=14)	-0.01	(-0.10, 0.09)	(-0.11, 0.10)	(-0.13, 0.11)	(-0.14, 0.13)	(-0.17, 0.16)
Wound gap diameter (mm): Day 30	1.35 (n=11)	0.69 (n=12)	-0.66	(-1.00, -0.31)	(-1.05, -0.27)	(-1.10, -0.22)	(-1.16, -0.15)	(-1.27, -0.05)
Wound depth (mm): Day 35	0.59 (n=13)	0.56 (n=12)	-0.02	(-0.11, 0.07)	(-0.12, 0.08)	(-0.14, 0.09)	(-0.15, 0.11)	(-0.18, 0.13)
	**Estimated geometric mean***		**Ratio***			**Confidence interval***		
	Placebo	AZD4017		75%	80%	85%	90%	95%
Hour 3 TEWL (Set 1; Day 0)	34.95 (n=14)	31.26 (n=12)	0.89	(0.79, 1.01)	(0.78, 1.03)	(0.76, 1.05)	(0.74, 1.07)	(0.72, 1.12)
Hour 48 TEWL (Set 1; Day 2)	20.37 (n=14)	20.13 (n=11)	0.99	(0.84, 1.17)	(0.82, 1.19)	(0.80, 1.22)	(0.77, 1.26)	(0.74, 1.33)
Hour 168 TEWL (Set 1; Day 7)	14.23 (n=13)	15.25 (n=13)	1.07	(0.82, 1.40)	(0.79, 1.44)	(0.76, 1.50)	(0.73, 1.58)	(0.67, 1.71)
Hour 3 TEWL (Set 2; Day 28)	23.47 (n=13)	30.51 (n=13)	1.30	(1.06, 1.60)	(1.03, 1.64)	(1.00, 1.69)	(0.96, 1.76)	(0.90, 1.87)
Hour 48 TEWL (Set 2; Day 30)	14.44 (n=13)	18.28 (n=13)	1.27	(1.03, 1.56)	(1.00, 1.60)	(0.97, 1.64)	(0.94, 1.71)	(0.88, 1.82)
Hour 168 TEWL (Set 2; Day 35)	10.11 (n=13)	10.88 (n=12)	1.08	(0.87, 1.33)	(0.85, 1.37)	(0.82, 1.41)	(0.79, 1.47)	(0.74, 1.57)
Hour 0 TEWL (Set 3; Day 35)	6.88 (n=13)	9.02 (n=12)	1.31	(1.05, 1.65)	(1.02, 1.69)	(0.98, 1.75)	(0.94, 1.83)	(0.88, 1.96)
Number of tapes required for barrier disruption: Day 28	38.37 (n=13)	55.37 (n=13)	1.44	(1.17, 1.78)	(1.14, 1.82)	(1.11, 1.88)	(1.07, 1.95)	(1.00, 2.08)

*Adjusted for baseline value if applicable, age, sex, and baseline HbA1c.

Table S10: Sensitivity analysis of primary and secondary efficacy outcomes using last observation carried forward in the full analysis set

For TEWL, integrity, wound depth and diameter, linear regression was used to estimate CIs around differences between the groups. TEWL and integrity measurements were log-transformed before analysis; for these variables changes from baseline are expressed as ratios of geometric means at follow-up to baseline and differences are expressed as ratios of geometric means between groups (AZD4017:placebo). For the remaining variables, which did not meet assumptions for linear regression, median regression was used. The comparison was adjusted for each variable's baseline value (this was not available for wound diameter and depth), age, sex and baseline HbA1c. All TEWL readings were adjusted using pre-disruption TEWL at baseline. TEWL was not measured in 1 participant at baseline as a result of an error and data could therefore only be carried forward for 27 participants.

**Variable**	**Estimated median***		**Difference***	**Confidence interval***				
	Placebo	AZD4017		75%	80%	85%	90%	95%
11bHSD1 activity radioassay (percent conversion per 24 hours): Day 28	13.41 (n=14)	12.69 (n=14)	-0.72	(-3.71, 2.27)	(-3.83, 2.39)	(-4.59, 3.15)	(-5.13, 3.68)	(-5.73, 4.29)
11bHSD1 activity ELISA (percent conversion per 24 hours): Day 28	5.76 (n=14)	4.36 (n=14)	-1.39	(-3.65, 0.87)	(-3.76, 0.97)	(-3.86, 1.07)	(-4.39, 1.60)	(-4.79, 2.00)
Sudomotor function Left Hand (μS): Day 35	56.92 (n=14)	59.57 (n=14)	2.65	(-5.65, 10.96)	(-5.99, 11.29)	(-6.38, 11.68)	(-8.80, 14.10)	(-10.68, 15.98)
Sudomotor function Right Hand (μS): Day 35	54.60 (n=14)	59.45 (n=14)	4.85	(-1.92, 11.62)	(-3.00, 12.71)	(-3.35, 13.06)	(-6.44, 16.14)	(-8.12, 17.83)
Sudomotor function Hands (μS): Day 35	55.06 (n=14)	57.94 (n=14)	2.88	(-3.84, 9.60)	(-4.12, 9.89)	(-4.64, 10.40)	(-8.55, 14.31)	(-9.79, 15.55)
Sudomotor function Left Foot (μS): Day 35	75.20 (n=14)	69.34 (n=14)	-5.86	(-10.67, -1.06)	(-10.87, -0.86)	(-11.22, -0.51)	(-11.69, -0.04)	(-19.25, 7.53)
Sudomotor function Right Foot (μS): Day 35	73.12 (n=14)	69.90 (n=14)	-3.23	(-6.96, 0.51)	(-7.41, 0.95)	(-8.42, 1.97)	(-10.27, 3.82)	(-13.35, 6.89)
Sudomotor function Feet (μS): Day 35	74.01 (n=14)	69.70 (n=14)	-4.31	(-8.44, -0.18)	(-8.61, -0.01)	(-9.63, 1.01)	(-11.71, 3.10)	(-15.60, 6.98)

Sudomotor function Overall (µS): Day 35	64.24 (n=14)	63.79 (n=14)	-0.45	(-4.23, 3.32)	(-5.09, 4.19)	(-7.76, 6.86)	(-8.73, 7.83)	(-9.40, 8.50)
Skin hydration (A.U): Day 35	38.34 (n=14)	43.42 (n=14)	5.08	(1.95, 8.21)	(0.66, 9.50)	(0.46, 9.70)	(-1.79, 11.94)	(-2.86, 13.02)
Epidermal thickness (µm): Day 35	63.31 (n=14)	65.43 (n=14)	2.11	(-4.24, 8.46)	(-4.64, 8.87)	(-5.63, 9.86)	(-6.09, 10.32)	(-6.88, 11.11)
Cortisol (mcg/24 hours): Day 35	64.34 (n=14)	68.42 (n=14)	4.08	(-18.81, 26.97)	(-23.30, 31.47)	(-26.68, 34.84)	(-28.32, 36.48)	(-37.79, 45.95)
Urinary [THF+alloTHF]/THE ratio: Day 35	0.99 (n=14)	0.17 (n=14)	-0.82	(-0.96, -0.68)	(-0.96, -0.68)	(-1.01, -0.63)	(-1.02, -0.62)	(-1.04, -0.60)
	**Estimated mean***		**Difference***			**Confidence interval***		
	Placebo	AZD4017		75%	80%	85%	90%	95%
Wound gap diameter (mm): Day 2	1.51 (n=14)	0.98 (n=14)	-0.52	(-0.82, -0.23)	(-0.85, -0.20)	(-0.89, -0.15)	(-0.95, -0.10)	(-1.04, -0.01)
Wound depth (mm): Day 7	0.60 (n=14)	0.59 (n=14)	-0.01	(-0.10, 0.09)	(-0.11, 0.10)	(-0.13, 0.11)	(-0.14, 0.13)	(-0.17, 0.16)
Wound gap diameter (mm): Day 30	1.32 (n=14)	0.66 (n=14)	-0.66	(-0.94, -0.37)	(-0.98, -0.34)	(-1.02, -0.30)	(-1.07, -0.24)	(-1.16, -0.16)
Wound depth (mm): Day 35	0.59 (n=14)	0.56 (n=14)	-0.03	(-0.11, 0.05)	(-0.12, 0.06)	(-0.13, 0.07)	(-0.15, 0.09)	(-0.17, 0.11)
	**Estimated geometric mean***		**Ratio***			**Confidence interval***		
	Placebo	AZD4017		75%	80%	85%	90%	95%
Hour 3 TEWL (Set 1; Day 0)	34.85 (n=14)	29.97 (n=13)	0.86	(0.75, 0.98)	(0.74, 1.00)	(0.73, 1.02)	(0.71, 1.04)	(0.68, 1.08)
Hour 48 TEWL (Set 1; Day 2)	20.37 (n=14)	21.11 (n=13)	1.04	(0.89, 1.21)	(0.87, 1.23)	(0.85, 1.26)	(0.83, 1.30)	(0.79, 1.36)
Hour 168 TEWL (Set 1; Day 7)	14.59 (n=14)	15.22 (n=13)	1.04	(0.81, 1.35)	(0.78, 1.39)	(0.75, 1.44)	(0.72, 1.52)	(0.66, 1.64)
Hour 3 TEWL (Set 2; Day 28)	21.70 (n=14)	29.62 (n=13)	1.36	(1.08, 1.73)	(1.05, 1.78)	(1.01, 1.84)	(0.97, 1.93)	(0.90, 2.07)
Hour 48 TEWL (Set 2; Day 30)	13.87 (n=14)	18.00 (n=13)	1.30	(1.05, 1.60)	(1.03, 1.64)	(1.00, 1.69)	(0.96, 1.76)	(0.90, 1.88)
Hour 168 TEWL (Set 2; Day 35)	9.92 (n=14)	11.87 (n=13)	1.20	(0.95, 1.51)	(0.92, 1.56)	(0.89, 1.61)	(0.85, 1.68)	(0.79, 1.81)
Hour 0 TEWL (Set 3; Day 35)	6.88 (n=14)	9.67 (n=13)	1.41	(1.12, 1.77)	(1.09, 1.81)	(1.05, 1.88)	(1.01, 1.96)	(0.94, 2.10)
Number of tapes required for barrier disruption: Day 28	39.36 (n=14)	55.58 (n=13)	1.41	(1.15, 1.73)	(1.13, 1.77)	(1.09, 1.82)	(1.05, 1.90)	(0.99, 2.02)

*Adjusted for baseline value, if applicable, for age, sex, and baseline HbA1c.

Table S11: Correlations between AZD4017 compliance and efficacy outcomes in the full analysis set (AZD4017 group only)

Absolute rho values of at least 0.3 were considered preliminary evidence of an association. The sensitivity analysis excluded baseline radioimmunoassay samples that failed QC, an outlying ELISA assay result at day 28, TEWL readings recorded on very hot days, and results that were recorded 1 day earlier than planned, before imputation. Not all of these issues occurred in samples from the AZD4017 group, but issues that did occur could have affected imputed values for any incomplete variables.

	All values		Excluding outliers/QC failures	
**Variable**	**Imputed**	**Available case**	**Imputed**	**Available case**
11bHSD1 activity radioassay (percent conversion per 24 hours): Day 28	-0.04 (14)	-0.04 (14)	-0.04 (14)	-0.04 (14)
11bHSD1 activity ELISA (percent conversion per 24 hours): Day 28	-0.74 (14)	-0.74 (14)	-0.74 (14)	-0.74 (14)
Sudomotor function Left Hand (µS): Day 35	-0.05 (14)	-0.08 (13)	-0.03 (14)	-0.08 (13)
Sudomotor function Right Hand (µS): Day 35	-0.16 (14)	-0.23 (13)	-0.16 (14)	-0.23 (13)
Sudomotor function Hands (µS): Day 35	-0.03 (14)	-0.07 (13)	-0.03 (14)	-0.07 (13)
Sudomotor function Left Foot (µS): Day 35	-0.21 (14)	-0.20 (13)	-0.19 (14)	-0.20 (13)
Sudomotor function Right Foot (µS): Day 35	0.06 (14)	0.12 (13)	0.09 (14)	0.12 (13)
Sudomotor function Feet (µS): Day 35	-0.18 (14)	-0.17 (13)	-0.15 (14)	-0.17 (13)
Sudomotor function Overall (µS): Day 35	0.03 (14)	0.05 (13)	0.05 (14)	0.05 (13)
Skin hydration (A.U): Day 35	0.55 (14)	0.77 (12)	0.62 (14)	0.77 (12)
Epidermal thickness (µm): Day 35	-0.11 (14)	-0.15 (12)	-0.11 (14)	-0.15 (12)
Cortisol (mcg/24 hours): Day 35	-0.30 (14)	-0.31 (13)	-0.31 (14)	-0.31 (13)
Urinary [THF+alloTHF]/THE ratio: Day 35	-0.28 (14)	-0.34 (13)	-0.26 (14)	-0.34 (13)
Wound gap diameter (mm): Day 2	0.35 (14)	0.35 (14)	0.35 (14)	0.35 (14)

Variable	All values		Excluding outliers/QC failures	
	Imputed	Available case	Imputed	Available case
Wound depth (mm): Day 7	0.23 (14)	0.23 (14)	0.27 (14)	0.31 (13)
Wound gap diameter (mm): Day 30	-0.06 (14)	-0.01 (12)	-0.07 (14)	-0.01 (12)
Wound depth (mm): Day 35	0.09 (14)	0.10 (12)	0.09 (14)	0.10 (12)
Hour 0 TEWL (Set 1; Day 0)	-0.42 (14)	-0.43 (13)	-0.43 (14)	-0.43 (13)
Hour 3 TEWL (Set 1; Day 0)	-0.35 (14)	-0.39 (12)	-0.34 (14)	-0.39 (12)
Hour 48 TEWL (Set 1; Day 2)	-0.20 (14)	-0.13 (11)	-0.25 (14)	-0.17 (8)
Hour 168 TEWL (Set 1; Day 7)	-0.07 (14)	-0.01 (13)	0.18 (14)	0.38 (9)
Hour 0 TEWL (Set 2; Day 28)	-0.33 (14)	-0.33 (14)	-0.33 (14)	-0.33 (14)
Hour 3 TEWL (Set 2; Day 28)	-0.37 (14)	-0.37 (14)	-0.37 (14)	-0.37 (14)
Hour 48 TEWL (Set 2; Day 30)	0.29 (14)	0.29 (14)	0.29 (14)	0.29 (14)
Hour 168 TEWL (Set 2; Day 35)	0.37 (14)	0.38 (12)	0.34 (14)	0.38 (12)
Hour 0 TEWL (Set 3; Day 35)	0.30 (14)	0.39 (12)	0.31 (14)	0.39 (12)
Number of tapes required for barrier disruption: Day 28	-0.15 (14)	-0.15 (14)	-0.15 (14)	-0.15 (14)

All values presented as Spearman's rho (number of participants included).

Table S12: Unadjusted differences in final values of longitudinal laboratory safety variables in the safety set

Multiple imputation was used to address missing data; results are presented for placebo n=14, AZD4017 n=14. All point estimates and CIs were estimated using linear regression. For each visit, the values presented are: mean placebo group, mean AZD4017 group; difference AZD4017-placebo (90% CI).

Variable	Day 7	Day 28	Day 35	Day 42
Body mass index (kg/m^2)	N/A	N/A	33.90, 35.55; 1.65 (-5.35, 8.65)	N/A
Waist-hip ratio	N/A	N/A	0.98, 1.02; 0.04 (-0.01, 0.09)	N/A
Systolic blood pressure (mm Hg)	N/A	N/A	143.66, 129.01; -14.64 (-22.83, -6.45)	136.56, 137.50; 0.94 (-7.02, 8.89)
Diastolic blood pressure (mm Hg)	N/A	N/A	79.95, 73.64; -6.31 (-11.24, -1.39)	80.11, 79.29; -0.83 (-8.02, 6.37)
HbA1c (mmol/mol)	71.88, 65.81; -6.07 (-17.14, 5.00)	70.14, 66.00; -4.14 (-14.73, 6.45)	70.64, 64.79; -5.85 (-17.14, 5.44)	69.37, 65.43; -3.94 (-14.93, 7.05)
High-density lipoprotein (mmol/l)	1.16, 1.05; -0.11 (-0.28, 0.07)	1.22, 1.11; -0.11 (-0.28, 0.07)	1.18, 1.01; -0.17 (-0.34, 0.00)	1.19, 1.21; 0.02 (-0.18, 0.22)
Cholesterol (mmol/l)	4.31, 3.59; -0.71 (-1.26, -0.17)	4.21, 3.54; -0.67 (-1.17, -0.16)	4.11, 3.44; -0.68 (-1.18, -0.17)	4.06, 3.84; -0.22 (-0.77, 0.33)
Triglycerides (mmol/l)	2.11, 2.39; 0.28 (-0.51, 1.07)	1.81, 1.71; -0.11 (-0.63, 0.42)	2.22, 2.37; 0.15 (-0.91, 1.21)	1.61, 1.94; 0.33 (-0.39, 1.06)
Hemoglobin (g/l)	136.07, 137.43; 1.36 (-6.38, 9.09)	138.46, 138.36; -0.10 (-6.97, 6.77)	134.84, 136.30; 1.46 (-6.54, 9.46)	136.56, 139.86; 3.29 (-5.51, 12.09)
White blood cells ($x10^9/l$)	6.71, 7.96; 1.26 (-0.02, 2.54)	6.52, 6.72; 0.20 (-0.97, 1.38)	6.68, 6.93; 0.25 (-1.00, 1.50)	6.55, 7.66; 1.12 (-0.23, 2.46)
Platelets ($x10^9/l$)	219.36, 275.29; 55.93 (8.28, 103.58)	219.37, 288.57; 69.20 (15.99, 122.41)	213.32, 265.82; 52.50 (2.98, 102.03)	220.63, 281.43; 60.80 (10.77, 110.82)
Red blood cells ($x10^{12}/l$)	4.74, 4.59; -0.16 (-0.48, 0.16)	4.81, 4.68; -0.13 (-0.41, 0.16)	4.67, 4.57; -0.10 (-0.39, 0.19)	4.73, 4.77; 0.04 (-0.29, 0.38)
Mean corpuscular volume (fl)	89.71, 92.14; 2.43 (-1.26, 6.12)	88.30, 91.21; 2.92 (-1.05, 6.88)	90.23, 92.20; 1.96 (-2.13, 6.06)	88.19, 90.50; 2.31 (-1.16, 5.78)
Hematocrit (packed cell volume)	0.43, 0.42; -0.01 (-0.03, 0.02)	0.42, 0.43; 0.00 (-0.02, 0.03)	0.42, 0.42; 0.00 (-0.03, 0.03)	0.42, 0.43; 0.02 (-0.01, 0.04)
Mean corpuscular hemoglobin (pg)	28.79, 30.13; 1.34 (-0.12, 2.80)	28.96, 29.68; 0.72 (-0.61, 2.05)	28.97, 29.99; 1.02 (-0.44, 2.48)	29.01, 29.42; 0.41 (-1.02, 1.85)
Corpuscular hemoglobin concentration (g/l)	321.07, 326.93; 5.86 (-0.96, 12.68)	328.44, 325.79; -2.66 (-9.90, 4.59)	321.93, 325.20; 3.27 (-3.47, 10.01)	328.81, 324.86; -3.95 (-11.54, 3.64)
Red blood cell distribution width (%)	14.43, 14.27; -0.16 (-0.94, 0.62)	13.98, 14.08; 0.10 (-0.45, 0.65)	14.21, 14.42; 0.21 (-0.38, 0.81)	13.76, 13.74; -0.02 (-0.68, 0.64)

Variable	Day 7	Day 28	Day 35	Day 42
Albumin (g/l)	39.50, 40.14; 0.64 (-1.23, 2.52)	38.94, 38.71; -0.22 (-1.95, 1.51)	39.88, 40.04; 0.16 (-1.65, 1.98)	38.29, 38.84; 0.56 (-0.80, 1.91)
Bilirubin (umol/l)	8.36, 8.64; 0.29 (-2.43, 3.00)	9.81, 9.86; 0.05 (-2.50, 2.60)	8.80, 8.36; -0.44 (-3.41, 2.53)	8.29, 8.40; 0.11 (-1.54, 1.75)
Alkaline phosphatase (U/l)	80.86, 80.14; -0.71 (-17.98, 16.56)	78.01, 68.93; -9.08 (-23.40, 5.24)	77.67, 68.18; -9.50 (-23.41, 4.41)	78.30, 76.94; -1.36 (-19.31, 16.60)
Alanine aminotransferase (iu/l)	22.93, 26.14; 3.21 (-2.22, 8.64)	23.19, 23.07; -0.12 (-5.46, 5.22)	22.81, 22.19; -0.61 (-6.76, 5.53)	21.74, 23.96; 2.23 (-3.55, 8.00)
Aspartate aminotransferase (iu/l)	21.07, 22.36; 1.29 (-1.98, 4.55)	20.63, 21.36; 0.73 (-1.56, 3.01)	20.76, 20.92; 0.16 (-2.17, 2.49)	19.15, 21.80; 2.65 (-0.73, 6.03)
Gamma-glutamyl transpeptidase (iu/l)	39.71, 37.71; -2.00 (-22.51, 18.51)	42.23, 29.86; -12.37 (-29.42, 4.68)	43.29, 28.10; -15.19 (-35.42, 5.05)	40.96, 28.82; -12.14 (-29.49, 5.21)
eGFR (ml/min/1.73m^2)	77.50, 71.50; -6.00 (-14.97, 2.97)	80.49, 75.14; -5.34 (-13.52, 2.83)	76.61, 73.60; -3.02 (-13.42, 7.39)	78.28, 76.21; -2.06 (-10.63, 6.51)
Sodium (mmol/l)	139.29, 140.00; 0.71 (-0.83, 2.26)	138.12, 138.79; 0.66 (-0.62, 1.95)	140.05, 139.28; -0.78 (-2.63, 1.08)	138.86, 138.00; -0.86 (-2.24, 0.52)
Potassium (mmol/l)	4.79, 4.59; -0.20 (-0.50, 0.10)	4.50, 4.74; 0.24 (0.00, 0.49)	4.47, 4.52; 0.05 (-0.18, 0.28)	4.63, 4.63; -0.00 (-0.26, 0.26)
Urea (mmol/l)	6.91, 7.24; 0.33 (-1.18, 1.84)	6.71, 6.56; -0.15 (-2.04, 1.74)	7.68, 7.49; -0.20 (-2.70, 2.31)	7.04, 7.57; 0.53 (-1.33, 2.38)
Creatinine (umol/l)	82.36, 88.57; 6.21 (-6.95, 19.38)	77.83, 80.93; 3.10 (-8.58, 14.77)	84.69, 84.94; 0.25 (-14.58, 15.08)	81.18, 79.93; -1.25 (-12.61, 10.11)
Testosterone (nmol/l)	10.34, 6.43; -3.91 (-8.01, 0.18)	11.08, 6.46; -4.62 (-8.48, -0.75)	10.26, 6.46; -3.80 (-7.92, 0.31)	10.25, 7.66; -2.59 (-6.72, 1.53)
Dehydroepiandrosterone sulphate (umol/l)	3.56, 4.91; 1.36 (-0.58, 3.30)	3.25, 5.35; 2.10 (0.53, 3.66)	3.51, 4.91; 1.39 (-0.31, 3.10)	3.00, 3.55; 0.55 (-0.75, 1.85)
Free thyroxine (pmol/l)	14.63, 15.75; 1.12 (-0.28, 2.52)	14.64, 15.74; 1.10 (-0.05, 2.26)	14.39, 15.48; 1.09 (-0.05, 2.23)	14.00, 15.10; 1.10 (0.15, 2.06)
Thyroid-stimulating hormone (mIU/l)	1.68, 1.84; 0.16 (-0.27, 0.59)	1.89, 1.71; -0.18 (-0.71, 0.35)	1.77, 1.68; -0.09 (-0.54, 0.35)	1.79, 1.76; -0.03 (-0.57, 0.51)

Table S13: Unadjusted differences in changes from baseline in longitudinal laboratory safety variables in the safety set

Multiple imputation was used to address missing data; results are presented for placebo n=14, AZD4017 n=14. All point estimates and CIs were estimated using linear regression. For each visit, the values presented are: mean placebo group, mean AZD4017 group; difference AZD4017-placebo (90% CI).

Variable	Day 7	Day 28	Day 35	Day 42
Body mass index (kg/m^2)	N/A	N/A	0.23, 0.51; 0.28 (-0.64, 1.19)	N/A
Waist-hip ratio	N/A	N/A	-0.00, -0.00; -0.00 (-0.03, 0.03)	N/A
Systolic blood pressure (mm Hg)	N/A	N/A	7.94, -11.41; -19.36 (-31.07, -7.64)	0.85, -2.93; -3.78 (-13.95, 6.39)
Diastolic blood pressure (mm Hg)	N/A	N/A	-3.90, 1.00; 4.90 (-1.16, 10.96)	-3.75, 6.64; 10.39 (2.27, 18.51)
HbA1c (mmol/mol)	-0.40, -0.19; 0.22 (-1.26, 1.69)	-2.15, 0.00; 2.15 (-0.89, 5.18)	-1.64, -1.21; 0.44 (-3.99, 4.86)	-2.92, -0.57; 2.35 (-2.59, 7.28)
High density lipoprotein (mmol/l)	-0.08, -0.14; -0.06 (-0.15, 0.02)	-0.01, -0.08; -0.07 (-0.14, 0.01)	-0.06, -0.19; -0.13 (-0.21, -0.05)	-0.04, 0.02; 0.06 (-0.04, 0.17)
Cholesterol (mmol/l)	-0.05, -0.36; -0.31 (-0.51, -0.10)	-0.15, -0.41; -0.26 (-0.66, 0.14)	-0.24, -0.51; -0.27 (-0.70, 0.17)	-0.29, -0.11; 0.19 (-0.34, 0.71)
Triglycerides (mmol/l)	0.44, 0.37; -0.06 (-0.54, 0.41)	0.13, -0.31; -0.45 (-0.85, -0.05)	0.54, 0.35; -0.19 (-0.89, 0.50)	-0.07, -0.08; -0.01 (-0.50, 0.48)
Hemoglobin (g/l)	-3.14, -2.00; 1.14 (-2.06, 4.34)	-0.76, -1.07; -0.31 (-4.32, 3.69)	-4.38, -3.13; 1.24 (-3.57, 6.06)	-2.65, 0.43; 3.08 (-0.70, 6.86)
White blood cells (x10^9/l)	0.25, 0.63; 0.37 (-0.33, 1.08)	0.06, -0.62; -0.68 (-1.27, -0.09)	0.22, -0.41; -0.63 (-1.40, 0.13)	0.09, 0.33; 0.23 (-0.58, 1.05)
Platelets (x10^9/l)	0.64, 2.14; 1.50 (-12.40, 15.40)	0.66, 15.43; 14.77 (-6.85, 36.40)	-5.40, -7.32; -1.93 (-23.02, 19.17)	1.92, 8.29; 6.37 (-10.84, 23.58)
Red blood cells (x10^{12}/l)	-0.10, -0.13; -0.03 (-0.13, 0.07)	-0.03, -0.03; 0.00 (-0.11, 0.11)	-0.17, -0.14; 0.03 (-0.11, 0.16)	-0.11, 0.06; 0.17 (0.06, 0.29)
Mean corpuscular volume (fl)	1.29, 0.93; -0.36 (-2.12, 1.41)	-0.13, 0.00; 0.13 (-1.68, 1.94)	1.80, 0.98; -0.82 (-2.99, 1.35)	-0.24, -0.71; -0.48 (-2.01, 1.06)
Hematocrit (packed cell volume)	-0.00, -0.01; -0.01 (-0.02, 0.01)	-0.01, -0.00; 0.01 (-0.01, 0.02)	-0.01, -0.01; 0.00 (-0.01, 0.02)	-0.01, 0.01; 0.02 (0.00, 0.03)
Mean corpuscular hemoglobin (pg)	-0.03, 0.39; 0.42 (0.07, 0.78)	0.14, -0.06; -0.19 (-0.59, 0.20)	0.15, 0.25; 0.10 (-0.38, 0.59)	0.19, -0.31; -0.50 (-0.99, -0.01)
Corpuscular hemoglobin concentration (g/l)	-4.50, 0.29; 4.79 (-1.61, 11.19)	2.87, -0.86; -3.73 (-10.81, 3.35)	-3.64, -1.44; 2.20 (-5.76, 10.16)	3.24, -1.79; -5.03 (-11.66, 1.61)
Red blood cell distribution width (%)	0.55, 0.15; -0.40 (-0.89, 0.09)	0.10, -0.04; -0.14 (-0.62, 0.34)	0.33, 0.30; -0.03 (-0.46, 0.40)	-0.12, -0.38; -0.26 (-0.73, 0.21)

Variable	Day 7	Day 28	Day 35	Day 42
Albumin (g/l)	0.07, 0.64; 0.57 (-0.61, 1.75)	-0.49, -0.79; -0.29 (-1.70, 1.12)	0.45, 0.54; 0.09 (-1.45, 1.63)	-1.14, -0.66; 0.49 (-0.62, 1.59)
Bilirubin (umol/l)	-0.57, 0.50; 1.07 (-0.75, 2.90)	0.88, 1.71; 0.83 (-0.69, 2.35)	-0.13, 0.21; 0.35 (-2.19, 2.88)	-0.64, 0.26; 0.89 (-0.85, 2.64)
Alkaline phosphatase (U/l)	0.43, -4.93; -5.36 (-11.92, 1.20)	-2.42, -16.14; -13.72 (-22.00, -5.44)	-2.75, -16.89; -14.14 (-21.88, -6.39)	-2.13, -8.13; -6.00 (-16.68, 4.68)
Alanine aminotransferase (iu/l)	-1.71, -1.29; 0.43 (-2.53, 3.38)	-1.45, -4.36; -2.90 (-6.02, 0.21)	-1.84, -5.24; -3.40 (-8.09, 1.29)	-2.91, -3.47; -0.56 (-4.51, 3.39)
Aspartate aminotransferase (iu/l)	0.07, -0.29; -0.36 (-3.22, 2.51)	-0.37, -1.29; -0.92 (-3.59, 1.75)	-0.24, -1.73; -1.48 (-4.90, 1.94)	-1.85, -0.85; 1.01 (-2.75, 4.76)
Gamma-glutamyl transpeptidase (iu/l)	-2.14, -1.57; 0.57 (-2.73, 3.87)	0.37, -9.43; -9.80 (-17.09, -2.51)	1.43, -11.18; -12.61 (-25.48, 0.26)	-0.90, -10.47; -9.57 (-17.50, -1.64)
eGFR (ml/min/1.73m^2)	-4.36, -7.71; -3.36 (-7.87, 1.15)	-1.37, -4.07; -2.70 (-5.83, 0.43)	-5.24, -5.62; -0.38 (-5.36, 4.61)	-3.58, -3.00; 0.58 (-3.91, 5.08)
Sodium (mmol/l)	0.36, -0.71; -1.07 (-3.83, 1.69)	-0.81, -1.93; -1.12 (-3.94, 1.70)	1.13, -1.44; -2.56 (-5.46, 0.33)	-0.07, -2.71; -2.65 (-5.44, 0.15)
Potassium (mmol/l)	0.33, -0.04; -0.36 (-0.62, -0.11)	0.03, 0.11; 0.08 (-0.16, 0.32)	0.01, -0.11; -0.11 (-0.37, 0.14)	0.17, 0.01; -0.16 (-0.43, 0.10)
Urea (mmol/l)	-0.04, -0.01; 0.04 (-0.87, 0.94)	-0.25, -0.69; -0.45 (-1.34, 0.45)	0.72, 0.24; -0.49 (-1.84, 0.87)	0.09, 0.32; 0.23 (-0.82, 1.29)
Creatinine (umol/l)	5.71, 12.36; 6.64 (0.17, 13.12)	1.19, 4.71; 3.53 (-0.64, 7.69)	8.05, 8.72; 0.68 (-6.83, 8.18)	4.54, 3.71; -0.82 (-7.40, 5.75)
Testosterone (nmol/l)	-0.76, -0.54; 0.21 (-1.52, 1.94)	-0.02, -0.51; -0.49 (-1.83, 0.86)	-0.84, -0.51; 0.33 (-1.61, 2.26)	-0.85, 0.69; 1.53 (-0.41, 3.48)
Dehydroepiandrosterone sulphate (umol/l)	0.03, 2.00; 1.97 (1.04, 2.91)	-0.28, 2.44; 2.71 (1.98, 3.44)	-0.02, 1.99; 2.01 (1.14, 2.88)	-0.53, 0.64; 1.16 (0.51, 1.82)
Free thyroxine (pmol/l)	-0.24, 0.02; 0.26 (-0.89, 1.40)	-0.22, 0.01; 0.24 (-0.76, 1.23)	-0.47, -0.24; 0.23 (-0.94, 1.39)	-0.87, -0.63; 0.24 (-0.67, 1.15)
Thyroid-stimulating hormone (mlU/l)	-0.06, 0.12; 0.18 (-0.17, 0.52)	0.15, -0.01; -0.16 (-0.51, 0.18)	0.03, -0.05; -0.08 (-0.48, 0.33)	0.05, 0.04; -0.01 (-0.40, 0.37)

*Estimated in imputed data.

Table S14: Adjusted differences in changes from baseline in longitudinal laboratory safety variables in the safety set

Multiple imputation was used to address missing data; results are presented for placebo n=14, AZD4017 n=14. All point estimates and CIs were estimated using linear regression, adjusted for the variable's baseline value, age, sex, and baseline HbA1c. For each visit, the values presented are: mean placebo group, mean AZD4017 group; difference AZD4017-placebo (90% CI).

Variable	Day 7	Day 28	Day 35	Day 42
Body mass index (kg/m^2)	N/A	N/A	0.17, 0.58; 0.41 (-0.51, 1.34)	N/A
Waist-hip ratio	N/A	N/A	-0.01, 0.01; 0.02 (-0.01, 0.05)	N/A
Systolic blood pressure (mm Hg)	N/A	N/A	5.61, -9.13; -14.74 (-23.00, -6.47)	89.67, 90.44; 0.77 (-6.58, 8.11)
Diastolic blood pressure (mm Hg)	N/A	N/A	-0.25, -2.69; -2.43 (-7.67, 2.80)	58.31, 60.40; 2.09 (-6.77, 10.95)
HbA1c (mmol/mol)	-0.38, -0.19; 0.18 (-1.51, 1.88)	-2.10, 0.08; 2.17 (-0.64, 4.99)	-1.71, -0.99; 0.72 (-3.64, 5.09)	-2.87, -0.45; 2.42 (-2.38, 7.22)
High-density lipoprotein (mmol/l)	-0.08, -0.14; -0.06 (-0.14, 0.02)	0.18, 0.10; -0.08 (-0.16, 0.00)	0.10, -0.03; -0.13 (-0.21, -0.05)	0.06, 0.11; 0.05 (-0.07, 0.16)
Cholesterol (mmol/l)	0.00, -0.41; -0.42 (-0.60, -0.23)	1.55, 1.10; -0.46 (-0.79, -0.12)	0.97, 0.46; -0.51 (-0.87, -0.15)	1.28, 1.18; -0.10 (-0.54, 0.34)
Triglycerides (mmol/l)	0.47, 0.33; -0.14 (-0.67, 0.40)	0.62, 0.43; -0.19 (-0.56, 0.18)	-0.03, -0.52; -0.48 (-1.21, 0.24)	0.05, 0.07; 0.02 (-0.51, 0.55)
Hemoglobin (g/l)	-2.90, -2.24; 0.67 (-2.12, 3.45)	45.08, 44.10; -0.98 (-4.13, 2.17)	15.89, 16.53; 0.64 (-3.96, 5.24)	-3.55, -1.48; 2.07 (-1.62, 5.76)
White blood cells (x10^9/l)	0.17, 0.71; 0.54 (-0.14, 1.22)	1.76, 1.36; -0.40 (-0.87, 0.08)	1.51, 1.21; -0.31 (-0.96, 0.34)	1.07, 1.40; 0.33 (-0.41, 1.07)
Platelets (x10^9/l)	0.10, 2.52; 2.42 (-13.65, 18.50)	7.72, 21.04; 13.32 (-9.56, 36.20)	9.47, 8.55; -0.91 (-25.20, 23.37)	2.42, 3.89; 1.47 (-16.74, 19.68)
Red blood cells (x10^{12}/l)	-0.09, -0.14; -0.05 (-0.17, 0.06)	0.93, 0.88; -0.05 (-0.15, 0.04)	0.42, 0.40; -0.02 (-0.15, 0.12)	-0.29, -0.15; 0.15 (0.03, 0.26)
Mean corpuscular volume (fl)	1.06, 1.17; 0.11 (-1.83, 2.05)	6.03, 6.55; 0.52 (-1.49, 2.53)	9.58, 9.41; -0.17 (-2.53, 2.19)	12.90, 13.06; 0.16 (-1.42, 1.73)
Hematocrit (packed cell volume)	-0.00, -0.01; -0.01 (-0.02, 0.01)	0.12, 0.12; 0.00 (-0.01, 0.02)	0.03, 0.03; 0.00 (-0.02, 0.02)	-0.02, -0.01; 0.02 (0.00, 0.03)
Mean corpuscular hemoglobin (pg)	-0.02, 0.39; 0.41 (0.08, 0.75)	3.58, 3.51; -0.07 (-0.44, 0.31)	1.34, 1.51; 0.17 (-0.28, 0.62)	2.42, 2.00; -0.42 (-0.95, 0.10)
Corpuscular hemoglobin concentration (g/l)	-4.52, 0.39; 4.91 (-1.41, 11.22)	150.85, 147.06; -3.80 (-10.80, 3.21)	121.63, 123.38; 1.75 (-4.85, 8.35)	62.90, 57.67; -5.22 (-12.18, 1.74)
Red blood cell distribution width (%)	0.52, 0.17; -0.36 (-0.89, 0.18)	6.04, 5.95; -0.09 (-0.49, 0.31)	2.59, 2.70; 0.11 (-0.28, 0.51)	2.84, 2.64; -0.21 (-0.68, 0.26)

Variable	Day 7	Day 28	Day 35	Day 42
Albumin (g/l)	0.18, 0.54; 0.37 (-0.82, 1.55)	10.87, 10.50; -0.37 (-1.82, 1.09)	9.36, 9.35; -0.01 (-1.60, 1.59)	11.80, 12.19; 0.40 (-0.59, 1.38)
Bilirubin (umol/l)	-0.50, 0.43; 0.93 (-1.14, 3.00)	1.36, 1.97; 0.60 (-0.89, 2.09)	4.14, 3.50; -0.64 (-3.01, 1.73)	2.16, 2.50; 0.35 (-1.17, 1.87)
Alkaline phosphatase (U/l)	0.66, -5.34; -6.00 (-12.44, 0.44)	18.33, 5.94; -12.39 (-20.21, -4.56)	12.99, -0.10; -13.09 (-20.43, -5.75)	8.43, 2.78; -5.65 (-15.25, 3.95)
Alanine aminotransferase (iu/l)	-2.09, -0.94; 1.16 (-1.84, 4.15)	4.23, 2.27; -1.96 (-4.94, 1.03)	5.83, 4.19; -1.64 (-5.88, 2.61)	1.56, 2.34; 0.78 (-3.00, 4.56)
Aspartate aminotransferase (iu/l)	-0.33, 0.13; 0.46 (-2.29, 3.21)	11.62, 11.74; 0.12 (-1.81, 2.05)	6.93, 6.90; -0.04 (-2.52, 2.45)	6.53, 8.74; 2.21 (-1.02, 5.44)
Gamma-glutamyl transpeptidase (iu/l)	-2.23, -1.47; 0.76 (-2.83, 4.35)	10.09, -0.19; -10.28 (-14.96, -5.60)	7.96, -4.02; -11.97 (-22.80, -1.15)	6.46, -3.09; -9.55 (-14.88, -4.22)
eGFR (ml/min/1.73m^2)	-4.42, -7.60; -3.18 (-8.30, 1.94)	2.79, 0.60; -2.19 (-5.38, 1.01)	-14.06, -13.79; 0.27 (-4.57, 5.10)	4.24, 5.86; 1.62 (-2.77, 6.01)
Sodium (mmol/l)	-0.23, -0.12; 0.10 (-1.23, 1.44)	138.48, 138.64; 0.16 (-0.98, 1.29)	1.57, 0.37; -1.19 (-3.21, 0.82)	135.61, 134.37; -1.25 (-2.42, -0.07)
Potassium (mmol/l)	0.30, -0.00; -0.31 (-0.58, -0.03)	2.17, 2.35; 0.18 (-0.06, 0.43)	1.61, 1.63; 0.01 (-0.22, 0.25)	1.85, 1.80; -0.04 (-0.31, 0.22)
Urea (mmol/l)	-0.08, 0.04; 0.13 (-0.79, 1.05)	-0.33, -0.68; -0.35 (-1.31, 0.61)	-1.47, -1.99; -0.52 (-1.88, 0.83)	0.16, 0.18; 0.02 (-1.04, 1.08)
Creatinine (umol/l)	5.30, 12.74; 7.43 (0.79, 14.08)	-1.29, 2.14; 3.43 (-0.98, 7.85)	-8.65, -6.87; 1.78 (-5.65, 9.22)	9.36, 7.78; -1.58 (-8.24, 5.07)
Testosterone (nmol/l)	-0.34, -0.97; -0.63 (-2.52, 1.25)	0.86, -0.20; -1.06 (-2.58, 0.45)	0.38, -0.02; -0.40 (-2.66, 1.85)	0.44, 1.57; 1.12 (-1.20, 3.45)
Dehydroepiandrosterone sulphate (umol/l)	-0.13, 2.17; 2.30 (1.52, 3.08)	-0.50, 2.36; 2.86 (2.09, 3.64)	-0.15, 2.02; 2.16 (1.29, 3.04)	0.32, 1.41; 1.09 (0.43, 1.75)
Free thyroxine (pmol/l)	-0.36, 0.14; 0.50 (-0.63, 1.64)	5.32, 5.82; 0.51 (-0.43, 1.44)	4.33, 4.99; 0.65 (-0.45, 1.76)	3.77, 4.56; 0.79 (0.07, 1.52)
Thyroid stimulating hormone (mlU/l)	-0.02, 0.08; 0.10 (-0.22, 0.42)	0.12, -0.07; -0.20 (-0.58, 0.19)	0.52, 0.36; -0.15 (-0.54, 0.23)	0.09, 0.05; -0.04 (-0.46, 0.38)

Table S15: Absolute and relative frequencies of values below or above normal limits for laboratory safety variables in the safety set

Variable	LLN	ULN	Summary	Day 0		Day 7		Day 28		Day 35		Day 42	
				Placebo	AZD4017	Placebo	AZD4017	Placebo	AZD4017	Placebo	AZD4017	Placebo	AZD4017
Body mass index	N/A	N/A	Total, n	14	14					13	13		
Waist hip ratio	N/A	N/A	Total, n	14	14					13	13		
Systolic blood pressure	N/A	150	Total, n	14	14					13	13	13	14
			Above normal, n	3	2					4	1	3	
			Above normal, %	21	14					31	8	23	
Diastolic blood pressure	N/A	90	Total, n	14	14					13	13	13	14
			Above normal, n	2						1	1	2	3
			Above normal, %	14						8	8	15	21
HbA1c	N/A	41	Total, n	14	14	13	13	13	14	11	12	13	14
			Above normal, n	14	14	13	12	13	14	11	12	13	14
			Above normal, %	100	100	100	92	100	100	100	100	100	100
High-density lipoprotein	1.5	N/A	Total, n	14	14	14	14	13	14	12	12	12	13
			Below normal, n	12	12	12	14	11	14	10	12	10	10
			Below normal, %	86	86	86	100	85	100	83	100	83	77
Cholesterol	2.6	5.2	Total, n	14	14	14	14	13	14	12	12	12	13
			Below normal, n									1	
			Below normal, %									8	
			Above normal, n	2	1	2		1		1		1	
			Above normal, %	14	7	14		8		8		8	
Triglycerides	N/A	N/A	Total, n	14	14	14	14	13	14	12	12	12	13

Variable	LLN	ULN	Summary	Day 0		Day 7		Day 28		Day 35		Day 42	
				**Placebo**	**AZD4017**	**Placebo**	**AZD4017**	**Placebo**	**AZD4017**	**Placebo**	**AZD4017**	**Placebo**	**AZD4017**
Hemoglobin	114	160	Total, n	14	14	14	14	13	14	12	13	13	14
			Below normal, n	1				1		1		1	
			Below normal, %	7				8		8		8	
			Above normal, n	1	1	1	1		1		1		1
			Above normal, %	7	7	7	7		7		8		7
White blood cells	4	11	Total, n	14	14	14	14	13	14	12	13	13	14
			Below normal, n	1	1		1	1	1	1	1	1	1
			Below normal, %	7	7		7	8	7	8	8	8	7
			Above normal, n	1	1								1
			Above normal, %	7	7								7
Platelets	150	400	Total, n	14	14	14	14	13	14	12	13	13	14
			Below normal, n	1	1	2	1	1	1	1	1		1
			Below normal, %	7	7	14	7	8	7	8	8		7
			Above normal, n		1		2		2		2		2
			Above normal, %		7		14		14		15		14
Red blood cells	3.8	5.8	Total, n	14	14	14	14	13	14	12	13	13	14
			Below normal, n		1		1		1		1		1
			Below normal, %		7		7		7		8		7
			Above normal, n										1
			Above normal, %										7
Mean corpuscular volume	78	100	Total, n	14	14	14	14	13	14	12	13	13	14

Variable	LLN	ULN	Summary	Day 0		Day 7		Day 28		Day 35		Day 42	
				**Placebo**	**AZD4017**	**Placebo**	**AZD4017**	**Placebo**	**AZD4017**	**Placebo**	**AZD4017**	**Placebo**	**AZD4017**
			Below normal, n							1			
			Below normal, %							8			
			Above normal, n		1		2		1		1		1
			Above normal, %		7		14		7		8		7
Hematocrit (packed cell volume)	.37	.47	Total, n	14	14	14	14	13	14	12	13	13	14
			Below normal, n							1		1	1
			Below normal, %							8		8	7
			Above normal, n	3	1	2	2	1	1	1	2		2
			Above normal, %	21	7	14	14	8	7	8	15		14
Mean corpuscular hemoglobin	27	232	Total, n	14	14	14	14	13	14	12	13	13	14
			Below normal, n	2	2	2	2	1	2	1	2	1	3
			Below normal, %	14	14	14	14	8	14	8	15	8	21
			Above normal, n										
			Above normal, %										
Corpuscular hemoglobin concentration	N/A	N/A	Total, n	14	14	14	14	13	14	12	13	13	14
Red blood cell distribution width	11.5	15	Total, n	14	14	14	14	13	14	12	13	13	14
			Below normal, n										

Variable	LLN	ULN	Summary	Day 0		Day 7		Day 28		Day 35		Day 42	
				Placebo	AZD4017	Placebo	AZD4017	Placebo	AZD4017	Placebo	AZD4017	Placebo	AZD4017
			Below normal, %										
			Above normal, n	1	4	3	3	1	1	3	3	1	1
			Above normal, %	7	29	21	21	8	7	25	23	8	7
Albumin	35	50	Total, n	14	14	14	14	13	14	12	13	13	13
			Below normal, n					1					
			Below normal, %					8					
			Above normal, n										
			Above normal, %										
Bilirubin	2	21	Total, n	14	14	14	14	13	14	12	13	13	13
			Below normal, n										
			Below normal, %										
			Above normal, n				1		1	1			
			Above normal, %				7		7	8			
Alkaline phosphatase	30	130	Total, n	14	14	14	14	13	14	12	13	13	13
			Below normal, n						1		1		
			Below normal, %						7		8		
			Above normal, n	1		1						1	1
			Above normal, %	7		7						8	8
Alanine aminotransferase	N/A	40	Total, n	14	14	14	14	13	14	12	13	13	13
			Above normal, n		2		1		2		1		2
			Above normal, %		14		7		14		8		15

Variable	LLN	ULN	Summary	Day 0 Placebo	Day 0 AZD4017	Day 7 Placebo	Day 7 AZD4017	Day 28 Placebo	Day 28 AZD4017	Day 35 Placebo	Day 35 AZD4017	Day 42 Placebo	Day 42 AZD4017
Aspartate aminotransferase	N/A	40	Total, n	14	14	14	14	13	14	12	12	12	11
			Above normal, n										
			Above normal, %										
Gamma-glutamyl transpeptidase (males)	N/A	90	Total, n	12	10	12	10	11	10	10	9	11	9
			Above normal, n	1	1	1	1	1	1	1		1	
			Above normal, %	8	10	8	10	9	10	10		9	
Gamma-glutamyl transpeptidase (females)	N/A	50	Total, n	2	4	2	4	2	4	2	4	2	4
			Above normal, n	1		1	1	1		1		1	
			Above normal, %	50		50	25	50		50		50	
eGFR	90	N/A	Total, n	14	14	14	14	13	14	12	13	13	14
			Below normal, n	9	8	9	12	8	9	7	8	9	8
			Below normal, %	64	57	64	86	62	64	58	62	69	57
Sodium	133	146	Total, n	14	14	14	14	13	14	12	13	13	14
			Below normal, n										
			Below normal, %										
			Above normal, n		1					1			
			Above normal, %		7					8			
Potassium	3.5	5.3	Total, n	14	14	14	14	13	14	12	13	13	12
			Below normal, n										

Variable	LLN	ULN	Summary	Day 0		Day 7		Day 28		Day 35		Day 42	
				Placebo	AZD4017	Placebo	AZD4017	Placebo	AZD4017	Placebo	AZD4017	Placebo	AZD4017
			Below normal, %										
			Above normal, n		1	3			2			1	
			Above normal, %		7	21			14			8	
Urea	2.5	7.8	Total, n	14	14	14	14	13	14	12	13	13	14
			Below normal, n										
			Below normal, %										
			Above normal, n	3	5	4	5	3	4	2	7	3	5
			Above normal, %	21	36	29	36	23	29	17	54	23	36
Creatinine_M	64	104	Total, n	12	10	12	10	11	10	10	9	11	10
			Below normal, n	2	3	2	1	2	1	1	1	1	2
			Below normal, %	17	30	17	10	18	10	10	11	9	20
			Above normal, n		2	2	3	1	2	2	3	1	1
			Above normal, %		20	17	30	9	20	20	33	9	10
Creatinine_F	49	90	Total, n	2	4	2	4	2	4	2	4	2	4
			Below normal, n										
			Below normal, %										
			Above normal, n								1		1
			Above normal, %								25		25
Testosterone_M	8	30	Total, n	12	10	12	10	11	10	10	9	11	10
			Below normal, n	1	2	2	4	2	4	1	3	3	2
			Below normal, %	8	20	17	40	18	40	10	33	27	20

Variable	LLN	ULN	Summary	Day 0		Day 7		Day 28		Day 35		Day 42	
				Placebo	AZD4017	Placebo	AZD4017	Placebo	AZD4017	Placebo	AZD4017	Placebo	AZD4017
			Above normal, n							1			
			Above normal, %							10			
Testosterone_F	N/A	2.8	Total, n	2	4	2	4	1	4	2	4	2	4
			Above normal, n										
			Above normal, %										
Dehydroepiandrosterone sulphate (males)	3.6	13	Total, n	12	10	12	10	11	10	10	9	11	10
			Below normal, n	5	7	5	4	6	2	4	2	8	6
			Below normal, %	42	70	42	40	55	20	40	22	73	60
			Above normal, n				1						
			Above normal, %				10						
Dehydroepiandrosterone sulphate (females)	2.7	11	Total, n	2	4	2	4	2	4	2	4	2	4
			Below normal, n	2	3	2	1	2	2	2	2	2	3
			Below normal, %	100	75	100	25	100	50	100	50	100	75
			Above normal, n										
			Above normal, %										
Free thyroxine	10	20	Total, n	14	14	14	13	12	12	12	13	13	14
			Below normal, n										
			Below normal, %										
			Above normal, n				1						

Variable	LLN	ULN	Summary	Day 0		Day 7		Day 28		Day 35		Day 42	
				Placebo	AZD4017	Placebo	AZD4017	Placebo	AZD4017	Placebo	AZD4017	Placebo	AZD4017
			Above normal, %				8						
Thyroid-stimulating hormone	.2	4	Total, n	14	14	14	13	12	12	12	13	13	14
			Below normal, n										
			Below normal, %										
			Above normal, n										
			Above normal, %										

LLN, lower limit of normal; ULN, upper limit of normal.

Table S16: Sample sizes for future trials based on estimates from available case data in full analysis set

At an alpha of 0.05 (5% significance) and 1-beta of 0.90 (90% power), sample sizes for a range of substantive between-group differences are presented that allow for a 10% dropout rate. For TEWL and integrity variables, means and standard deviations (SDs) are presented on the log scale. For all variables, sample size is based on mean and SD (and Pearson's r where applicable), as specified in the statistical analysis plan. However, some variables were not normally distributed which may limit the accuracy of these estimates. Sample size estimates presented here should be considered preliminary.

Variable	Relative difference	Placebo mean	AZD4017 mean	Pooled SD	R*	N per arm
11bHSD1 activity radioassay (percent conversion per 24 hours): Day 28	10%	12.38	11.14	5.13	0.07	399
	20%	12.38	9.90	5.13	0.07	100
	30%	12.38	8.66	5.13	0.07	46
11bHSD1 activity ELISA (percent conversion per 24 hours): Day 28	10%	15.27	13.74	25.85	0.36	5821
	20%	15.27	12.22	25.85	0.36	1456
	30%	15.27	10.69	25.85	0.36	648
Sudomotor function Left Hand (μS): Day 35	10%	56.46	62.11	12.83	0.42	100
	20%	56.46	67.75	12.83	0.42	26
	30%	56.46	73.40	12.83	0.42	12
Sudomotor function Right Hand (μS): Day 35	10%	54.08	59.48	14.07	0.61	100
	20%	54.08	64.89	14.07	0.61	26
	30%	54.08	70.30	14.07	0.61	11
Sudomotor function Hands (μS): Day 35	10%	55.27	60.80	13.26	0.52	99
	20%	55.27	66.32	13.26	0.52	26
	30%	55.27	71.85	13.26	0.52	11
Sudomotor function Left Foot (μS): Day 35	10%	68.69	75.56	15.26	0.67	63
	20%	68.69	82.43	15.26	0.67	16
	30%	68.69	89.30	15.26	0.67	8
Sudomotor function Right Foot (μS): Day 35	10%	69.31	76.24	16.69	0.77	56
	20%	69.31	83.17	16.69	0.77	14

Variable	Relative difference	Placebo mean	AZD4017 mean	Pooled SD	R*	N per arm
	30%	69.31	90.10	16.69	0.77	7
Sudomotor function Feet (μS): Day 35	10%	69.00	75.90	15.82	0.72	59
	20%	69.00	82.80	15.82	0.72	14
	30%	69.00	89.70	15.82	0.72	7
Sudomotor function Overall (μS): Day 35	10%	62.13	68.35	12.82	0.70	50
	20%	62.13	74.56	12.82	0.70	13
	30%	62.13	80.78	12.82	0.70	6
Skin hydration (A.U): Day 35	10%	38.19	42.01	9.90	0.55	110
	20%	38.19	45.83	9.90	0.55	28
	30%	38.19	49.65	9.90	0.55	12
Epidermal thickness (μm): Day 35	10%	61.72	67.90	9.43	0.07	56
	20%	61.72	74.07	9.43	0.07	14
	30%	61.72	80.24	9.43	0.07	7
Wound gap diameter (mm): Day 2	10%	1.49	1.34	0.71	N/A	529
	20%	1.49	1.19	0.71	N/A	132
	30%	1.49	1.04	0.71	N/A	59
Wound depth (mm): Day 7	10%	0.60	0.54	0.20	N/A	262
	20%	0.60	0.48	0.20	N/A	66
	30%	0.60	0.42	0.20	N/A	30
Wound gap diameter (mm): Day 30	10%	1.44	1.30	0.60	N/A	409
	20%	1.44	1.16	0.60	N/A	102
	30%	1.44	1.01	0.60	N/A	46
Wound depth (mm): Day 35	10%	0.60	0.54	0.19	N/A	233
	20%	0.60	0.48	0.19	N/A	59
	30%	0.60	0.42	0.19	N/A	27
Hour 3 TEWL (Set 1; Day 0)	10%	3.55	3.45	0.24	0.25	119
	20%	3.55	3.33	0.24	0.25	27
	30%	3.55	3.20	0.24	0.25	10
Hour 48 TEWL (Set 1; Day 2)	10%	2.99	2.88	0.34	0.32	220
	20%	2.99	2.76	0.34	0.32	50
	30%	2.99	2.63	0.34	0.32	20
Hour 168 TEWL (Set 1; Day 7)	10%	2.61	2.50	0.55	0.40	541
	20%	2.61	2.38	0.55	0.40	121
	30%	2.61	2.25	0.55	0.40	48
Hour 3 TEWL (Set 2; Day 28)	10%	3.17	3.06	0.41	0.12	351

Variable	Relative difference	Placebo mean	AZD4017 mean	Pooled SD	R*	N per arm
	20%	3.17	2.95	0.41	0.12	79
	30%	3.17	2.81	0.41	0.12	31
Hour 48 TEWL (Set 2; Day 30)	10%	2.67	2.56	0.41	0.26	329
	20%	2.67	2.45	0.41	0.26	73
	30%	2.67	2.31	0.41	0.26	29
Hour 168 TEWL (Set 2; Day 35)	10%	2.30	2.20	0.40	0.17	334
	20%	2.30	2.08	0.40	0.17	74
	30%	2.30	1.95	0.40	0.17	29
Hour 0 TEWL (Set 3; Day 35)	10%	1.94	1.84	0.42	-0.12	374
	20%	1.94	1.72	0.42	-0.12	84
	30%	1.94	1.59	0.42	-0.12	33
Number of tapes required for barrier disruption: Day 28	10%	3.63	3.73	0.49	0.42	500
	20%	3.63	3.82	0.49	0.42	138
	30%	3.63	3.90	0.49	0.42	66

*Correlation with baseline value (not applicable for wound healing measures)
