# Supplementary material for: Oral 11β-HSD1 inhibitor AZD4017 improves wound healing and skin integrity in adults with type 2 diabetes mellitus: a pilot randomized controlled trial
Source: Eur J Endocrinol. 2022 Feb 3;186(4):441–55. doi: 10.1530/EJE-21-1197 (PMC8942338; doi:10.1530/EJE-21-1197)

Figure S1. Correlation between the different methods of assessing 24-hour 11β-HSD1 activity (percent conversion per 24 hours) in the full analysis set

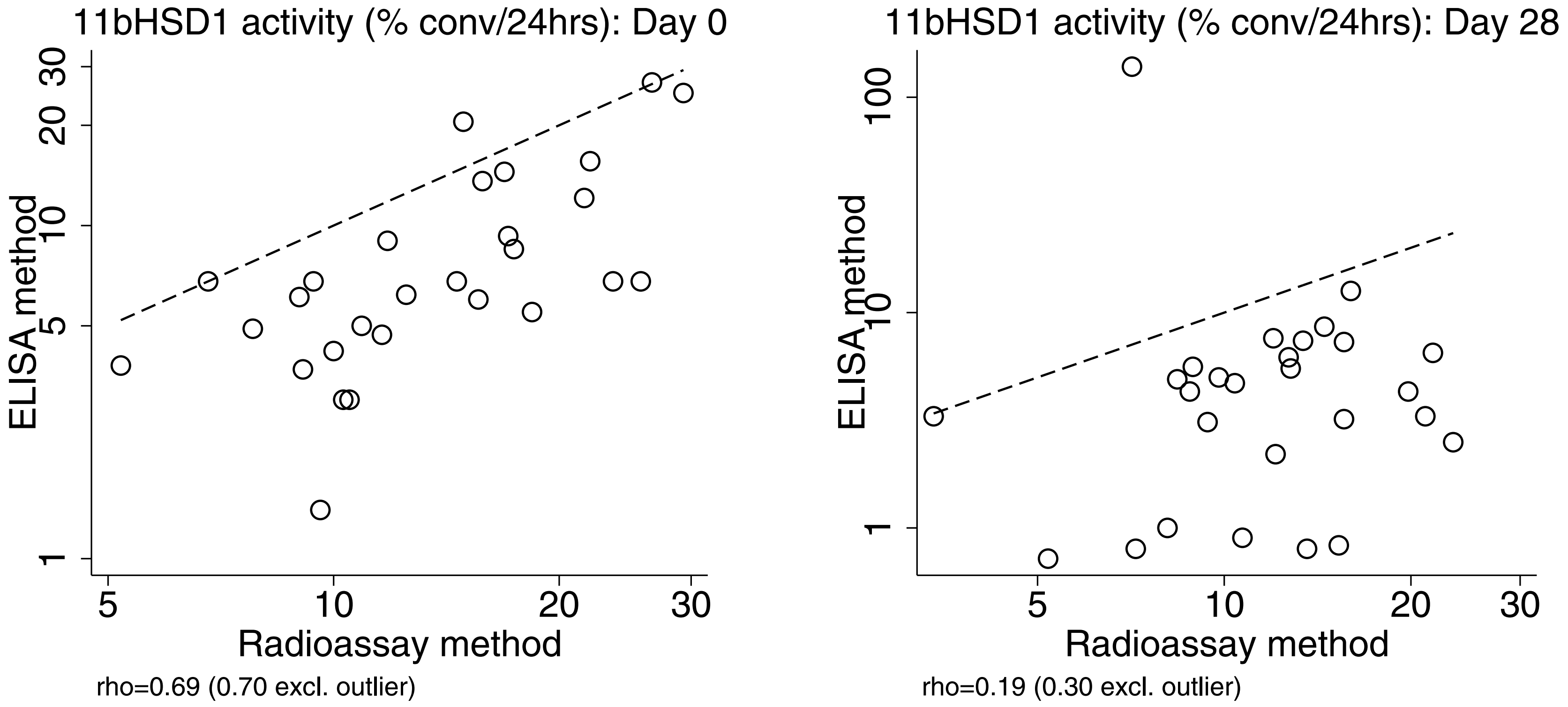

Figure S2. Correlation between AZD4017 levels in the biopsy at day 28 and in plasma at day 35 in the full analysis set (AZD4017 group only)

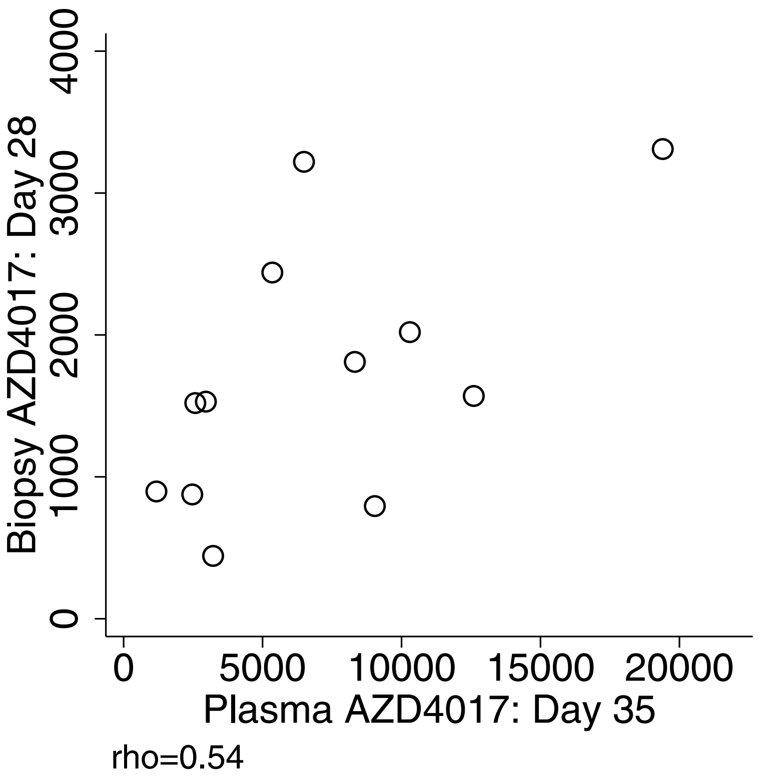

Supplement: Figure S1. Correlation between the different methods of assessing 24-hour 11β-HSD1 activity (percent conversion per 24 hours) in the full analysis set [file supplementary_figure_1.pdf]
